# Analysis of the fecal and oral microbiota in chronic recurrent multifocal osteomyelitis

**DOI:** 10.1186/s13075-021-02711-8

**Published:** 2022-02-22

**Authors:** Philipp Rausch, Meike Hartmann, John F. Baines, Philipp von Bismarck

**Affiliations:** 1 Institute for Clinical Molecular Biology (IKMB), Kiel University, Kiel, Germany; 2 Laboratory of Genomics and Molecular Biomedicine, Department of Biology, University of Copenhagen, Copenhagen, Denmark; 3 Clinic for General Pediatrics, University Hospital Schleswig-Holstein, Kiel, Germany; 4 Max Planck Institute for Evolutionary Biology, Plön, Germany; 5 Institute for Experimental Medicine, Kiel University, Kiel, Germany

**Keywords:** Chronic recurrent multifocal osteomyelitis, CRMO, CMO, Chronic multifocal osteomyelitis, CNO, Chronic non-bacterial osteomyelitis, HACEK, Microbiome, Pediatric bone inflammation

## Abstract

**Background:**

Chronic recurrent multifocal osteomyelitis (CRMO) is a rare autoinflammatory bone disease for which a lack of bacterial involvement is a key diagnostic feature to distinguish it from other symptomatically related diseases. However, the growing evidence suggesting an involvement of the host-associated microbiota in rheumatic disorders together with the now wide accessibility of modern culture-independent methods warrant a closer examination of CRMO.

**Methods:**

In this study, we show through bacterial 16S rRNA gene profiling that numerous features of the oral- and fecal microbial communities differentiate children with and without CRMO.

**Results:**

Notably, communities in diseased children are characterized by a lack of potential probiotic bacteria in the fecal community and an overabundance of known pathobionts in the oral microbial communities. Of special interest is the HACEK group, a set of commonly known oral pathogens that are implicated in the development of several acute and chronic diseases such as osteitis and rheumatoid arthritis. Furthermore, we observe that gut bacterial communities in the diseased children appear to reflect an altered host physiology more strongly than the oral community, which could suggest an oral disease origin followed by propagation and/or responses beyond the oral cavity.

**Conclusions:**

Bacterial communities, in particular the oral microbiota, may serve as an indicator of underlying susceptibility to CRMO, or play a yet undefined role in its development.

**Supplementary Information:**

The online version contains supplementary material available at 10.1186/s13075-021-02711-8.

## Background

Chronic recurrent multifocal osteomyelitis was first described by Giedion et al. [1] and is ascribed to the very heterogeneous disease pattern of non-bacterial osteomyelitis [2], affecting mainly children and adolescents. The cause of this disease complex remains unknown, although chronicity, recurrence, and involvement of the innate immune system speak for an origin in the auto-inflammatory disease spectrum [3, 4]. A sterile bone inflammation (culture negative blood sample or bone biopsy) is the hallmark of auto-inflammatory bone disorders with clinical representations ranging from a mostly mild and limited unifocal disease such as chronic non-bacterial osteomyelitis (CNO) to severe diseases with multifocal chronic active and recurrent inflammation known as chronic recurrent multifocal osteomyelitis (CRMO) [1]. Classic clinical signs are pain in the affected bones, sometimes swelling or warmth and erythema, and rarely fever [5]. In a few cases, chronic bone inflammation is associated with inflammatory skin disease, psoriasis, palmoplantar pustulosis, or acne fulminans and in 10% of CRMO cases inflammatory bowel diseases can occur [6]. Specific laboratory parameters do not exist and often no or only very mild changes in the known inflammation parameters (CRP and BSR/ESR) are observed. The inflammation can be self-limiting and is primarily localized in the metaphyses of tubular long bones, but can occur in every bone, including the clavicle, mandible, and vertebrae [7]. In most cases, chronic osteomyelitis does not lead to changes in the macroscopic bone structure, but occasionally bone thickening (hyperostosis) and spinal involvement can occur [8]. Histologically, the affected bones show immune cell infiltration and bone resorption processes, but also a partly exceeding formation of bone material. Disease patterns with simultaneous occurrence of acne fulminans and arthritis are very close to the SAPHO disease complex (synovitis, acne, pustulosis, hyperostosis, osteitis), from which CRMO is believed to be the pediatric equivalent based on shared inflammatory features [9, 10]. However, CRMO is a low incidence disease with only 0.43 cases per 100,000 children (chronic non-bacterial osteomyelitis) and predominantly occurs in girls [11].

An immediate cause of CRMO and a central pillar of its pathogenesis appears to be an imbalance of pro-inflammatory monocytes (increased expression of IL-1β, IL6, TNFα) and reduced levels of anti-inflammatory cytokines (IL10) [12]. Genetic factors are also implicated, including a potential risk locus for CRMO on chromosome 18 [13], observed disease clustering among family groups, and high- and low-IL10 expression promotor haplotypes [14, 15]. The activation of typical bacterial sensors like *TLR2* and *TLR4* may also be involved in the signaling cascade, which leads to reduced IL10 expression, dysregulated immune reactions, and eventually bone damage via *RANK* activation and osteoclast formation [6, 16].

Although a critical feature of CRMO is the apparent lack of a specific infectious cause, there are a number of indications for potential microbial involvement, not as a primary infection, but as triggers or as contributing factors. First, an established mouse model of osteomyelitis carries a genetic risk locus known as *Pstpip2* (Proline-serine-threonine phosphatase-interacting protein 2) [17], whose human ortholog *PSTPIP1 (CD2BP1*) is an autosomal dominant cause of the hereditary autoinflammatory PAPA syndrome (pyogenic arthritis, pyoderma gangrenosum, acne) [18], a disease characterized by autoinflammatory bone inflammation [12, 19]. *PSTPIP1* also lies within the genomic region of a previously described CRMO risk locus in humans [13]. The murine *Pstpip2*^*cmo*^ model shows spontaneous osteomyelitis, which resembles the CRMO phenotype in humans. Interestingly, this CRMO model displays evidence of interaction with the microbiota, as *Pstpip2*^*cmo*^ mice show a marked increase of intestinal *Prevotella* abundance, which can be modified by dietary and antibiotic intervention with direct disease ameliorating effects (e.g*.*, decreasing IL-1β levels) [20]. So far CRMO and related comorbidities have been treated successfully by the administration of IL-1-Receptor antagonists like *anakinra*, emphasizing the importance of IL-1β and neutrophil activation [21–26]. Furthermore clear associations/comorbidities with autoinflammatory diseases like psoriasis or inflammatory bowel disease were reported, which speak for an overlap in genetic and/or environmental risk factors, including potential bacterial triggers [5, 27]. Similarly, the association of host innate immune receptors *TLR2* and *TLR4* in CRMO pathogenesis [14, 28] or *NLRP3* [29] imply microbial involvement. Finally, recent data suggest that mast cells appear to be an additional factor in sterile bone inflammation [30]. For inflammatory bowel disease, it was shown that mast cell activities altered by the host microbiome probably play a role in its pathogenesis [31].

Although living bacteria were thus far not isolated from biopsies or blood samples of CRMO patients, it remains possible that yet unknown slow growing and/or fastidious bacteria could be undetected triggers and not necessarily restricted to the site of inflammation [32]. Thus, microbial community composition may hold valuable diagnostic information as in other inflammatory disorders such as inflammatory bowel disease [33] and could represent a therapeutic target. We here describe the oral and fecal microbiota in a panel of CRMO patients and matched controls derived from Northern Germany, which reveals numerous disease-related signatures at both body sites and implicates several bacterial taxa with known pathogenic properties.

## Methods

### Human sample collection and clinical data

Fecal and saliva samples were taken with consent over the course of regular clinical control examinations in the pediatric rheumatology outpatient clinic at the “Klinik für Kinder- und Jugendmedizin I”, Universitätsklinikum Schleswig Holstein, Campus Kiel (UKSH, Kiel campus, Germany; ethical permit: D 438/13). All procedures related to patients and healthy subjects followed the guidelines of the Declaration of Helsinki. For an overview of cohort specific meta-data, like age, gender, physiological measurements, and medication, see Table 1. The inclusion of the patients in the study did not require a predefined time of disease or treatment history. We recruited 25 CRMO patients in clinical remission, defined as having no ailment with or without treatment at the time of sampling, and a group of 24 healthy children matched by age and gender with no evidence of inflammatory disease as controls.

**Table 1:** The table shows an overview of cohort characteristics of healthy and diseased study participants, as well as the respective number of fecal and oral samples. It also shows the range of clinical measurements available for the diseased study participants. Clinical measures range from the concentration of C-reactive protein (CRP mg/l), percentage of monocytes, percentage of lymphocytes, percentage of basophils, percentage of eosinophils, percentage of neutrophils, thrombocytes (cells/nl), number of erythrocytes (cellsp/l), to AP (U/l, alkaline phosphatase), LDH (U/l, lactate dehydrogenase), calcium concentration (mmol/l), hematocrit, MCV (fl, mean cell volume), MCH (pg/cell, mean corpuscular hemoglobin), MCHC (g/dl, mean corpuscular hemoglobin concentration), hemoglobin concentration (g/dl; Hb), and BSR 1h (1 hour blood sedimentation rate). The table includes the normal physiological ranges of the respective clinical measures based on UKSH Clinical Chemistry Department and Oster [34]

Cohort	Category/variable	Normal range	Fecal			Oral		
			Ratio or mean	±SD	#NA	Ratio or mean	±SD	#NA
All	F/M	-	25/16	-	1	28/18	-	1
	Healthy/CRMO	-	20/22	-	0	23/24	-	0
	Age	-	12.307	3.315	1	12.485	3.325	2
Healthy	F/M	-	11/8	-	1	13/9	-	1
	Age	-	12.271	3.419	1	12.658	3.474	2
CRMO	F/M	-	14/8	-	0	15/9	-	0
	Relapse (Yes/No)	-	8/14	-	0	9/15	-	0
	Age	-	12.340	3.302	0	12.334	3.256	0
	NSAID (Yes/No)	-	13/9	-	0	14/10	-	0
	Methylprednisolon (Yes/No)	-	10/12	-	0	12/12	-	0
	Cox 2 (Yes/No)	-	14/8	-	0	15/9	-	0
	Etanercept (Yes/No)	-	2/20	-	0	2/22	-	0
	Cortison (Yes/No)	-	5/17	-	0	7/17	-	0
	Sulfasalazin (Yes/No)	-	4/18	-	0	4/20	-	0
	Bisphosphonate (Yes/No)	-	2/20	-	0	2/22	-	0
	MTX (Yes/No)	-	1/21	-	0	2/22	-	0
Physiology	BSR (1h)^b^	7.00–12.00	34.524	19.717	1	34.682	19.256	2
(CRMO)	CRP (mg/l)^a^	< 5.00	16.382	18.892	0	15.646	18.248	0
	Hemoglobin/Hb (g/dl)^a^	10.70–13.90	12.164	1.164	0	12.179	1.114	0
	Erythrocytes (cells/pl)^a^	3.85–5.15	4.597	0.294	0	4.605	0.283	0
	Hematocrit/Hkt^b^	5.00–40.00	35.909	3.022	0	35.958	2.896	0
	MCV (fl)^a^	74.00–89.00	77.273	4.590	0	77.292	4.408	0
	MCH (pg/cell)^a^	24.50–31.00	26.510	1.727	1	26.470	1.655	1
	MCHC (g/dl)^a^	31.00–36.00	33.957	1.464	1	33.939	1.398	1
	Thrombocytes (cells/nl)^a^	200.00–445.00	343.909	66.273	0	344.792	65.939	0
	Neutrophils (cells/ml) in %^a^	26.00–75.00	53.245	8.431	2	53.355	8.628	2
	Eosinophils (cells/ml) in %^a^	0.50–5.50	2.860	1.979	2	2.795	1.933	2
	Basophils (cells/ml) in %^a^	< 1.76	0.389	0.285	3	0.381	0.273	3
	Lymphocytes (cells/ml) in %^a^	25.00–55.00	34.710	7.340	2	34.605	8.006	2
	Monocytes (cells/ml) in %^a^	1.50–8.50	8.132	2.304	3	8.257	2.241	3
	Calcium (mmol/l)^a^	2.20–2.70	2.395	0.062	2	2.392	0.059	2
	AP (U/l)^a^	142.00–335.00	178.167	61.045	10	179.692	58.705	11
	LDH (U/l)^a^	107.00–314.00	188.500	25.792	2	186.955	26.245	2

^a^Reference values/range based on the UKSH Clinical Chemistry Department

^b^Reference values/range based on Oster [34]

Fecal and saliva samples were collected after written consent, following the manufacturer’s instructions (PSP Saliva Gene DNA Kit, PSP Spin Stool DNA Kit; STRATEC Molecular GmbH, Germany) and stored in stabilizing solution at −20°C. As part of regular examinations, we assessed several clinical blood parameters, including among others the concentration of C-reactive protein (CRP mg/l) (see Table 1, Figure S1).

### DNA extraction and 16S rRNA gene sequencing

DNA was extracted from 47 saliva and 42 stool samples from 49 individuals using the PSP Saliva Gene DNA Kit and PSP Spin Stool DNA Kit respectively following the manufacturer’s protocol (STRATEC Molecular GmbH, Germany). The 16S rRNA gene was amplified using uniquely barcoded primers flanking the V1 and V2 hypervariable regions (27F-338R) with fused MiSeq adapters and sequencing was performed on the Illumina MiSeq platform with v3 chemistry. See supplemental methods for additional details.

### Sequence processing and quality control

FASTQ files were filtered and merged via *USEARCH* v8.0.1623 [35] and *FASTX tool* v0.0.13 [36]. Chimeric sequences were removed [35, 37] and sequences were classified and confirmed as bacterial using the *RDP classifier* and the RDP16 database in *mothur* 1.39.5 [38, 39]. For all downstream analyses of diversity and habitat association, we took a random subset of 13641 sequences per sample to normalize the read distribution. OTU binning was performed by the *opticlust* algorithm to ensure high quality OTU clusters [40]. Further details can be found in the supplemental methods.

### Statistical methods

Species diversity indices (ACE approximated species richness, Shannon-Weaver number equivalent) were calculated in *R* 3.3.2 using *vegan* [41–44]. For beta diversity analyses, we included OTU-based metrics that highlight shared presence (Jaccard distance) or abundance (Bray-Curtis distance). Distances were visualized via principal coordinate analysis (PCoA) and centroids and vectors were fit via an iterative goodness of fit test. Testing for community separation between health conditions or by age was performed via distance-based redundancy analyses and permutative ANOVA [45, 46]. Univariate analyses were carried out with negative binomial generalized linear models and linear models using prior model selection by AIC criterion and likelihood ratio tests in *MASS* [47] and permutative Wilcoxon tests as implemented in *coin* [48]. Indicator species analysis (*IndVal.g*) was used to assess the predictive value of a taxon for each respective host phenotype/category as implemented in *indicspecies* [49]. *P*-values of the genera and OTU associations were adjusted by the Benjamini-Hochberg procedure [50]. Additionally, machine learning via RandomForest was employed for supervised classification and derivation of important taxa to distinguish healthy individuals and CRMO patients [51, 52].

Evaluating correlations between bacterial taxa and clinical parameters was done using the Euclidean distance correlation [53], where only associations with *P* ≤ 0.010 were chosen for further analysis, as this method is able to detect a wide variety of correlations. Further, *SparCC* was used to generate co-abundance networks of consensus genera and species level OTUs in fecal and oral communities at a *P*-value cutoff of *P*_FDR_ ≤ 0.05 and ║*R*║ ≥ 0.20 [54]. Node-based importance measures (degree, betweenness, PageRank-index) were calculated as implemented in *igraph* [55–57] and significance of importance was obtained via a one-sided *Z*-test against a set of permuted networks (10,000 permutations).

## Results

### Cohort description

We recruited 49 individuals (25 CRMO patients, 24 healthy controls) and collected fecal and saliva samples. A summary of patient characteristics is given in Table 1. Two patients were enrolled in the study at the time of diagnosis, before the start of treatment. In 22 patients, the collection of stool and saliva samples was between 1 and 90 months after first diagnosis. Eight of these patients had no treatment at the time of sample collection; 17 patients were under therapy with an NSAID. Seven patients were given corticosteroids, one was given Etanercept, and two were treated with bisphosphonates during their course of disease and prior to the time of sample collection. All patients were previously diagnosed with CRMO, with signs of active bone inflammation (clinical signs of inflammation, inflammation biomarkers, detection of bone inflammation via MRI), but at the time of sampling were in a state of clinical remission without signs of discomfort or ailment with or without treatment. Thus, with the exception of BSR, CRP, monocyte, and basophile number, most clinical parameters showed no clear deviation from their respective normal reference ranges (see Table 1; Figure S1) [34].

In total, we successfully sequenced the V1/V2 region of the bacterial 16S rRNA gene in 42 fecal samples (20 healthy, 22 diseased) and 47 oral samples (23 healthy, 24 diseased). This resulted in a sampling depth of 13641 sequences per sample, corresponding to a Good’s coverage at the species level (97% sequence identity OTUs) of 0.9498 ± 0.0264 (fecal) and 0.9876 ± 0.0049 (oral; mean ± sd). Throughout the subsequent analyses, the potential influence of anti-inflammatory treatment, which varied among patients, was tested in all instances where feasible/appropriate (i.e., for taxon abundances and alpha- and beta diversity analyses).

### Differential phylum abundances between controls and CRMO patients

**Fig. 1:**
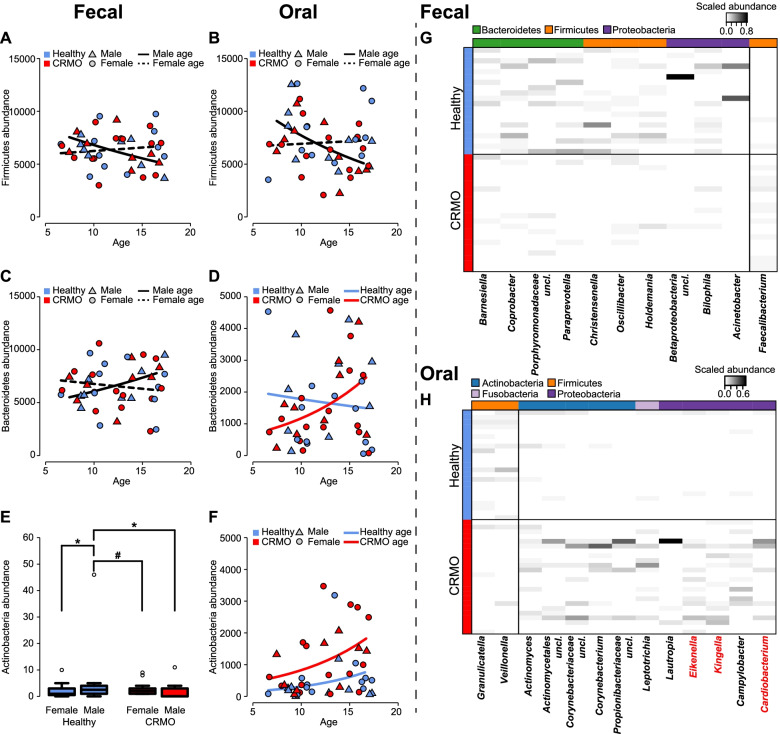
Abundance differences of the three most abundant phyla in the fecal (**A**, **C**, **E**) and oral microbiome (**B**, **D**, **F**). Shown are the results according to the best statistical model (see Table 2). Heatmaps show the distribution of indicator genera significantly associated to CRMO patients or healthy control individuals based on indicator species analysis of fecal (**G**) and oral microbial communities (**H**, Table 3) [49]

In the primary analysis, we investigated the influence of patient characteristics (Table 1) on the abundance of the major taxonomic groups in the fecal and oral microbiome. Sex and age are the main contributors to abundance differences in the major phyla of the fecal microbiota, such as Firmicutes and Bacteroidetes. Actinobacteria are also influenced by sex and are underrepresented among CRMO patients (see Fig. 1A–F; Table 2). In the oral communities, Firmicutes are influenced by age and sex, while Bacteroidetes show a slight abundance increase in CRMO patients compared to healthy controls, which increases with age. However, oral Actinobacteria and Fusobacteria significantly increase in samples of diseased children with age (Fig. 1F and Figure S2C & S2D; Table 2). Members of the phylum Proteobacteria in the fecal and oral microbial communities do not show any association to health condition, age, or sex in our study (Figure S2A & S2B; Table 2).

**Table 2:** Results of negative binomial generalized linear model analyses of major phyla abundances in fecal and oral communities (see Fig. 1 and Figure S2 for illustration)

Data	Phylum	Model	DF	Deviance	Residual deviance	*P*-value	Pairwise comp.	*P(Z)*
Stool	Bacteroidetes	Age	1,39	0.22075	44.517	0.63847	-	-
		Sex	1,38	0.01076	44.506	0.91737	-	-
		Age:Sex	1,37	2.78322	41.723	0.09526	-	-
	Firmicutes	Age	1,39	0.3794	45.373	0.53792	-	-
		Sex	1,38	0.0748	45.298	0.78450	-	-
		Age:Sex	1,37	3.8370	41.461	0.05013	-	-
	Proteobacteria	~1	40	-	46.840	-	-	-
	Actinobacteria	Health	1,39	1.8799	48.404	0.17035	CRMO(F)-Healthy(F)	0.6286
		Sex	1,38	1.2938	47.111	0.25536	Healthy(M)-Healthy(F)	0.0368
		Health:Sex	1,37	3.4262	43.684	0.06417	CRMO(M)-Healthy(F)	0.9130
							Healthy(M)-CRMO(F)	0.0782
							CRMO(M)-CRMO(F)	0.5810
							CRMO(M)-Healthy(M)	0.0431
	Fusobacteria	Health	1,39	1.3877	23.715	0.23880	-	-
		Age	1,38	0.0282	23.687	0.86662	-	-
		Sex	1,37	0.0014	23.685	0.97024	-	-
		Heatlh:Age	1,36	3.4243	20.261	0.06424	-	-
		Health:Sex	1,35	4.4391	15.822	0.03513	-	-
Saliva	Bacteroidetes	Health	1,43	0.0277	53.793	0.86782	-	-
		Age	1,42	0.6094	53.183	0.43501	-	-
		Health:Age	1,41	2.8931	50.290	0.08896	-	-
	Firmicutes	Age	1,43	1.6254	50.914	0.20235	-	-
		Sex	1,42	0.2719	50.642	0.60204	-	-
		Age:Sex	1,41	4.6660	45.976	0.03077	-	-
	Proteobacteria	~1	44	-	47.510	-	-	-
	Actinobacteria	Health	1,43	8.6775	57.643	0.00322	-	-
		Age	1,42	5.7823	51.861	0.01619	-	-
	Fusobacteria	Health	1,43	7.7296	76.532	0.00543	-	-
		Age	1,42	2.9008	73.631	0.08854	-	-
		Sex	1,41	0.1865	73.444	0.66587	-	-
		Health:Age	1,40	13.1673	60.277	0.00028	-	-
		Age:Sex	1,39	8.6147	51.662	0.00333	-	-

### Bacterial associations with disease state

A closer inspection of taxon associations to CRMO disease state reveals many interesting genus- and species-level associations, which mainly overlap. Among the healthy children, several potential probiotic bacteria are overrepresented in feces including several members of the Bacteroidales (*Paraprevotella*, *Coprobacter*, *Porphyromonadaceae*, *Barnesiella*) and others (Fig. 1G and Figure S2E; Table 3 and Table S1). Interestingly, *Christensenella*, a bacterium shown to be highly heritable and previously shown to be associated with decreased weight gain [58], is overrepresented in the fecal samples of healthy children. Surprisingly, *Faecalibacterium*, which is a taxon typically positively associated with a healthy status in the context of other inflammatory disorders such as inflammatory bowel disease [59], rather shows an association to CRMO, similar to another member of the *Clostridia* Cluster *Fusicatenibacter* (see Fig. 1G and Figure S2E; Table 3 and Table S1).

**Table 3:** Indicator genera analysis of fecal and oral microbial communities for healthy and diseased individuals. Potential HACEK-group members are highlighted in **bold** face and genera marked with “*” show repeated associations

Data	Factor	Genus classification (RDP16)	Assoc.	*IndVal.g*	*P*-Value	*P*_FDR_
Stool	Disease	*Faecalibacterium*	CRMO	0.79720	0.00250	0.16123
		Barnesiella*	Healthy	0.79559	0.03250	0.46579
		*Coprobacter*		0.71171	0.02110	0.46435
		*Porphyromonadaceae uncl.*		0.69602	0.02880	0.46435
		*Paraprevotella*		0.67131	0.00200	0.16123
		*Christensenella*		0.57721	0.01070	0.34504
		Oscillibacter*		0.80668	0.04010	0.46693
		*Holdemania*		0.79469	0.00980	0.34504
		*Betaproteobacteria uncl.*		0.53721	0.02720	0.46435
		*Bilophila*		0.75734	0.02600	0.46435
		*Acinetobacter*		0.44721	0.04220	0.46693
	Relapse	*Clostridium XVIII*	Yes	0.84863	0.02530	0.45555
		Barnesiella*	No	0.86570	0.02210	0.45555
		*Odoribacter*		0.85980	0.02680	0.45555
		*Alistipes*		0.91034	0.00220	0.22438
		*Anaerovorax*		0.88385	0.00710	0.24138
		Oscillibacter*		0.89888	0.00620	0.24138
Saliva	Disease	*Actinomyces*	CRMO	0.83715	0.04220	0.40217
		*Actinomycetales uncl.*		0.75191	0.03880	0.40217
		*Corynebacteriaceae uncl.*		0.52099	0.03290	0.40217
		*Corynebacterium*		0.93276	0.00510	0.15936
		*Propionibacteriaceae uncl.*		0.84285	0.00020	0.01250
		*Leptotrichia*		0.89057	0.00990	0.18212
		*Lautropia*		0.93432	0.01970	0.30778
		***Eikenella***		0.81943	0.00230	0.09582
		***Kingella***		0.91866	0.00010	0.01250
		*Campylobacter*		0.87207	0.01020	0.18212
		***Cardiobacterium***		0.84092	0.00760	0.18212
		*Granulicatella*	Healthy	0.82050	0.02750	0.38191
		*Veillonella*		0.83492	0.04830	0.40217
	Relapse	-	-	-	-	-

Next, we focused on abundance differences in oral bacteria according to disease status, whereby *Veillonella*, *Granullicatella*, members of the Bacteroidales (*Prevotellaceae*, *Porphyromonadaceae*), and others are associated to healthy subjects (Fig. 1H and Figure S2F). *Granulicatella* is a common member of the oral microbial community known to cause nosocomial infections and depends on metabolic byproducts of other bacteria [60]. Similarly *Veillonella* is a common fastidious and mostly mutualistic bacterium of the human oral and gut microbiota that rarely causes disease [61–63]. In contrast, oral bacteria associated to CRMO include among others *Actinomyces*, *Cardiobacterium, Campylobacter*, *Eikenella*, *Leptotrichia*, and *Kingella*. *Eikenella* is known to cause osteomyelitis and endocarditis particularly in immunocompromised individuals [64–66]. *Kingella* was also recently recognized as a widely distributed and important pathogen for children, with disease manifestations in joints and bones [67]. *Haemophilus*, *Aggregatibacter*, *Cardiobacterium*, *Eikenella*, *and Kingella* together comprise *the* HACEK group (*Haemophilus parainfluenzae*, *Aggregatibacter actinomycetemcomitans*, *Aggregatibacter aphrophilus*, *Aggregatibacter paraphrophilus*, *Cardiobacterium* spp., *Eikenella corrodens*, *Kingella* spp.; see Fig. 1H and Figure S2F; Table 3 and Table S1), an assembly of slowly growing, fastidious bacteria that are often involved in unusual infections with cardiac complications, as well as strong periodontal and skeletal involvement [68–70]. Bacteria grouped as *unclassified Pasteurellaceae* were included in the HACEK group, due to the difficult to resolve taxonomy among members of this taxonomic group, e.g., *Haemophilus* and *Aggregatibacter* [71]. Furthermore, *Actinomyces* and *Leptotrichia* were reported to cause oral abscesses, systemic inflammation, bacteremia, and even bone degradation [72–74]. Additional supervised classification of disease states via RandomForest further validated many of the indicator species and genera, including several members of the HACEK group, which strongly differentiate CRMO patients and healthy individuals (see Table S2) [51].

### Taxon co-abundance network analysis highlights the importance of opportunistic pathobionts

**Fig. 2:**
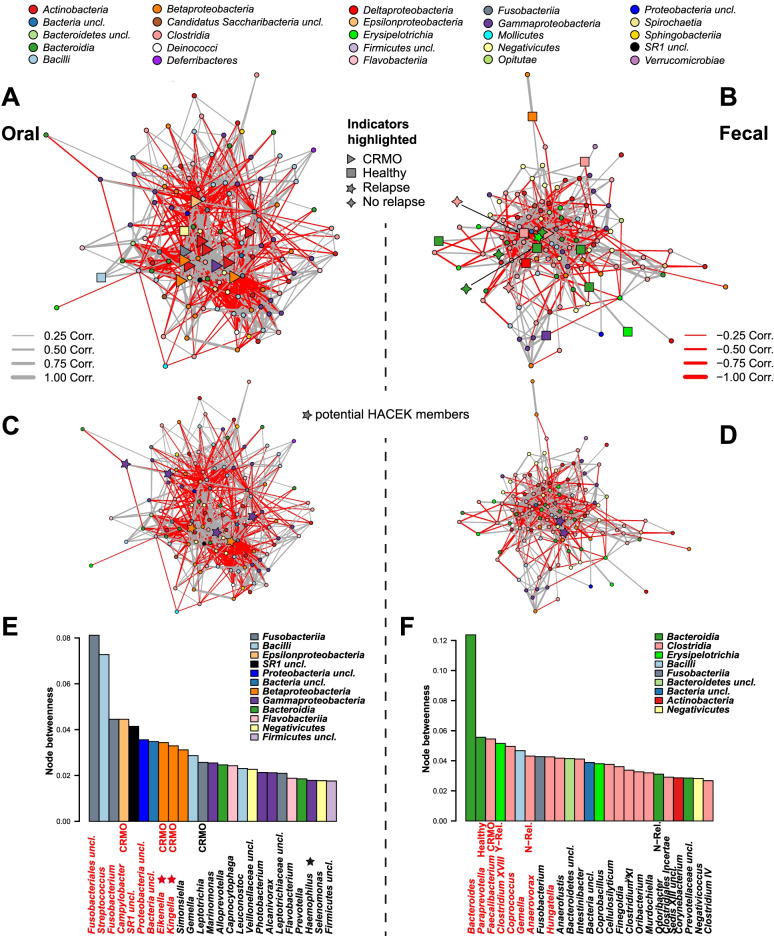
Co-abundance networks of consensus genera in (**A**) oral and (**B**) fecal microbial communities based on SparCC correlation [54]. Node colors indicate class level taxonomy, the edge width and color between nodes show the strength and direction of the bacterial correlations. Node shapes highlight indicator genera [49] for a healthy or CRMO-associated microbial community (■—healthy, ▲—CRMO), or bacteria associated to disease relapse (★—relapse, ✦—no relapse). Networks (**C**) and (**D**) highlight the position of genera belonging to the HACEK group in the co-abundance networks (★—HACEK). Barplots show the top 30 most central bacteria in oral (**E**) and fecal (**F**) bacterial consensus genus networks (node betweenness; see Table S3). Bar colors indicate bacterial classification at the class level. Names highlighted in red indicate higher than average centrality values according to network permutation tests. Additionally, significant indicator species associations are added above the names and potential HACEK membership is indicated next to genus names (/STAR/)

Through the analysis of genus and species level co-abundance networks (Fig. 2A–F; Figure S3, S4, S5), we identified highly interactive groups of oral and fecal bacteria, whose abundances are closely correlated and may have important community wide and potentially disease-modifying effects. Indicator bacteria for CRMO (Fig. 2A, B; OTU level: Figure S4A-S4D) are on average more highly interconnected than other bacteria, as determined by their number of “interaction partners” (node degree; fecal-genera: *P*=0.9822, oral-genera: *P*=0.0005, fecal-OTU: *P*<0.0001, oral-OTU: *P*<0.0001; Wilcoxon test), but also by their positional importance, specifically in the oral community network (PageRank; fecal-genera: *P*=0.2168, oral-genera: *P*=0.0010, fecal-OTU: *P*<0.0001, oral-OTU: *P*<0.0001; Wilcoxon test). Similarly, a higher relative number of connections among HACEK bacteria (Fig. 2C, D; OTU level: Figure S4E & S4F) compared to others is observed (node degree; fecal-genera: *P*=0.1733, oral-genera: *P*=0.05131, fecal-OTU: *P*=0.6716, oral-OTU: *P*=0.0049; Wilcoxon test) as well as a higher positional importance specifically in the oral communities (PageRank; fecal-genera: *P*=0.0185, oral-genera: *P*=0.09001, fecal-OTU: *P*=0.6914, oral-OTU: *P*=0.0391; Wilcoxon test). By permuting the network nodes in an untargeted approach, we identified a significantly higher than average taxon importance of, among others, indicator genera and species for CRMO in the oral community (i.e., *Kingella*, *Capnocytophaga*, *Campylobacter*, *Eikenella*), as well as several other potentially pathogenic bacteria like *Fusobacteria* or *Streptococcus* (Fig. 2E; Figure S3A & 3C; OTU level: Figure S5A, C, D; Table S3 and S4). In contrast, several of the most important bacteria in the fecal co-abundance networks are indicator bacteria associated to a healthy condition or absence of disease relapse, with the exception of *Faecalibacterium* (associated to CRMO; see Fig. 2F; Figure S3B & S3D; OTU level: Figure S5B, S5E, S5F; Table S3 and S4). These analyses imply for one, a central role of the detected indicator taxa in the respective communities, but also the importance of disease-associated bacteria, in particular members of the HACEK group, in the oral microbial communities.

### Bacterial associations to clinical parameters

The host-associated microbiome is known to be intimately associated to a broad range of physiological and immune parameters [75]. Thus, given the availability of clinical measurements for CRMO patients, we conducted further exploratory analyses to gain further insight in this group. Interestingly, several consensus genera and OTUs in the fecal and oral samples show strong and mostly directionally consistent associations to clinical data in CRMO patients (Tables S5, S6, S7), although clinical measures are mainly in the normal and age-appropriate physiological range (Figure S1). As an example, fecal OTUs belonging to the *uncl. Ruminococcaceae* mostly positively correlate with BSR, CRP, MCHC, counts of eosinophils, neutrophils, and thrombocytes, while consistently negatively correlate to levels of hemoglobin, hematocrit and MCH, and counts of erythrocytes and lymphocytes. Particularly, indicator bacteria for health or disease show a similar direction of association with the respective clinical measure. In the fecal samples, OTUs belonging to the *unclassified Lachnospiraceae* and *Faecalibacterium* are associated with decreased calcium levels as well as being indicators for CRMO (see Table S6), while indicators of healthy individuals in the Clostridiales positively associate to MCH, neutrophils, and BSR. Health-associated fecal Proteobacteria are positively correlated to MCH and the levels of eosinophils, but negatively correlated to lymphocyte counts (Table S6). An indicator in feces for having experienced disease relapse (OTU-697: *uncl. Ruminococcaceae*) is also positively associated to CRP levels, and negatively to hemoglobin concentration and hematocrit. In contrast, indicators of no relapse show opposing associations to hematocrit, erythrocytes, and thrombocytes (e.g., OTU-1104: *Faecalibacterium*; OTU-2811: *Lachnospiraceae uncl.*).

Oral indicator species for CRMO are associated with MCHC (OTU-512: *Kingella*; OTU-835: *Cardiobacterium*), lymphocyte number (OTU-255: *Campylobacter*), and LDH activity (OTU-548: *uncl. Prevotellaceae*) (Table S7). In general, most members of the HACEK group display a comparable directionality of their association to clinical parameters, although with heterogeneity at the OTU level (e.g., see Table S6). This separation between potentially pathogenic or potentially probiotic bacterial groups in the way they collectively correlate to the clinical measures implies a similar relationship to host physiology among those bacterial groups.

### Community diversity and its relationship to disease condition

**Fig. 3:**
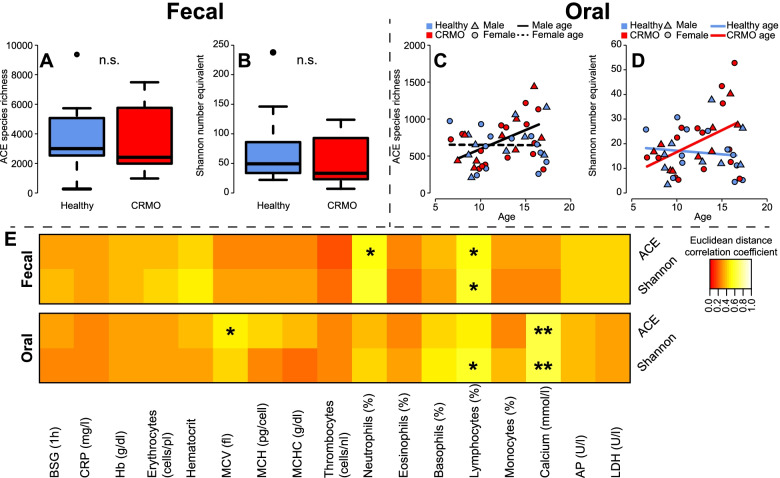
Alpha diversity analysis based on their bacterial species richness (A, C; ACE), abundance distribution, and dominance (B, D; Shannon H (number equivalent)). Shown are the illustrations of the best final models (see Table 3; n.s. *P*>0.05). **E** The heatmaps show correlations (Euclidean distance correlation [53]) of ACE species richness and Shannon diversity (numbers equivalent) in fecal and oral microbial communities with clinical measures in CRMO patients (significant correlations highlighted by * *P*≤0.05, ** *P*≤0.01, *** *P*≤0.001; see Table S8)

To characterize community complexity, we employed different and complementary measures of alpha diversity based on the OTU presence and abundance distribution (ACE species richness, Shannon Entropy number equivalent) [44, 76] (Fig. 3). We could not find differences in alpha diversity measures derived from fecal communities, according to health status, age, or gender of the sampled individuals (Fig. 3A, B; Table 4). Interestingly, the oral microbial communities showed a pronounced association with subject age, as communities appear to become more diverse with age, particularly in male subjects (Fig. 3C). Further, the oral communities of CRMO patients become more diverse with increasing subject age, as compared to the healthy controls (Fig. 3D; Table 4). The treatment of CRMO patients had no significant effect on any measure of alpha diversity in our patient cohort, in contrast to a recently published study (fecal- ACE: *Z*=0.8208, *P*=0.4446; Shannon Entropy: *Z*=0.8208, *P*=0.4410; oral- ACE: *Z*=0.0869, *P*=0.9546; Shannon Entropy: *Z*=−0.5504, *P*=0.6049; Wilcoxon test) [77]. Whether patients suffered a disease relapse since their diagnosis also did not show an influence on community diversity among patients (fecal- ACE: *Z*=−1.2285, *P*=0.2372; Shannon Entropy: *Z*=−1.6381, *P*=0.1057; oral- ACE: *Z*=−0.0894, *P*=0.9501; Shannon Entropy: *Z*=0.0298, *P*=1.0000; Wilcoxon test). However, among CRMO patients, we also identified significant associations between clinical measurements and alpha diversity, which are mostly restricted to either the fecal or oral microbial community (Fig. 3E; Table S8). Lymphocyte number decreases with a more diverse community (ACE species richness, Shannon Entropy) in the gut microbial communities and with increasing Shannon diversity in the oral microbial communities. In contrast, the number of neutrophils increases with increasingly diverse fecal communities (ACE species richness, Shannon Entropy). Only in the oral microbial community did increasing diversity correlate with increasing blood calcium levels (Table S8).

**Table 4:** Results of alpha diversity analyses via linear models, focusing on OTU-based diversity measures (ACE species richness, Shannon entropy (number equivalent)) in fecal and oral samples

Sample	Diversity	Model factors	DF	*F*-value	*P*-value
Stool	ACE species richness	~1	-	-	-
	Shannon (number equivalent)	~1	-	-	-
Saliva	ACE species richness	Age	1,41	2.6082	0.11399
		Sex	1,41	0.6894	0.41117
		Age:Sex	1,41	4.3243	0.04387
	Shannon (number equivalent)	Health	1,41	1.9378	0.17142
		Age	1,41	2.4224	0.12730
		Health:Age	1,41	4.7187	0.03567

### Community differentiation between disease conditions

**Fig. 4:**
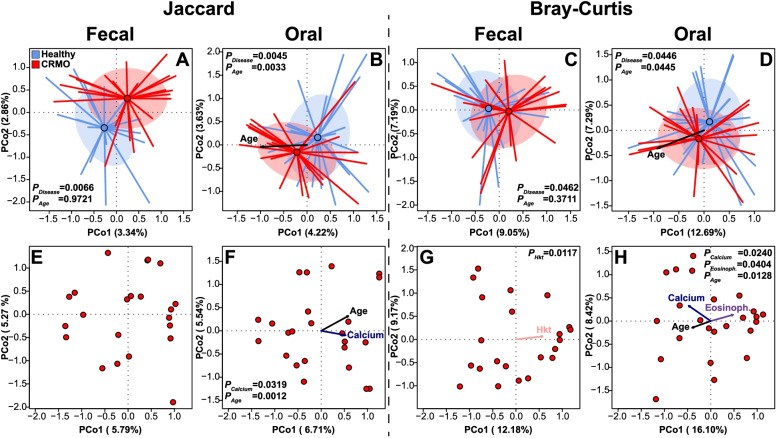
Principal coordinate analyses of fecal and oral bacterial community spectra, focusing on the first two dimensions based on the shared presence (Jaccard, **A**, **B**) and shared abundance of bacteria among individuals (Bray-Curtis, **C**, **D**) highlighting compositional differences with respect to disease in fecal samples (Jaccard: *F*_1,40_=1.0621, *P*=0.0066, *R*^2^=0.0259, adj. *R*^2^=0.0015; Bray-Curtis: *F*_1,40_=1.2848, *P*=0.0462, *R*^2^=0.0311, adj. *R*^2^=0.0069) and oral samples (Jaccard: *F*_1,45_=1.1673, *P*=0.0045, *R*^2^=0.0253, adj. *R*^2^=0.0036; Bray-Curtis: *F*_1,45_=1.4153, *P*=0.0446, *R*^2^=0.0305, adj. *R*^2^=0.0089). The age vector highlights the direction of significant community changes with respect to subject age in oral samples (Jaccard: *F*_1,43_=1.1760, *P*=0.0033, *R*^2^=0.0266, adj. *R*^2^=0.0040; Bray-Curtis: *F*_1,43_=1.4168, *P*=0.0445, *R*^2^=0.0319, adj. *R*^2^=0.0094), while no significant correlations are detectable among fecal samples (Jaccard: *F*_1,39_=0.9721, *P*=0.9626, *R*^2^=0.0243, adj. *R*^2^=-0.0007; Bray-Curtis: *F*_1,39_=1.0220, *P*=0.3711, *R*^2^=0.0255, adj. *R*^2^=0.0005). **E**–**H** PCoAs visualize the direction and relative strength of significant correlations of physiological parameters in CRMO patients, with presence/absence (**E**, **F**) and abundance (**G**, **H**) based measures of fecal (**E**, **G**) and oral (**F**, **H**) microbial communities (see Table S9). Vectors were fitted via the *envfit* routine for visualization

To characterize differences between microbial communities, we used beta diversity measures based on qualitative and quantitative differences in OTU composition (Jaccard [J], Bray-Curtis [BC]). This revealed significant differences in structure and composition between healthy and diseased subjects in both fecal and oral microbial communities (Fig. 4A–D). A correlation of community differences with age was exclusive to the oral microbial communities, similar to what we observed in the alpha diversity of oral communities. This implies that the composition of the oral microbial community also changes successively with age (Fig. 4B, D). Further, we could also identify significant correlations between clinical measurements and community differences in the fecal- and oral microbial communities of CRMO patients (Fig. 4E–H). Calcium concentration and eosinophil number are exclusively correlated to differences in the oral microbial communities (Fig. 4F, H). In contrast, only hematocrit is correlated to community differences in the fecal microbial communities of CRMO patients, further highlighting the different influence of the fecal and oral microbial communities on patient physiology (see Table S9; Fig. 4E, G). However, we failed to identify any significant community differentiation between patients with or without a previous disease relapse (fecal- J: *F*_1,20_=1.0205, *P*=0.1420; BC: *F*_1,20_=1.0615, *P*=0.2961; oral- J: *F*_1,22_=0.9732, *P*=0.6868; BC: *F*_1,22_=0.9793, *P*=0.4472). To further account for potential influences of anti-inflammatory treatment, we performed analyses between treated and untreated individuals, for which no influence on fecal or oral communities was detected (fecal- J: *F*_1,20_=0.9843, *P*=0.6001; BC: *F*_1,20_=1.0642, *P*=0.3469; oral- J: *F*_1,22_=0.8851, *P*=0.9336; BC: *F*_1,22_=0.3966, *P*=0.9961).

## Discussion

In this study, we identify a number of intriguing microbial signatures in CRMO, including significant signals of disease manifestation and a spectrum of indicator species that were previously found to be associated with other diseases, such as rheumatoid arthritis (RA) [78]. The reduced diversity in the feces of diseased individuals and somewhat opposing community dynamics in the oral community (increased diversity) suggests potentially higher oral colonization in diseased individuals, possibly driven by reduced colonization resistance. Further, the numerous associations between clinical parameters and community composition and complexity among CRMO patients indicate possible interactions relevant to disease development and progression.

Several other aspects of the microbiota between CRMO patients and controls are, of note, particularly the consistent and significant compositional differences of fecal and oral microbiota between healthy controls and CRMO patients, which were not yet reported for this disease. These patterns are another clear sign of a community wide influence of CRMO on the microbiota, or vice versa. Upon characterizing these differences to the individual taxonomic level in the fecal microbiota, we find an overrepresentation of potentially probiotic bacteria among controls such as *Barnesiella* or Clostridial groups (*Cluster IV* / *Clostridium leptum group*, *Cluster XIVa*), which are well-known butyrate producers with anti-inflammatory effects [59, 79, 80]. However, *Faecalibacterium* strain competition (multiple species level OTUs are observed in this cohort) can negatively influence the spectrum of short chain fatty acids, which might explain the association of this usually beneficial bacterial group to CRMO, which is similar to what was observed in cases of atopic dermatitis or juvenile idiopathic arthritis [81, 82].

In contrast, oral taxa indicative of CRMO such as *Corynebacterium*, *Actinomyces*, *Propionibacteria*, and *Leptotrichia* are generally associated to nosocomial infections, which can develop into local and systemic inflammation with skeletal manifestations. Infections by *Corynebacterium*, *Actinomyces*, and *Propionibacteria* in particular show similar etiologies (e.g., actinomycosis like symptoms: swelling, suppuration, abscess, fibrosis) and are known for associations to bacteremia, endocarditis, systemic inflammation, and even bone degradation [72–74, 83]. Although we must emphasize that no indication of infection was present in our CRMO patient cohort, associations involving the HACEK group (*Haemophilus parainfluenzae*, *Aggregatibacter actinomycetemcomitans*, *A.aphrophilus*, *A.paraphrophilus*, *Cardiobacterium spp.*, *Eikenella corrodens*, *Kingella* spp*.*) are among the most interesting findings of our study. HACEK members are slow growing fastidious bacteria mainly of oral origin and involved in unusual infections with cardiac and skeletal complications [68–70], including children with inflammation in joints and bones [67]. *Eikenella* in particular is known to cause osteomyelitis and endocarditis if it is able to invade the respective body sites [64–66]. Further, *Aggregatibacter* was shown to induce prostaglandin, *TNFα*, and *IL-6* expression [84], which are central drivers of CRMO pathogenesis [12]. The concerted occurrence of HACEK members and their close correlation are strong signs that this bacterial assembly has a stable co-occurrence pattern, with potential synergistic interactions with other bacteria, and is relevant to disease. Interestingly, slow growing fastidious bacteria have been suspected to trigger or associate with CNO/CRMO, but were not yet detected in biopsies of diseased individuals via culture or PCR [32, 85]. Although antibiotic treatment has no significant influence on CRMO progression [27], most HACEK members are resistant against several commonly used antibiotics, which may contribute to the missing effect of antibiotics on CRMO [86, 87].

Bone marrow-derived hematopoietic cells were identified as highly associated to CMO/CRMO [88]. Neutrophils in particular appear to be a causative agent in animal models and humans acting via (pro-)IL-1β-related pathways [6, 20, 88] and are further associated to the microbial community in our analyses. Experimental ablation of neutrophils confers significant protection from disease progression and tissue damage in *Pstpip2*^cmo^ mice [20]. It has been reported that LPS leads to a stronger inflammasome activation (*NLRP3*) and subsequently a stronger imbalance between pro-inflammatory (increase in *IL-20*) and anti-inflammatory signals (reduced *IL-1*, *IL-10* expression) in CRMO patient samples [14, 29]. Thus, oral activation of the inflammasome via bacteria, like members of the HACEK group or Fusobacteria, may lead to a systemic induction or amplification of inflammatory processes in CRMO [89–91]. However, there is also evidence for inflammasome independent inflammation in the *Pstpip2*^cmo^ mouse model for CRMO [88]. Accordingly, members of the fecal microbiota mainly correlate negatively to AP activity, which is in stark contrast to the oral communities, which appear to increase in regard to AP activity and blood calcium levels, potentially signifying inflammatory processes [92]. In the fecal communities, we observe a lack of potential beneficial bacteria, while an increase of potential pathobionts is observed in the oral communities in CRMO cases. Thus, it is possible that a lack of beneficial bacterial signals (e.g., SCFAs) in the gut together with increasing the reactivity/inflammatory potential of the oral microbiota may result in some children having a higher probability to develop or sustain systemic autoinflammatory processes (i.e., CRMO).

In our study, the concentration of various immune cell types such as lymphocytes, monocytes, basophiles, and eosinophils and characteristics of erythrocytes also correlate to structural characteristics and single members of the microbial communities and thus indicate a modulating effect of the microbiota on the immune system of children with CRMO. For CRP, we identified positive correlations with oral *Rothia*, while fecal *Ruminococcaceae*, *Lachnospiraceae*, and others associate with increased CRP. Interestingly, the HACEK bacteria group was also implicated in a metagenomic study of RA in Chinese individuals [78], where they were abundant in healthy controls, but correlated negatively with certain clinical disease markers such as C-reactive protein (CRP) and RA-specific autoantibodies (anti-CCP). Indicator taxa also differ in their association to disease like *Holdemania* (Zhang: RA associated, current: health associated), *Leptotrichia* (Zhang: control associated, current: CRMO associated), while others overlap (e.g., *Rothia*-control associated, *Campylobacter*-control associated, *Actinomyces*-disease associated). Another notable group identified in our study is *Prevotella*, which was previously identified in the context of a mouse model of CNO by Lukens et al. [20]. In the current study, fecal and oral *Prevotella/Prevotellaceae* OTUs associate to healthy individuals. The oral *Prevotellaceae* OTU displays a positive correlation to LDH activity CRMO patients. *Prevotella* is a heterogeneous group with a range of different metabolic capacities [93, 94], and may contain genomic variation changing their physiological and pathological behavior in relation to their host and disease status [20, 95].

Although CRMO fundamentally differs from RA in many underlying principles of pathophysiology, where RA is a classical autoimmune disease in comparison to CRMO, which is autoinflammatory, our results display interesting overlap with recent studies of RA. Specifically, *Aggregatibacter actinomycetemcomitans* as a representative of the HACEK group was found to lead to the hypercitrullination of autoantigens in RA, linking this disease to periodontal infection with this bacterium [96]. With regard to CRMO, *Aggregatibacter* is known to disrupt neutrophil membrane integrity via Leukotoxin A (LtxA) and thereby reduces and dysregulates neutrophils, a cell population under strong suspicion to be a causative agent for autoinflammation in CRMO animal models via (pro-)IL-1β [20, 88]. Another HACEK member identified in this study, *Cardiobacterium*, was shown to increase IL-1β levels [97], as well as *Actinomyces* [98] and *Propionibacteria* (now *Cutibacterium*) [99], which is often isolated “concomitantly” with HACEK bacteria and is associated to the related SAPHO syndrome, although controversially discussed [100]. Thus, HACEK bacteria, *Corynebacterium*, *Actinomyces*, *Propionibacteria*, and *Leptotrichia* may together form a disease-modulating consortium, and their single and/or combined disease associations may lead to skeletal manifestations [83, 98, 101, 102]. For other autoinflammatory diseases, links between dysbioses of the gut microbiome and disease activity were also previously discussed. Individual authors report that clinical symptoms in Familial Mediterranean Fever, a classic autoinflammatory syndrome, are influenced by an altered microbiome [103, 104]. However, Ozen et al. failed to provide evidence of microbiome alterations in an international study population. Further, gut microbial dysbiosis in systemic juvenile idiopathic arthritis (Still syndrome) is also suspected to be associated to the health condition and disease activity in different cohorts [82, 105].

Despite the numerous significant observations of our study, it suffers from a number of limitations. First, the patient cohort was sampled at different time points over the course of disease, leading some samples to be from treated and others from untreated individuals. Although we failed to observe significant differences between these subgroups at several levels of the analysis, this heterogeneity could mask some disease-related signals. Further, due to the relative small sample size of this rare disease, we could not further distinguish between the types of anti-inflammatory therapy.

## Conclusions

In conclusion, our culture-independent analysis of microbial communities draws new attention to the potential role of members of the microbiota in the pathology of CRMO. We reveal a clear signal of known pathogenic groups such as HACEK or *Actinomyces*. Importantly, however, these observations may not reflect a direct causal role, but could also be a consequence of other primary microbial community alterations and/or host immunological responses, resulting in an imbalance of cytokine expression [6]. Thus, future studies employing longitudinal sampling strategies, larger sample sizes, and deeper molecular and phenotypic characterization of patients and controls are warranted to better understand the etiology of CRMO.

## Supplementary Information

**Additional file 1:.** Supplemental methods. Supplemental Figures S1–S5. Supplemental Tables S1–S9.

## Data Availability

The datasets generated and analyzed (raw sequencing data, meta data) during the current study are available at the European Nucleotide Archive (https://www.ebi.ac.uk/ena/) under the accession number PRJEB22984 or ERP104714.

## Abbreviations

16S rRNA: 16S ribosomal RNA
AIC: Akaike Information Criterion
AP: Alkaline phosphatase
BC: Bray-Curtis dissimilarity
BSR/ESR: Blood sedimentation rate (1 hour) / Erythrocyte sedimentation rate
CMO: Chronic multifocal osteomyelitis
CNO: Chronic non-bacterial osteomyelitis
CRMO: Chronic recurrent multifocal osteomyelitis
CRP: C-reactive protein
HACEK: Bacterial group consisting of *Haemophilus spp.*, *Aggregatibacter actinomycetemcomitans*, *Aggregatibacter aphrophilus*, *Aggregatibacter paraphrophilus*, *Cardiobacterium spp.*, *Eikenella corrodens*, *Kingella spp*
Hb: Hemoglobin
Hkt: Hematocrit
IFNγ: Interferon-γ
IL10: Interleukin 10
IL1-ß: Interleukin 1ß
IL6: Interleukin 6
J: Jaccard distance
LDH: Lactate dehydrogenase
MCH: Mean corpuscular hemoglobin
MCHC: Mean corpuscular hemoglobin concentration
MCV: Mean cell volume
MRI: Magnetic Resonance Imaging
NSAID: Non-steroidal anti-inflammatory drug
OTU: Operational taxonomic unit
PCoA: Principal Coordinate Analysis
RA: Rheumatoid arthritis
TLR: Toll-like receptor
TNFα: Tumor necrosis factor α

## References

[1] Giedion A, Holthusen W, Masel LF, Vischer D (1972). Subacute and chronic “symmetrical” osteomyelitis. Annales de Radiologie (Paris).

[2] Wipff J, Adamsbaum C, Kahan A, Job-Deslandre C (2011). Chronic recurrent multifocal osteomyelitis. Joint Bone Spine.

[3] Ferguson PJ, El-Shanti HI (2007). Autoinflammatory bone disorders. Curr Opin Rheumatol.

[4] Wagner AD, Andresen J, Jendro MC, Hülsemann JL, Zeidler H (2002). Sustained response to tumor necrosis factor α–blocking agents in two patients with SAPHO syndrome. Arthritis Rheum.

[5] Jansson A, Renner ED, Ramser J, Mayer A, Haban M, Meindl A (2007). Classification of non-bacterial osteitis: retrospective study of clinical, immunological and genetic aspects in 89 patients. Rheumatology (Oxford, England).

[6] Hofmann SR, Kapplusch F, Girschick HJ, Morbach H, Pablik J, Ferguson PJ (2017). Chronic recurrent multifocal osteomyelitis (CRMO): presentation, pathogenesis, and treatment. Curr Osteopor Rep.

[7] Andronikou S, Mendes da Costa T, Hussien M, Ramanan AV (2019). Radiological diagnosis of chronic recurrent multifocal osteomyelitis using whole-body MRI-based lesion distribution patterns. Clin Radiol.

[8] Vittecoq O, Ait Said L, Michot C, Mejjad O, Thomine J-M, Mitrofanoff P (2000). Evolution of chronic recurrent multifocal osteitis toward spondylarthropathy over the long term. Arthritis Rheum.

[9] Beretta-Piccoli BC, Sauvain MJ, Gal I, Schibler A, Saurenmann T, Kressebuch H (2000). Synovitis, acne, pustulosis, hyperostosis, osteitis (SAPHO) syndrome in childhood: a report of ten cases and review of the literature. Eur J Pediatr.

[10] Reith JD, Bauer TW, Schils JP (1996). Osseous manifestations of SAPHO (synovitis, acne, pustulosis, hyperostosis, osteitis) syndrome. Am J Surg Pathol.

[11] Voit A. Bakterielle osteomyelitis und Nichtbakterielle Osteitis(NBO): Eine vergleichende Datenerhebung zur Inzidenz und klinischen manifestation der beiden Erkrankungen im Kindesalter und zusätzliche Betrachtung des Langzeitverlaufs der NBO. Dissertation. Ludwig-Maximilians-Universität München. 2013.

[12] Hedrich CM, Hofmann SR, Pablik J, Morbach H, Girschick HJ (2013). Autoinflammatory bone disorders with special focus on chronic recurrent multifocal osteomyelitis (CRMO). Pediatr Rheumatol Online J.

[13] Golla A, Jansson A, Ramser J, Hellebrand H, Zahn R, Meitinger T (2002). Chronic recurrent multifocal osteomyelitis (CRMO): evidence for a susceptibility gene located on chromosome 18q21.3-18q22. Eur J Hum Genet.

[14] Hofmann SR, Schwarz T, Möller JC, Morbach H, Schnabel A, Rösen-Wolff A (2011). Chronic non-bacterial osteomyelitis is associated with impaired Sp1 signaling, reduced IL10 promoter phosphorylation, and reduced myeloid IL-10 expression. Clin Immunol.

[15] Hamel J, Paul D, Gahr M, Hedrich CM (2012). Pilot study: possible association of IL10 promoter polymorphisms with CRMO. Rheumatol Int.

[16] Takayanagi H (2007). Osteoimmunology: shared mechanisms and crosstalk between the immune and bone systems. Nat Rev Immunol.

[17] Ferguson PJ, Bing XY, Vasef MA, Ochoa LA, Mahgoub A, Waldschmidt TJ (2006). A missense mutation in pstpip2 is associated with the murine autoinflammatory disorder chronic multifocal osteomyelitis. Bone.

[18] Wise CA, Gillum JD, Seidman CE, Lindor NM, Veile R, Bashiardes S (2002). Mutations in CD2BP1 disrupt binding to PTP PEST and are responsible for PAPA syndrome, an autoinflammatory disorder. Hum Mol Genet.

[19] Smith EJ, Allantaz F, Bennett L, Zhang D, Gao X, Wood G (2010). Clinical, molecular, and genetic characteristics of PAPA syndrome: a review. Curr Genom.

[20] Lukens JR, Gurung P, Vogel P, Johnson GR, Carter RA, McGoldrick DJ (2014). Dietary modulation of the microbiome affects autoinflammatory disease. Nature.

[21] Majeed HA, Al-Tarawna M, El-Shanti H, Kamel B, Al-Khalaileh F (2001). The syndrome of chronic recurrent multifocal osteomyelitis and congenital dyserythropoietic anaemia. Report of a new family and a review. Eur J Pediatr.

[22] Herlin T, Fiirgaard B, Bjerre M, Kerndrup G, Hasle H, Bing X (2013). Efficacy of anti-IL-1 treatment in Majeed syndrome. Ann Rheum Dis.

[23] Aksentijevich I, Masters SL, Ferguson PJ, Dancey P, Frenkel J, van Royen-Kerkhoff A (2009). An autoinflammatory disease with deficiency of the interleukin-1–receptor antagonist. N Engl J Med.

[24] Eleftheriou D, Gerschman T, Sebire N, Woo P, Pilkington CA, Brogan PA (2010). Biologic therapy in refractory chronic non-bacterial osteomyelitis of childhood. Rheumatology.

[25] Colina M, Pizzirani C, Khodeir M, Falzoni S, Bruschi M, Trotta F (2010). Dysregulation of P2X7 receptor-inflammasome axis in SAPHO syndrome: successful treatment with anakinra. Rheumatology.

[26] Rech J, Manger B, Lang B, Schett G, Wilhelm M, Birkmann J (2012). Adult-onset Still’s disease and chronic recurrent multifocal osteomyelitis: a hitherto undescribed manifestation of autoinflammation. Rheumatol Int.

[27] Borzutzky A, Stern S, Reiff A, Zurakowski D, Steinberg EA, Dedeoglu F (2012). Pediatric chronic nonbacterial osteomyelitis. Pediatrics.

[28] Hofmann SR, Morbach H, Schwarz T, Rösen-Wolff A, Girschick HJ, Hedrich CM (2012). Attenuated TLR4/MAPK signaling in monocytes from patients with CRMO results in impaired IL-10 expression. Clin Immunol.

[29] Hofmann S, Kubasch A, Rösen-Wolff A, Girschick H, Morbach H, Hedrich C (2015). Altered expression of IL-10 family cytokines in CRMO result in enhanced inflammasome activation. Pediatr Rheumatol Online J.

[30] Young S, Sharma N, Lee JH, Chitu V, Neumeister V, Sohr E, Stanley ER, Hedrich CM, Craig AWB. Mast cells enhance sterile inflammation in chronic nonbacterial osteomyelitis. Dis Model Mech. 2019;12(8):1–9. 10.1242/dmm.040097 PMC6737947 31416928

[31] De Zuani M, Dal Secco C, Frossi B (2018). Mast cells at the crossroads of microbiota and IBD. Eur J Immunol.

[32] Jurik AG (2004). Chronic recurrent multifocal osteomyelitis. Semin Musculoskelet Radiol.

[33] Pascal V, Pozuelo M, Borruel N, Casellas F, Campos D, Santiago A (2017). A microbial signature for Crohn's disease. Gut.

[34] Oster O, Hoffmann GF, Lentze MJ, Spranger J, Zepp F (2015). Pädiatrisch relevante Referenzwerte: klinische Chemie. Pädiatrie: Grundlagen und praxis.

[35] Edgar RC (2010). Search and clustering orders of magnitude faster than BLAST. Bioinformatics.

[36] Hannon G: FASTX-Toolkit. In. http://hannonlab.cshl.edu/fastx_toolkit; 2010.

[37] Edgar RC, Haas BJ, Clemente JC, Quince C, Knight R (2011). UCHIME improves sensitivity and speed of chimera detection. Bioinformatics.

[38] Wang Q, Garrity GM, Tiedje JM, Cole JR (2007). Naive Bayesian classifier for rapid assignment of rRNA sequences into the new bacterial taxonomy. Appl Environ Microbiol.

[39] Schloss PD, Westcott SL, Ryabin T, Hall JR, Hartmann M, Hollister EB (2009). Introducing mothur: open source, platform-independent, community-supported software for describing and comparing microbial communities. Appl Environ Microbiol.

[40] Westcott SL, Schloss PD. OptiClust, an improved method for assigning amplicon-based sequence data to operational taxonomic units. mSphere 2017;2(2):1–11. 10.1128/mSphereDirect.00073-17 PMC5343174 28289728

[41] Team RC (2016). R: a language and environment for statistical computing. R Foundation for Statistical Computing. 3.3.2.

[42] Oksanen J, Blanchet FG, Kindt R, Legendre P, O'Hara RB, Simpson GL (2011). Vegan: community ecology package.

[43] Chao A (1987). Estimating the population size for capture-recapture data with unequal catchability. Biometrics.

[44] Jost L (2006). Entropy and diversity. Oikos.

[45] Legendre P, Legendre L (1998). Numerical ecology. Second English edition. Dev Environ Model.

[46] Legendre P, Anderson MJ (1999). Distance-based redundancy analysis: testing multispecies responses in multifactorial ecological experiments. Ecol Monogr.

[47] Venables WN, Ripley BD (2002). Generalized linear models. Modern applied statistics with S.

[48] Hothorn T, Hornik K, Van de Wiel MA, Zeileis A (2006). A Lego system for conditional inference. Am Stat.

[49] De Cáceres M, Legendre P, Moretti M (2010). Improving indicator species analysis by combining groups of sites. Oikos.

[50] Benjamini Y, Hochberg Y (1995). Controlling the false discovery rate: a practical and powerful approach to multiple testing. J Roy Stat Soc B Met.

[51] Breiman L (2001). Random forests. Mach Learn.

[52] Kuhn M (2008). Building predictive models in R using the caret package. J Stat Softw.

[53] Székely GJ, Rizzo ML, Bakirov NK (2007). Measuring and testing dependence by correlation of distances. Ann Stat.

[54] Friedman J, Alm EJ (2012). Inferring correlation networks from genomic survey data. PLoS Comput Biol.

[55] Csardi G, Nepusz T (1695). The igraph software package for complex network research. InterJournal 2006, complex systems.

[56] Brin S, Page L (1998). The anatomy of a large-scale hypertextual web search engine. Comput Netw Isdn Syst.

[57] Freeman LC (1979). Centrality in social networks conceptual clarification. Soc Networks.

[58] Goodrich JK, Waters JL, Poole AC, Sutter JL, Koren O, Blekhman R (2014). Human genetics shape the gut microbiome. Cell.

[59] Sokol H, Pigneur B, Watterlot L, Lakhdari O, Bermúdez-Humarán LG, Gratadoux J-J (2008). Faecalibacterium prausnitzii is an anti-inflammatory commensal bacterium identified by gut microbiota analysis of Crohn disease patients. Proc Natl Acad Sci.

[60] Collins MD, Lawson PA (2000). The genus Abiotrophia (Kawamura et al.) is not monophyletic: proposal of Granulicatella gen. Nov., Granulicatella adiacens comb. nov., Granulicatella elegans comb. nov. and Granulicatella balaenopterae comb. nov. Int J Syst Evol Microbiol.

[61] Bhatti MA, Frank MO (2000). Veillonella parvula meningitis: case report and review of Veillonella infections. Clin Infect Dis.

[62] Mashima I, Nakazawa F (2014). The influence of oral Veillonella species on biofilms formed by streptococcus species. Anaerobe.

[63] Ng SK, Hamilton IR (1971). Lactate metabolism by Veillonella parvula. J Bacteriol.

[64] Swisher LA, Roberts JR, Glynn MJ (1994). Needle lickers osteomyelitis. Am J Emerg Med.

[65] Chen CK, Wilson ME (1992). Eikenella corrodens in human oral and non-oral infections: a review. J Periodontol.

[66] Patrick WD, Brown WD, Ian Bowmer M, Sinave CP (1990). Infective endocarditis due to Eikenella corrodens: case report and review of the literature. Can J Infect Dis.

[67] Principi N, Esposito S (2015). Kingella kingae infections in children. BMC Infect Dis.

[68] Ellner JJ, Rosenthal MS, Lerner PI, McHenry MC (1979). Infective endocarditis caused by slow-growing, fastidious. Gram-negative bacteria. Medicine.

[69] Yew HS, Chambers ST, Roberts SA, Holland DJ, Julian KA, Raymond NJ (2014). Association between HACEK bacteraemia and endocarditis. J Med Microbiol.

[70] Chambers ST, Murdoch D, Morris A, Holland D, Pappas P, Almela M (2013). HACEK infective endocarditis: characteristics and outcomes from a large, multi-national cohort. PLoS One.

[71] Naushad S, Adeolu M, Goel N, Khadka B, Al-Dahwi A, Gupta RS (2015). Phylogenomic and molecular demarcation of the core members of the polyphyletic pasteurellaceae genera actinobacillus, haemophilus, and pasteurella. Int J Genomics.

[72] Sato T, Watanabe K, Kumada H, Toyama T, Tani-Ishii N, Hamada N (2012). Peptidoglycan of Actinomyces naeslundii induces inflammatory cytokine production and stimulates osteoclastogenesis in alveolar bone resorption. Arch Oral Biol.

[73] Hall V (2008). Actinomyces—gathering evidence of human colonization and infection. Anaerobe.

[74] Eribe ERK, Olsen I (2008). Leptotrichia species in human infections. Anaerobe.

[75] Lloyd-Price J, Arze C, Ananthakrishnan AN, Schirmer M, Avila-Pacheco J, Poon TW, et al. Multi-omics of the gut microbial ecosystem in inflammatory bowel diseases. Nature. 2019;569(7758):655–62. 10.1038/s41586-019-1237-9 PMC6650278 31142855

[76] Chiu C-H, Wang Y-T, Walther BA, Chao A (2014). An improved nonparametric lower bound of species richness via a modified good–turing frequency formula. Biometrics.

[77] Zeus M, Janssen S, Laws H-J, Fischer U, Borkhardt A, Oommen PT. Results from a pilot study on the oral microbiome in children and adolescents with chronic nonbacterial osteomyelitis. Z Rheumatol. 2021:1–10. 10.1007/s00393-021-01035-x PMC9981501 34196794

[78] Zhang X, Zhang D, Jia H, Feng Q, Wang D, Liang D (2015). The oral and gut microbiomes are perturbed in rheumatoid arthritis and partly normalized after treatment. Nat Med.

[79] Kabeerdoss J, Sankaran V, Pugazhendhi S, Ramakrishna B (2013). Clostridium leptum group bacteria abundance and diversity in the fecal microbiota of patients with inflammatory bowel disease: a case-control study in India. BMC Gastroenterol.

[80] Atarashi K, Tanoue T, Shima T, Imaoka A, Kuwahara T, Momose Y (2011). Induction of colonic regulatory T cells by indigenous clostridium species. Science.

[81] Song H, Yoo Y, Hwang J, Na Y-C, Kim HS (2016). Faecalibacterium prausnitzii subspecies–level dysbiosis in the human gut microbiome underlying atopic dermatitis. J Allergy Clin Immunol.

[82] van Dijkhuizen EHP, Del Chierico F, Malattia C, Russo A, Pires Marafon D, ter Haar NM (2019). Microbiome analytics of the gut microbiota in patients with juvenile idiopathic arthritis: a longitudinal observational cohort study. Arthritis Rheum.

[83] Perry A, Lambert P (2011). Propionibacterium acnes: infection beyond the skin. Expert Rev Anti-Infect Ther.

[84] Shapira L, Soskolne WA, Sela MN, Offenbacher S, Barak V (1994). The secretion of PGE2, IL-1β, IL-6, and TNFα by adherent mononuclear cells from early onset periodontitis patients. J Periodontol.

[85] Girschick HJ, Huppertz H-I, Harmsen D, Krauspe R, Müller-Hermelink HK, Papadopoulos T (1999). Chronic recurrent multifocal osteomyelitis in children: diagnostic value of histopathology and microbial testing. Hum Pathol.

[86] Janda WM (2013). Update on the HACEK group of fastidious gram-negative bacilli. Part I. Clin Microbiol Newsl.

[87] Janda WM (2013). Update on the HACEK group of fastidious gram-negative bacilli. Part II. Clin Microbiol Newslet.

[88] Cassel SL, Janczy JR, Bing X, Wilson SP, Olivier AK, Otero JE (2014). Inflammasome-independent IL-1β mediates autoinflammatory disease in Pstpip2-deficient mice. Proc Natl Acad Sci.

[89] Bui FQ, Johnson L, Roberts J, Hung SC, Lee J, Atanasova KR (2016). Fusobacterium nucleatum infection of gingival epithelial cells leads to NLRP3 inflammasome-dependent secretion of IL-1β and the danger signals ASC and HMGB1. Cell Microbiol.

[90] Åberg CH, Kelk P, Johansson A (2015). Aggregatibacter actinomycetemcomitans: virulence of its leukotoxin and association with aggressive periodontitis. Virulence.

[91] Yilmaz Ö, Lee KL (2015). The inflammasome and danger molecule signaling: at the crossroads of inflammation and pathogen persistence in the oral cavity. Periodontol.

[92] Kuo T-R, Chen C-H (2017). Bone biomarker for the clinical assessment of osteoporosis: recent developments and future perspectives. Biomark Res.

[93] Accetto T, Avgustin G (2015). Polysaccharide utilization locus and CAZYme genome repertoires reveal diverse ecological adaptation of Prevotella species. Syst Appl Microbiol.

[94] Gupta VK, Chaudhari NM, Iskepalli S, Dutta C (2015). Divergences in gene repertoire among the reference Prevotella genomes derived from distinct body sites of human. BMC Genomics.

[95] Scher JU, Sczesnak A, Longman RS, Segata N, Ubeda C, Bielski C (2013). Expansion of intestinal Prevotella copri correlates with enhanced susceptibility to arthritis. eLife.

[96] Konig MF, Abusleme L, Reinholdt J, Palmer RJ, Teles RP, Sampson K (2016). Aggregatibacter actinomycetemcomitans–induced hypercitrullination links periodontal infection to autoimmunity in rheumatoid arthritis. Sci Transl Med.

[97] Acharya A, Chan Y, Kheur S, Kheur M, Gopalakrishnan D, Watt RM (2017). Salivary microbiome of an urban Indian cohort and patterns linked to subclinical inflammation. Oral Dis.

[98] Ohmori Y, Honda K, Kikuchu H, Hanazawa S, Amano S, Hirose K (1988). Inducing effect of periodontopathic bacteria on interleukin-1 production by mouse peritoneal macrophages. Oral Microbiol Immunol.

[99] Kistowska M, Gehrke S, Jankovic D, Kerl K, Fettelschoss A, Feldmeyer L (2014). IL-1beta drives inflammatory responses to propionibacterium acnes in vitro and in vivo. J Invest Dermatol.

[100] Colina M, Lo Monaco A, Khodeir M, Trotta F (2007). Propionibacterium acnes and SAPHO syndrome: a case report and literature review. Clin Exp Rheumatol.

[101] Wong JSJ, Seaward LM, Ho CP, Anderson TP, Lau EOC, Amodeo MR (2010). Corynebacterium accolens-associated pelvic osteomyelitis. J Clin Microbiol.

[102] Brook I, Frazier EH (1991). Infections caused by Propionibacterium species. Rev Infect Dis.

[103] Di Ciaula A, Stella A, Bonfrate L, Wang DQH, Portincasa P (2020). Gut microbiota between environment and genetic background in familial Mediterranean fever (FMF). Genes.

[104] Deshayes S, Fellahi S, Bastard JP, Launay JM, Callebert J, Fraisse T (2019). Specific changes in faecal microbiota are associated with familial Mediterranean fever. Ann Rheum Dis.

[105] Dong YQ, Wang W, Li J, Ma MS, Zhong LQ, Wei QJ (2019). Characterization of microbiota in systemic-onset juvenile idiopathic arthritis with different disease severities. World J Clin Cases.

