# Supplementary material for: Analysis of the fecal and oral microbiota in chronic recurrent multifocal osteomyelitis
Source: Arthritis Res Ther. 2022 Feb 22;24:54. doi: 10.1186/s13075-021-02711-8 (PMC8862485; doi:10.1186/s13075-021-02711-8)
Supplement: Supplementary file 1 — Additional file 1:. Supplemental methods. Supplemental Figures S1–S5. Supplemental Tables S1–S9. [file 13075_2021_2711_MOESM1_ESM.pdf]

Supplement: Analysis of the fecal and oral microbiota in chronic recurrent multifocal osteomyelitis

Philipp Rausch[1,2], Meike Hartmann[3], John F. Baines[4,5,*], Philipp von Bismarck[3,*]

[1] Institute for Clinical Molecular Biology (IKMB), Christian-Albrechts-University of Kiel, Kiel, Germany

[2] Laboratory of Genomics and Molecular Biomedicine, Department of Biology, University of Copenhagen, Denmark

[3] Clinic for general pediatrics, Christian-Albrechts-University of Kiel, Kiel, Germany

[4] Max Planck Institute for Evolutionary Biology, Plön, Germany

[5] Institute for Experimental Medicine, Christian-Albrechts-University of Kiel, Kiel, Germany

* Corresponding authors: John F. Baines (baines@evolbio.mpg.de), Philipp von Bismarck (philipp.vonbismarck@uksh.de)

Supplemental methods:

DNA extraction and 16S rRNA gene sequencing: DNA was extracted from saliva and stool samples samples (stored at -20°C in stabilizing solution) using the PSP Saliva Gene DNA Kit and PSP Spin Stool DNA Kit respectively following the manufacturer's protocol (STRATEC Molecular GmbH, Germany). The 16S rRNA gene was amplified using uniquely barcoded primers flanking the V1 and V2 hypervariable regions (27F-338R) with fused MiSeq adapters in a 25 µl PCR. We used 4 µl of each forward and reverse primer (0.28 µM), 0.5 µl dNTPs (200 µM each), 0.25 µl Phusion Hot Start II High-Fidelity DNA Polymerase (0.5 Us), 5 µl of HF buffer (Thermo Fisher Scientific, Inc., Waltham, MA, USA) and 1 µl of undiluted DNA or cDNA. PCRs were conducted with the following cycling conditions (98°C-30s, 30×[98°C-9s, 55°C-60s, 72°C-90s], 72°C-10 min) and checked on a 1.5 % agarose gel. The concentration of the amplicons was estimated using a Gel Doc™ XR+ System coupled with Image Lab™ Software (BioRad, Hercules, CA USA) with 3 µl of O'GeneRulerTM 100 bp Plus DNA Ladder (Thermo Fisher Scientific, Inc., Waltham, MA, USA) as the internal standard for band intensity measurement. The samples of individual gels were pooled into approximately equimolar sub pools as indicated by band intensity and measured with the Qubit dsDNA br Assay Kit (Life Technologies GmbH, Darmstadt, Germany). Sub pools were mixed in an equimolar fashion and stored at -20°C until sequencing. Sequencing was performed on the Illumina MiSeq platform with v3 chemistry.

Sequence processing and quality control: Raw BCL files were demultiplexed and transformed to FASTQ via *bcl2fastq v2.18.0.12* using both forward and reverse barcodes allowing no mismatch in the barcode region. Sequence pairs were filtered and merged via *USEARCH v8.0.1623* (quality threshold for truncation: 10, minimum length single: 200; minimum length after merge: 300; maximum length after merge: 350; minimum overlap: 100; maximum differences in overlap: 1) [1]. Quality filtering was done using the *FASTX tool v0.0.13* with a minimum quality score of 30/33 in at least 99% of the sequence [2]. A second round of quality filtering was performed via *USEARCH* (-fastq_maxee 0.1). Chimeric sequences were determined using *USEARCH* (database informed *UCHIME* algorithm and

the rdp core and gold databases) [1, 3]. Sequences were classified and confirmed as bacterial using the RDP classifier with ≥80% bootstrap threshold (1000 iterations) using the RDP16 database version as provided by P. Schloss in *mothur* 1.39.5 [4, 5]. For all downstream analyses of diversity and habitat association, we took a random subset of 13641 sequences per sample to normalize the read distribution. OTU binning was performed by using the *mothur opticlust* algorithm with single sequences as starting points for clustering and optimizing following the Matthews correlation coefficient [6].

Supplemental figures:

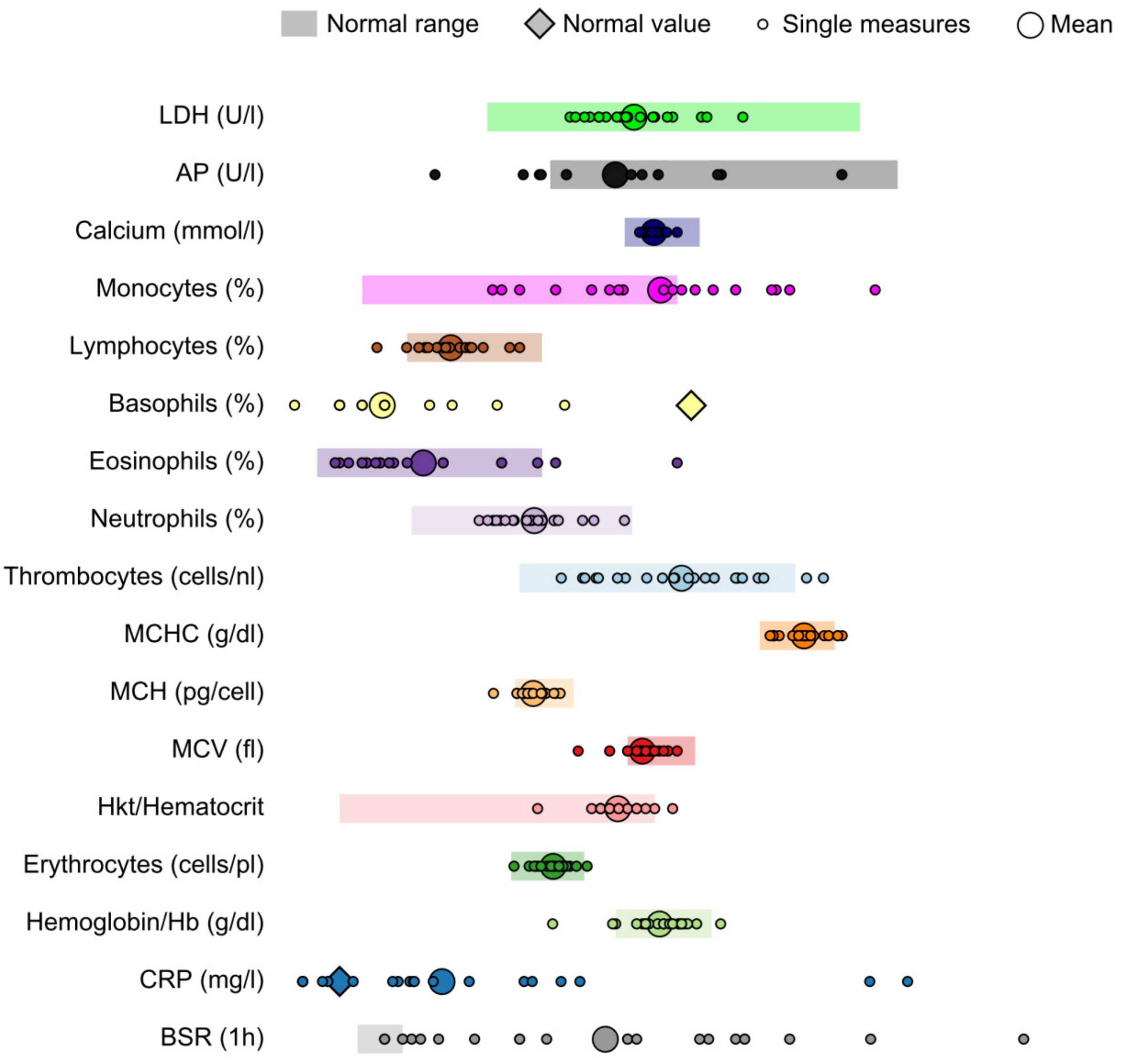

Figure S1: Single data points display scaled values of the respective clinical measurements, while large symbols marking the mean. The transparent bars or diamond symbols show the normal reference ranges/values of the respective clinical parameters (see Table 1). Normal physiological ranges/values of the respective clinical measures are based on the UKSH Clinical Chemistry Department or Oster 2015 [7].

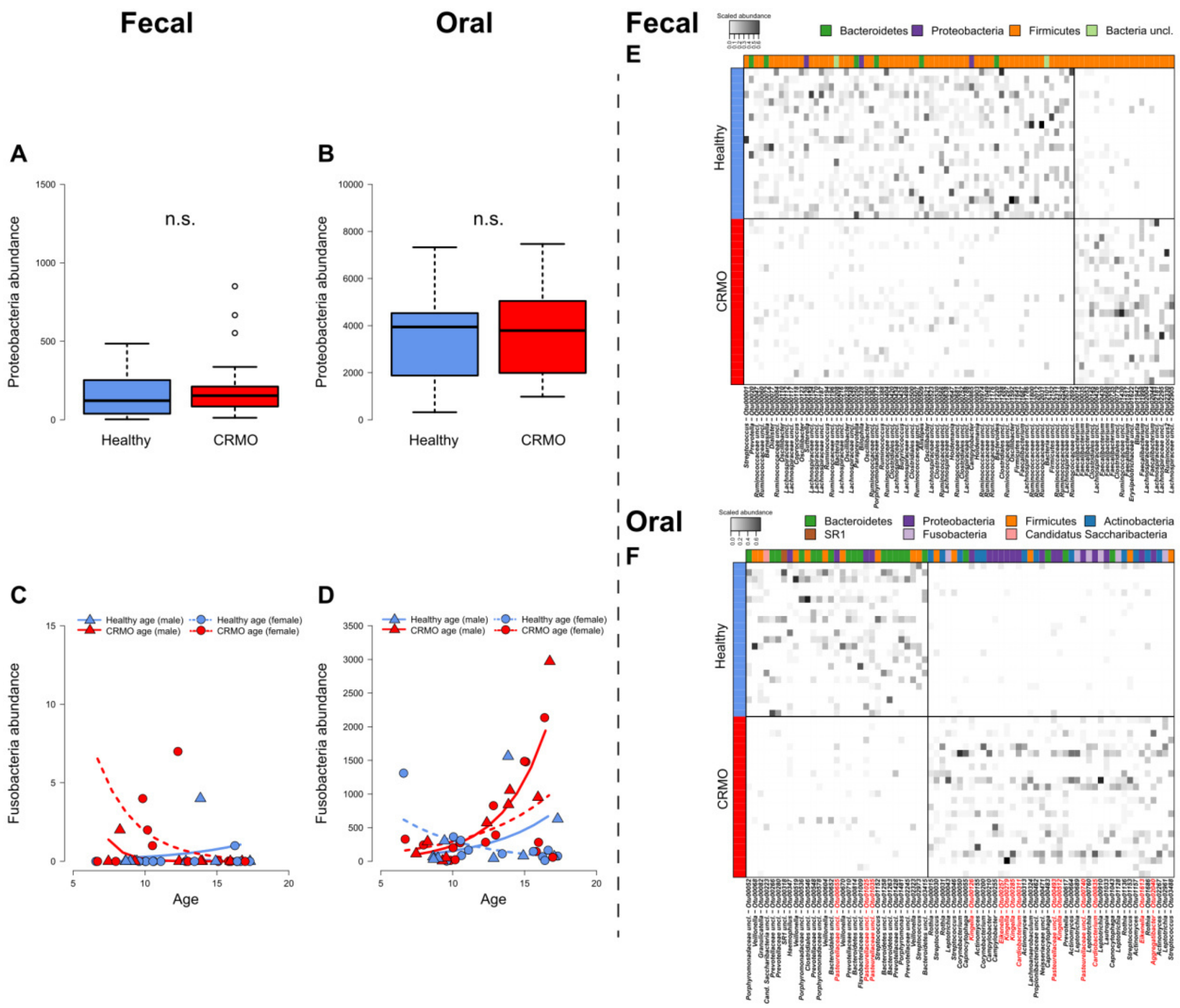

Figure S2: Abundance differences of low abundant phyla in the fecal (**A**, **C**) and oral microbiome (**B**, **D**). Shown are the results according to the best statistical model (see Table 2; n.s. P>0.05). Heatmaps show the distribution of indicator OTUs (min. >1 occurrence) significantly associated to CRMO patients or healthy control individuals based on indicator species analysis [8, 9] of fecal (**E**) and oral (**F**) microbial communities (Table S1). Red taxon names highlight potential HACEK members.

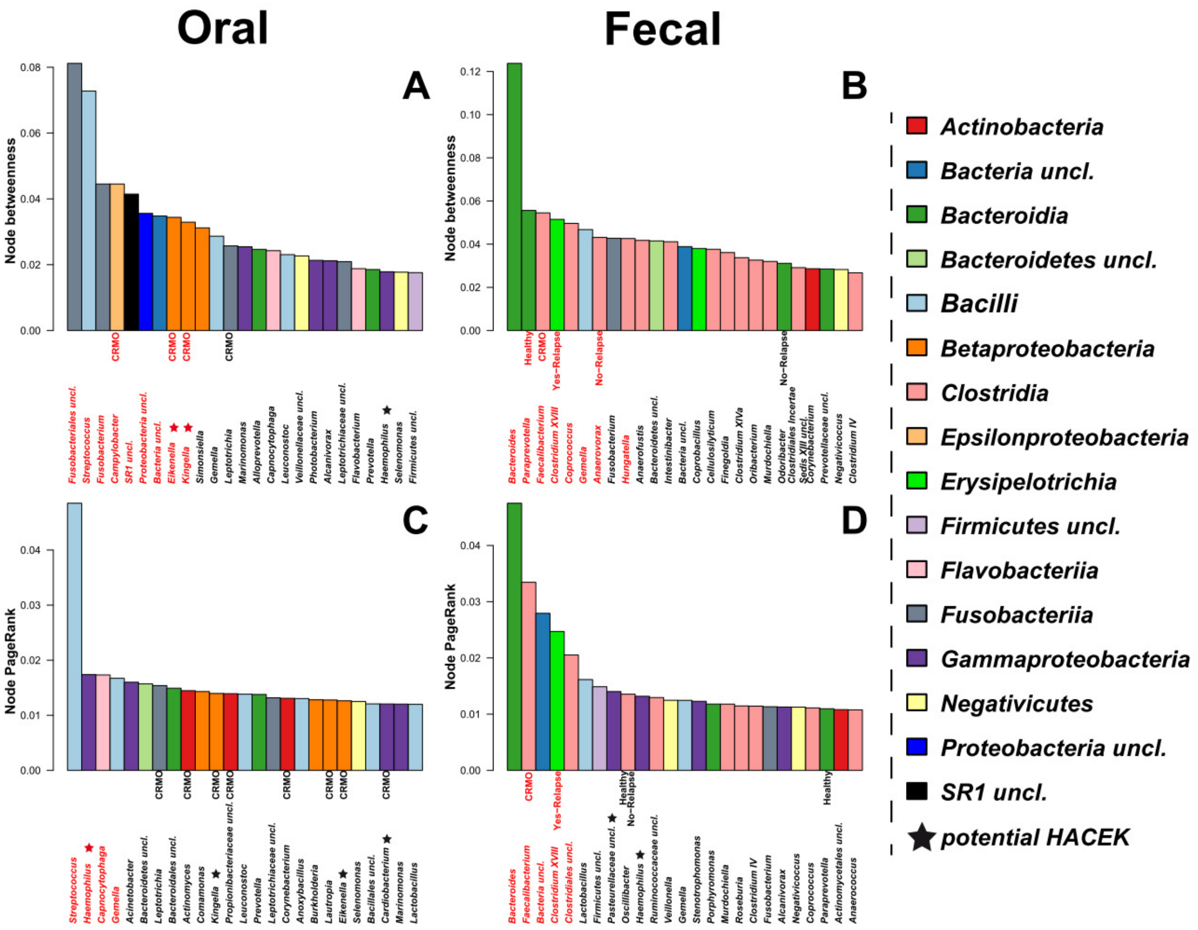

Figure S3: Barplots show the top 30 most central bacteria in oral (**A**, **C**) and fecal (**B**, **D**) bacterial consensus genus networks (node betweenness: **A, B**; PageRank: **C**, **D**; see Table S3). Bar colors indicate bacterial classification at the class level. Names highlighted in red indicate higher than average centrality values according to network permutation tests. Additionally, significant indicator species associations are added above the names and potential HACEK membership (★) is indicated next to genus names.

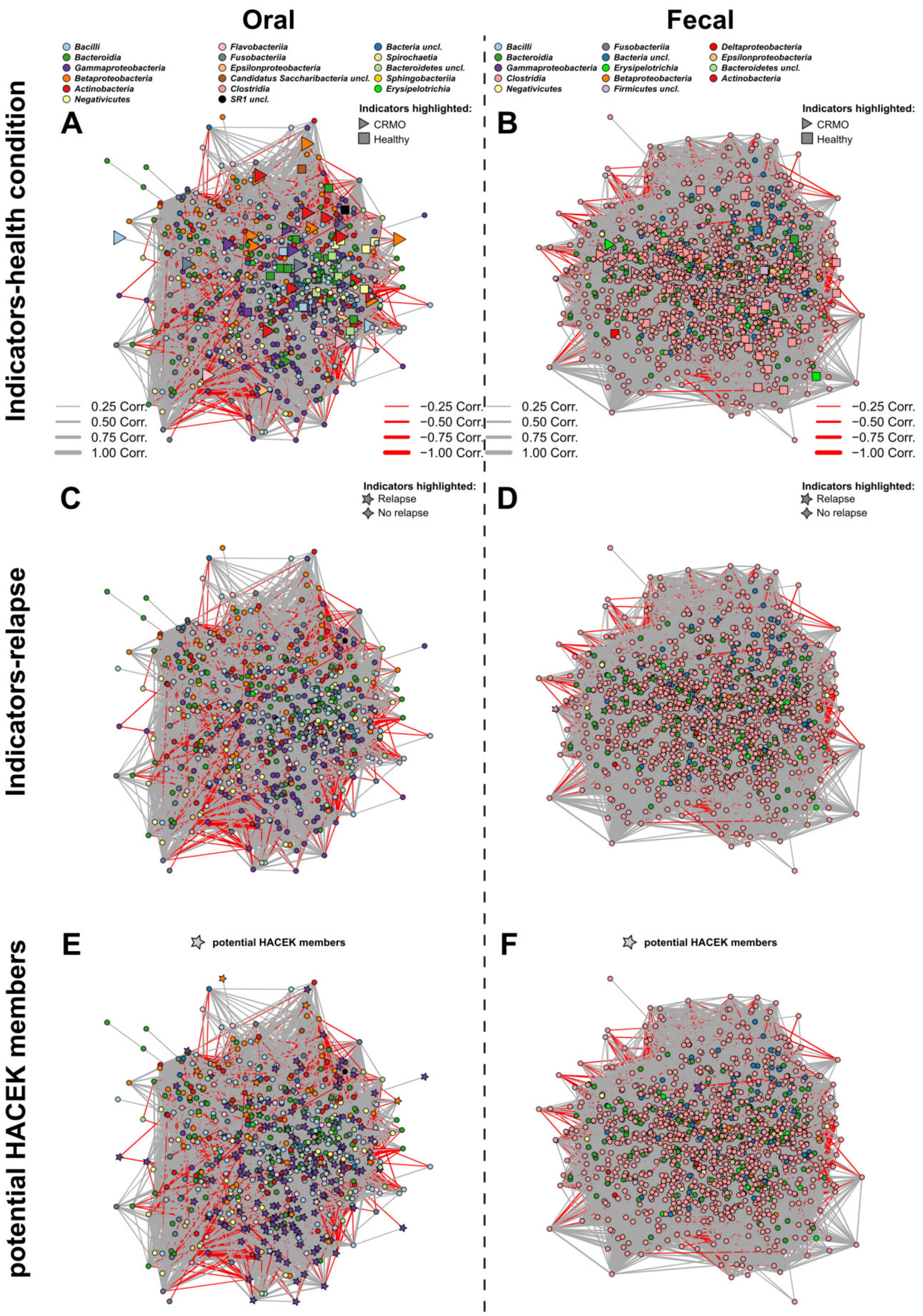
Oral
Fecal
Indicators-health condition
Indicators-relapse
potential HACEK members
Bacilli
Bacteroidia
Gammaproteobacteria
Betaproteobacteria
Actinobacteria
Negativicutes
Flavobacteriia
Fusobacteriia
Epsilonproteobacteria
Candidatus Saccharibacteria uncl.
Clostridia
SR1 uncl.
Bacteria uncl.
Spirochaetia
Bacteroidetes uncl.
Sphingobacteriia
Erysipelotrichia
Deltaproteobacteria
Firmicutes uncl.
A
B
C
D
E
F
Indicators highlighted:
CRMO
Healthy
Relapse
No relapse
potential HACEK members
0.25 Corr.
0.50 Corr.
0.75 Corr.
1.00 Corr.
−0.25 Corr.
−0.50 Corr.
−0.75 Corr.
−1.00 Corr.

Figure S4: Co-abundance networks of oral (**A**, **C**, **E**) and fecal OTUs (**B**, **D**, **F**) based on SparCC correlation [10]. Vertex colors show class level taxonomy and edge width and color shows the strength and direction of bacterial correlations. Vertex shapes highlight indicator species [8, 9] for healthy or CRMO associated OTUs (■-healthy, ▲-CRMO; **A** & **B**), or bacteria associated to disease relapse (★-Relapse,✦-No relapse: **C** & **D**). Networks **E** and **F** show the positions of OTUs potentially belonging to the HACEK group in the oral and fecal co-abundance networks (★-HACEK).

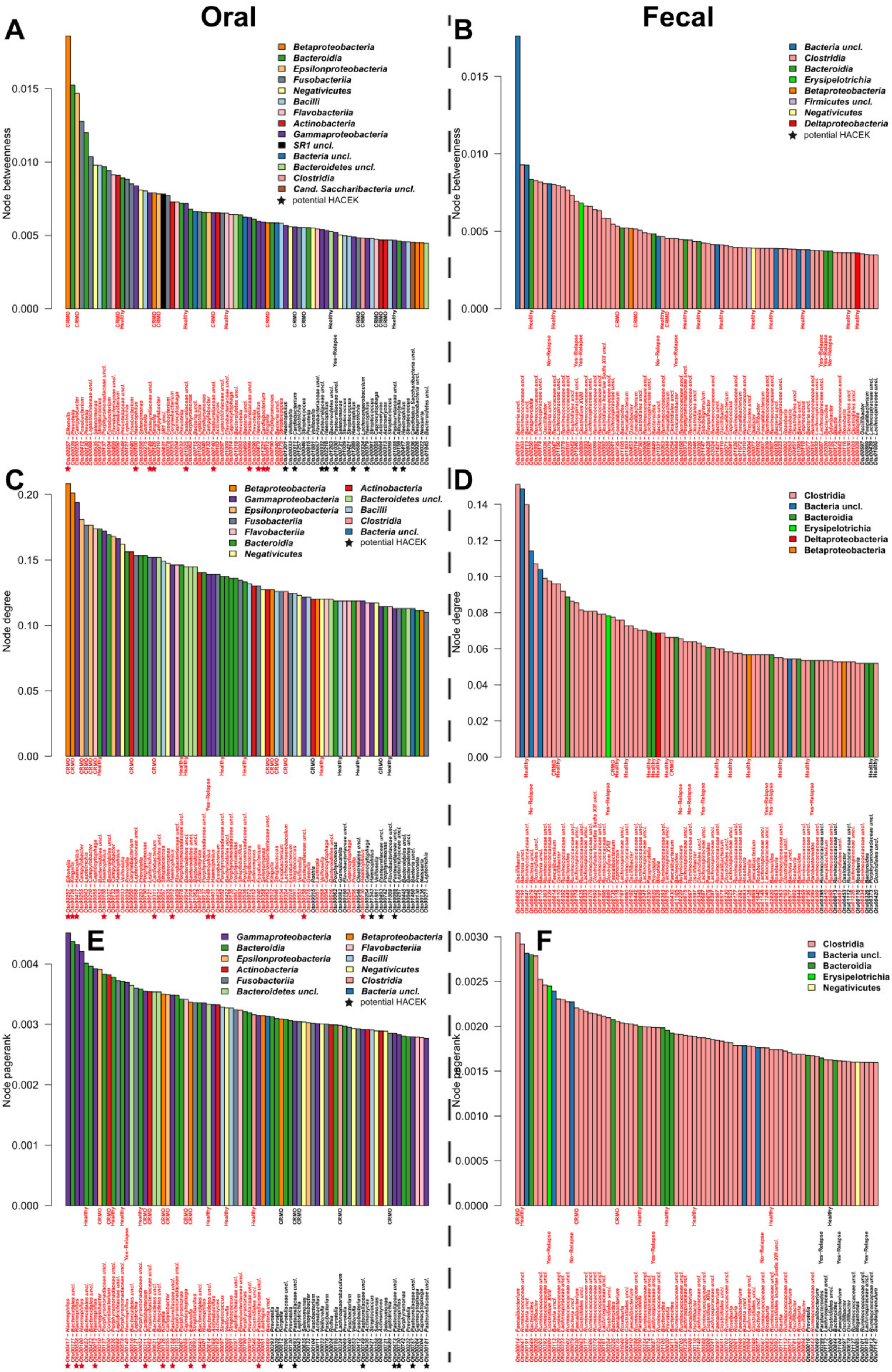
Oral
Fecal
A
B
C
D
E
F
Node betweenness
Node degree
Node pagerank
Betaproteobacteria
Bacteroidia
Epsilonproteobacteria
Fusobacteriia
Negativicutes
Bacilli
Flavobacteriia
Actinobacteria
Gammaproteobacteria
SR1 uncl.
Bacteria uncl.
Bacteroidetes uncl.
Clostridia
Cand. Saccharibacteria uncl.
potential HACEK
Erysipelotrichia
Firmicutes uncl.
Deltaproteobacteria
CRMO
Healthy
Yes–Relapse
No–Relapse

Figure S5: Barplots show the top 80 most central bacteria in oral (**A**, **C**, **E**) and fecal (**B**, **D**, **F**) bacterial OTU networks (betweenness: **A**, **B**; node degree: **C**, **D**; PageRank: **E**, **F**; see Table S4). Bar colors indicate bacterial classification at the class level. Names highlighted in red indicate higher than average centrality values according to network permutation tests (Table S4). Additionally, significant indicator species associations are added above the names and potential HACEK membership (★) is indicated next to genus names.

Supplemental tables:

Table S1: Indicator species level OTUs associated to healthy and CRMO fecal and oral communities (potential HACEK-group members are highlighted in **bold** face).

Table S2: Top twenty five most important bacterial genera and species level OTUs of fecal and oral microbial communities separating healthy and CRMO individuals using supervised classification via RandomForest (potential HACEK-group members are highlighted in **bold** face).

Table S3: Significant taxon centralities in the consensus genus networks of fecal and oral communities based on network permutation (10’000 permutations, one-sided *Z* test). Potential members of the HACEK-group members are highlighted in **bold** face and taxa in red mark repeated detections.

Table S4: Significant taxon centralities in the species level OTU networks of fecal and oral communities based on network permutation (10’000 permutations, one-sided *Z* test). Potential members of the HACEK-group members are highlighted in **bold** face and taxa in red mark repeated detections.

Table S5: Consensus genera associated to clinical measurements CRMO fecal and oral communities via Euclidean distance correlation and Spearman rank correlation (SCC-Spearman correlation coefficient). Potential HACEK-group members are highlighted in **bold** face and red highlights taxa with multiple associations. The *P*-value cutoff was set to $P \leq 0.010$.

Table S6: Species level OTUs from CRMO fecal communities associated to clinical measurements via Euclidean distance correlation and Spearman rank correlation (SCC-Spearman correlation coefficient). Indicators for healthy individuals are marked with +; # indicators for CRMO; ‡ indicators for relapse; † indicators for no relapse. HACEK-group members are highlighted in **bold** face and red indicates OTUs with multiple associations. The *P*-value cutoff was set to $P \leq 0.010$.

Table S7: Species level OTUs from CRMO oral communities associated to clinical measurements via Euclidean distance correlation and Spearman rank correlation (SCC-Spearman correlation coefficient). Indicators for healthy individuals are marked with +; # indicators for CRMO; ‡ indicators for relapse; † indicators for no relapse. HACEK-group members are highlighted in **bold** face and red indicates OTUs with multiple associations. The *P*-value cutoff was set to $P \leq 0.010$.

Table S8: Association of alpha diversity measures (ACE species richness, Shannon H numbers equivalent) in the fecal and oral microbial communities with the clinical measurements in CRMO patients. Lines highlighted in red show nominally significant Euclidean distance correlations.

Table S9: Association of beta diversity measures of fecal and oral microbial communities with clinical measurements in CRMO patients via PERMANOVA. Red highlights nominally significant associations between beta diversity and clinical measurements.

Supplemental references:

1. Edgar RC: **Search and clustering orders of magnitude faster than BLAST**. *Bioinformatics* 2010, **26**(19):2460-2461.

2. Hannon G: **FASTX-Toolkit**. In. http://hannonlab.cshl.edu/fastx_toolkit; 2010.

3. Edgar RC, Haas BJ, Clemente JC, Quince C, Knight R: **UCHIME improves sensitivity and speed of chimera detection**. *Bioinformatics* 2011, **27**(16):2194-2200.

4. Wang Q, Garrity GM, Tiedje JM, Cole JR: **Naive Bayesian Classifier for Rapid Assignment of rRNA Sequences into the New Bacterial Taxonomy**. *Applied and environmental microbiology* 2007, **73**(16):5261-5267.

5. Schloss PD, Westcott SL, Ryabin T, Hall JR, Hartmann M, Hollister EB, Lesniewski RA, Oakley BB, Parks DH, Robinson CJ *et al*: **Introducing mothur: Open Source, Platform-independent, Community-supported Software for Describing and Comparing Microbial Communities**. *Applied and environmental microbiology* 2009, **75**(23):7537-7541.

6. Westcott SL, Schloss PD: **OptiClust, an Improved Method for Assigning Amplicon-Based Sequence Data to Operational Taxonomic Units**. *mSphere* 2017, **2**(2).

7. Oster O: **Pädiatrisch relevante Referenzwerte: klinische Chemie**. In: *Pädiatrie: Grundlagen und Praxis.* Edited by Hoffmann GF, Lentze MJ, Spranger J, Zepp F. Berlin, Heidelberg: Springer Berlin Heidelberg; 2015: 1-67.

8. De Cáceres M, Legendre P, Moretti M: **Improving indicator species analysis by combining groups of sites**. *Oikos* 2010, **119**(10):1674-1684.

9. De Cáceres M, Legendre P, Wiser SK, Brotons L: **Using species combinations in indicator value analyses**. *Methods in Ecology and Evolution* 2012:n/a-n/a.

10. Friedman J, Alm EJ: **Inferring Correlation Networks from Genomic Survey Data**. *PLoS Computational Biology* 2012, **8**(9):e1002687.

Table S1: Indicator species level OTUs associated to healthy and CRMO fecal and oral communities (potential HACEK-group members are highlighted in **bold** face).

Data	Factor	Assoc.	OTU-ID	*IndVal.g*	*P*-Value	P_{FDR}	Phylum (RDP16)	Genus (RDP16)
Fecal	Disease	CRMO	OTU-2905	0.47673	0.04770	0.70522	Firmicutes	*Lachnospiraceae uncl.*
			OTU-2330	0.47673	0.04780	0.70522	Firmicutes	*Ruminococcus2*
			OTU-2295	0.47673	0.04940	0.70522	Firmicutes	*Lachnospiraceae uncl.*
			OTU-2181	0.47673	0.04980	0.70522	Firmicutes	*Lachnospiraceae uncl.*
			OTU-2044	0.53247	0.04350	0.70522	Firmicutes	*Faecalibacterium*
			OTU-2004	0.56408	0.00920	0.47221	Firmicutes	*Lachnospiraceae uncl.*
			OTU-1998	0.52438	0.04480	0.70522	Firmicutes	*Faecalibacterium*
			OTU-1942	0.53540	0.04170	0.70522	Firmicutes	*Blautia*
			OTU-1622	0.51854	0.04250	0.70522	Firmicutes	*Erysipelotrichaceae uncl.*
			OTU-1517	0.54447	0.02100	0.61333	Firmicutes	*Faecalibacterium*
			OTU-1430	0.47673	0.04910	0.70522	Firmicutes	*Ruminococcaceae uncl.*
			OTU-779	0.63175	0.00410	0.47221	Firmicutes	*Clostridiales uncl.*
			OTU-735	0.75194	0.00600	0.47221	Firmicutes	*Faecalibacterium*
			OTU-668	0.73713	0.02150	0.61367	Firmicutes	*Faecalibacterium*
			OTU-430	0.80670	0.00510	0.47221	Firmicutes	*Faecalibacterium*
			OTU-426	0.72125	0.00830	0.47221	Firmicutes	*Lachnospiraceae uncl.*
			OTU-145	0.61309	0.03280	0.70522	Firmicutes	*Clostridiales uncl.*
			OTU-53	0.79759	0.03690	0.70522	Firmicutes	*Faecalibacterium*
			OTU-35	0.87268	0.00750	0.47221	Firmicutes	*Faecalibacterium*
			OTU-14	0.81137	0.01410	0.59026	Firmicutes	*Faecalibacterium*
		Healthy	OTU-2101	0.51903	0.02900	0.70039	Bacteria uncl.	*Bacteria uncl.*
			OTU-208	0.75319	0.04440	0.70522	Bacteria uncl.	*Bacteria uncl.*
			OTU-1220	0.52440	0.03120	0.70522	Bacteroidetes	*Bacteroides*
			OTU-509	0.49800	0.03120	0.70522	Bacteroidetes	*Alistipes*
			OTU-373	0.58073	0.04930	0.70522	Bacteroidetes	*Porphyromonadaceae uncl.*
			OTU-250	0.56003	0.03610	0.70522	Bacteroidetes	*Paraprevotella*
			OTU-72	0.71150	0.02900	0.70039	Bacteroidetes	*Barnesiella*
			OTU-20	0.70410	0.00940	0.47221	Bacteroidetes	*Prevotella*
			OTU-2692	0.50000	0.01810	0.59252	Firmicutes	*Ruminococcaceae uncl.*
			OTU-2631	0.54772	0.00640	0.47221	Firmicutes	*Lachnospiraceae uncl.*
			OTU-2528	0.51042	0.04100	0.70522	Firmicutes	*Ruminococcaceae uncl.*
			OTU-2311	0.54772	0.00800	0.47221	Firmicutes	*Ruminococcaceae uncl.*

		OTU-2152	0.50000	0.01840	0.59252	Firmicutes	*Firmicutes uncl.*
		OTU-2031	0.50000	0.01790	0.59252	Firmicutes	*Ruminococcaceae uncl.*
		OTU-2012	0.52199	0.03790	0.70522	Firmicutes	*Ruminococcaceae uncl.*
		OTU-1800	0.50000	0.01750	0.59252	Firmicutes	*Ruminococcaceae uncl.*
		OTU-1786	0.54420	0.04040	0.70522	Firmicutes	*Lachnospiraceae uncl.*
		OTU-1687	0.58589	0.02400	0.66343	Firmicutes	*Faecalibacterium*
		OTU-1641	0.54772	0.00740	0.47221	Firmicutes	*Firmicutes uncl.*
		OTU-1592	0.50000	0.01750	0.59252	Firmicutes	*Oscillibacter*
		OTU-1297	0.63246	0.00130	0.43956	Firmicutes	*Ruminococcaceae uncl.*
		OTU-1288	0.50000	0.01750	0.59252	Firmicutes	*Clostridiales uncl.*
		OTU-1176	0.64495	0.01150	0.53491	Firmicutes	*Ruminococcaceae uncl.*
		OTU-1049	0.57792	0.04390	0.70522	Firmicutes	*Ruminococcaceae uncl.*
		OTU-974	0.68361	0.00090	0.43956	Firmicutes	*Ruminococcaceae uncl.*
		OTU-903	0.61266	0.01930	0.59731	Firmicutes	*Holdemania*
		OTU-788	0.70628	0.00710	0.47221	Firmicutes	*Lachnospiraceae uncl.*
		OTU-752	0.66675	0.03610	0.70522	Firmicutes	*Clostridiales uncl.*
		OTU-681	0.76222	0.00140	0.43956	Firmicutes	*Ruminococcaceae uncl.*
		OTU-647	0.64254	0.04610	0.70522	Firmicutes	*Holdemania*
		OTU-638	0.52309	0.02530	0.67242	Firmicutes	*Lachnospiraceae uncl.*
		OTU-566	0.69052	0.00650	0.47221	Firmicutes	*Ruminococcaceae uncl.*
		OTU-530	0.70808	0.03190	0.70522	Firmicutes	*Clostridiales uncl.*
		OTU-523	0.71331	0.01390	0.59026	Firmicutes	*Lachnospiraceae uncl.*
		OTU-521	0.52660	0.04220	0.70522	Firmicutes	*Oscillibacter*
		OTU-507	0.71430	0.00910	0.47221	Firmicutes	*Ruminococcaceae uncl.*
		OTU-500	0.68821	0.04470	0.70522	Firmicutes	*Clostridiales uncl.*
		OTU-498	0.66879	0.02030	0.60701	Firmicutes	*Lachnospiraceae uncl.*
		OTU-435	0.75484	0.04170	0.70522	Firmicutes	*Butyricicoccus*
		OTU-434	0.69549	0.04110	0.70522	Firmicutes	*Lachnospiraceae uncl.*
		OTU-420	0.77117	0.01070	0.51684	Firmicutes	*Clostridiales uncl.*
		OTU-404	0.77787	0.04750	0.70522	Firmicutes	*Ruminococcaceae uncl.*
		OTU-388	0.72439	0.01760	0.59252	Firmicutes	*Ruminococcus*
		OTU-363	0.73030	0.04630	0.70522	Firmicutes	*Ruminococcaceae uncl.*
		OTU-360	0.77405	0.00350	0.47221	Firmicutes	*Oscillibacter*
		OTU-248	0.77527	0.04480	0.70522	Firmicutes	*Lachnospiraceae uncl.*
		OTU-238	0.81911	0.00560	0.47221	Firmicutes	*Oscillibacter*
		OTU-216	0.79316	0.03280	0.70522	Firmicutes	*Lachnospiraceae uncl.*
		OTU-196	0.87250	0.00550	0.47221	Firmicutes	*Ruminococcaceae uncl.*

		OTU-194	0.64124	0.04710	0.70522	Firmicutes	*Ruminococcus*
		OTU-187	0.58992	0.01630	0.59252	Firmicutes	*Lachnospiraceae uncl.*
		OTU-170	0.92267	0.00010	0.12559	Firmicutes	*Lachnospiraceae uncl.*
		OTU-154	0.73470	0.02570	0.67242	Firmicutes	*Lachnospiraceae uncl.*
		OTU-123	0.86042	0.00610	0.47221	Firmicutes	*Oscillibacter*
		OTU-118	0.77091	0.01360	0.59026	Firmicutes	*Coprococcus*
		OTU-117	0.86431	0.01950	0.59731	Firmicutes	*Lachnospiraceae uncl.*
		OTU-112	0.79867	0.02820	0.70039	Firmicutes	*Lachnospiraceae uncl.*
		OTU-110	0.84912	0.00510	0.47221	Firmicutes	*Oscillibacter*
		OTU-84	0.87070	0.00850	0.47221	Firmicutes	*Ruminococcaceae uncl.*
		OTU-77	0.63222	0.00270	0.47221	Firmicutes	*Dialister*
		OTU-60	0.80459	0.00750	0.47221	Firmicutes	*Ruminococcaceae uncl.*
		OTU-47	0.78310	0.04760	0.70522	Firmicutes	*Ruminococcaceae uncl.*
		OTU-1	0.69544	0.02670	0.68432	Firmicutes	*Streptococcus*
		OTU-865	0.55131	0.03880	0.70522	Proteobacteria	*Campylobacter*
		OTU-328	0.76051	0.02430	0.66343	Proteobacteria	*Bilophila*
		OTU-128	0.60103	0.01790	0.59252	Proteobacteria	*Sutterella*
Relapse	No	OTU-1031	0.72166	0.03490	1.00000	Bacteria uncl.	*Bacteria uncl.*
		OTU-79	0.79453	0.02950	1.00000	Bacteria uncl.	*Bacteria uncl.*
		OTU-152	0.87438	0.02520	1.00000	Bacteroidetes	*Odoribacter*
		OTU-2032	0.65465	0.04810	1.00000	Firmicutes	*Faecalibacterium*
		OTU-1373	0.70711	0.04500	1.00000	Firmicutes	*Faecalibacterium*
		OTU-879	0.77033	0.04360	1.00000	Firmicutes	*Anaerovorax*
		OTU-700	0.70711	0.03520	1.00000	Firmicutes	*Oscillibacter*
		OTU-697	0.80178	0.01060	1.00000	Firmicutes	*Ruminococcaceae uncl.*
		OTU-640	0.75272	0.04350	1.00000	Firmicutes	*Lachnospiraceae uncl.*
		OTU-567	0.80178	0.01280	1.00000	Firmicutes	*Ruminococcaceae uncl.*
		OTU-229	0.86593	0.03160	1.00000	Firmicutes	*Butyricicoccus*
	Yes	OTU-2809	0.61237	0.03670	1.00000	Bacteroidetes	*Bacteroides*
		OTU-2615	0.61237	0.03770	1.00000	Bacteroidetes	*Bacteroides*
		OTU-2079	0.66986	0.02920	1.00000	Bacteroidetes	*Bacteroides*
		OTU-2020	0.76015	0.00820	1.00000	Bacteroidetes	*Bacteroides*
		OTU-1419	0.61237	0.03600	1.00000	Bacteroidetes	*Bacteroides*
		OTU-299	0.86267	0.03520	1.00000	Bacteroidetes	*Parabacteroides*
		OTU-141	0.70183	0.04420	1.00000	Bacteroidetes	*Bacteroides*
		OTU-32	0.89409	0.02480	1.00000	Bacteroidetes	*Bacteroides*
		OTU-5647	0.61237	0.04070	1.00000	Firmicutes	*Ruminococcaceae uncl.*

			OTU-4399	0.61237	0.03560	1.00000	Firmicutes	*Lachnospiraceae uncl.*
			OTU-4186	0.61237	0.04020	1.00000	Firmicutes	*Faecalibacterium*
			OTU-3893	0.61237	0.04070	1.00000	Firmicutes	*Lachnospiraceae uncl.*
			OTU-3879	0.61237	0.03580	1.00000	Firmicutes	*Lachnospiraceae uncl.*
			OTU-3586	0.61237	0.03480	1.00000	Firmicutes	*Faecalibacterium*
			OTU-3187	0.61237	0.03630	1.00000	Firmicutes	*Roseburia*
			OTU-3116	0.61237	0.03470	1.00000	Firmicutes	*Lachnospiraceae uncl.*
			OTU-2880	0.61237	0.03300	1.00000	Firmicutes	*Lachnospiraceae uncl.*
			OTU-2811	0.61237	0.03720	1.00000	Firmicutes	*Lachnospiraceae uncl.*
			OTU-2082	0.62361	0.04200	1.00000	Firmicutes	*Lachnospiraceae uncl.*
			OTU-2006	0.61237	0.03460	1.00000	Firmicutes	*Lachnospiraceae uncl.*
			OTU-1903	0.79057	0.00210	1.00000	Firmicutes	*Lachnospiraceae uncl.*
			OTU-1636	0.61237	0.03670	1.00000	Firmicutes	*Ruminococcaceae uncl.*
			OTU-1593	0.61020	0.03650	1.00000	Firmicutes	*Faecalibacterium*
			OTU-1268	0.79057	0.00250	1.00000	Firmicutes	*Firmicutes uncl.*
			OTU-1246	0.75245	0.01460	1.00000	Firmicutes	*Lachnospiraceae uncl.*
			OTU-1104	0.68313	0.02930	1.00000	Firmicutes	*Faecalibacterium*
			OTU-984	0.77460	0.04940	1.00000	Firmicutes	*Fusicatenibacter*
			OTU-809	0.73193	0.02020	1.00000	Firmicutes	*Clostridium XVIII*
			OTU-687	0.78745	0.02970	1.00000	Firmicutes	*Lachnospiraceae uncl.*
			OTU-641	0.60457	0.03660	1.00000	Firmicutes	*Ruminococcaceae uncl.*
			OTU-618	0.79772	0.02410	1.00000	Firmicutes	*Lachnospiraceae uncl.*
			OTU-603	0.76233	0.02430	1.00000	Firmicutes	*Ruminococcus2*
			OTU-185	0.70302	0.02430	1.00000	Firmicutes	*Lachnospiraceae uncl.*
			OTU-7	0.61237	0.03490	1.00000	Firmicutes	*Gemella*
		#	OTU-1	0.67990	0.01510	1.00000	Firmicutes	*Streptococcus*
Oral	Disease	CRMO	OTU-2267	0.45644	0.04870	0.45846	Actinobacteria	*Actinomyces*
			OTU-1686	0.56143	0.02440	0.40565	Actinobacteria	*Rothia*
			OTU-1157	0.63344	0.00530	0.33676	Actinobacteria	*Actinomyces*
			OTU-1136	0.55982	0.04600	0.45846	Actinobacteria	*Rothia*
			OTU-664	0.53597	0.02560	0.40565	Actinobacteria	*Actinomyces*
			OTU-462	0.76347	0.00050	0.17473	Actinobacteria	*Propionibacteriaceae uncl.*
			OTU-313	0.76712	0.00490	0.33676	Actinobacteria	*Actinomyces*
			OTU-200	0.83789	0.03490	0.42056	Actinobacteria	*Corynebacterium*
			OTU-155	0.74820	0.04820	0.45846	Actinobacteria	*Actinomyces*
			OTU-50	0.78985	0.01960	0.40565	Actinobacteria	*Corynebacterium*
			OTU-31	0.90058	0.01620	0.40565	Actinobacteria	*Rothia*

		OTU-15	0.88556	0.00160	0.27957	Actinobacteria	*Rothia*
		OTU-1043	0.56688	0.04280	0.45846	Bacteroidetes	*Capnocytophaga*
		OTU-617	0.59558	0.01960	0.40565	Bacteroidetes	*Prevotella*
		OTU-483	0.56297	0.03460	0.42056	Bacteroidetes	*Capnocytophaga*
		OTU-55	0.91184	0.03310	0.41936	Bacteroidetes	*Capnocytophaga*
		OTU-3488	0.45644	0.04980	0.45846	Firmicutes	*Streptococcus*
		OTU-1153	0.60396	0.02870	0.40565	Firmicutes	*Streptococcus*
		OTU-324	0.70059	0.02930	0.40565	Firmicutes	*Lachnoanaerobaculum*
		OTU-46	0.80764	0.04130	0.45846	Firmicutes	*Streptococcus*
		OTU-30	0.84204	0.02630	0.40565	Firmicutes	*Streptococcus*
		OTU-2961	0.45644	0.04670	0.45846	Fusobacteria	*Leptotrichia*
		OTU-1128	0.48947	0.04990	0.45846	Fusobacteria	*Leptotrichia*
		OTU-910	0.53260	0.02790	0.40565	Fusobacteria	*Leptotrichia*
		OTU-760	0.66506	0.00700	0.37567	Fusobacteria	*Leptotrichia*
		OTU-689	0.53571	0.02530	0.40565	Fusobacteria	*Leptotrichia*
		OTU-43	0.88420	0.03680	0.43594	Fusobacteria	*Leptotrichia*
		**OTU-2040**	**0.45644**	**0.04720**	**0.45846**	**Proteobacteria**	***Aggregatibacter***
		**OTU-1613**	**0.51961**	**0.02130**	**0.40565**	**Proteobacteria**	***Eikenella***
		OTU-1033	0.58503	0.02820	0.40565	Proteobacteria	*Lautropia*
		**OTU-835**	**0.50000**	**0.02420**	**0.40565**	**Proteobacteria**	***Cardiobacterium***
		**OTU-740**	**0.64555**	**0.03340**	**0.41936**	**Proteobacteria**	***Pasteurellaceae uncl.***
		**OTU-512**	**0.84175**	**0.00010**	**0.06989**	**Proteobacteria**	***Kingella***
		OTU-493	0.64635	0.04130	0.45846	**Proteobacteria**	***Pasteurellaceae uncl.***
		OTU-471	0.57286	0.04570	0.45846	Proteobacteria	*Neisseriaceae uncl.*
		**OTU-311**	**0.83345**	**0.00860**	**0.37567**	**Proteobacteria**	***Cardiobacterium***
		**OTU-285**	**0.63626**	**0.01500**	**0.40565**	**Proteobacteria**	***Kingella***
	#	**OTU-279**	**0.65238**	**0.03260**	**0.41936**	**Proteobacteria**	***Kingella***
		**OTU-257**	**0.81796**	**0.00320**	**0.27957**	**Proteobacteria**	***Eikenella***
		OTU-255	0.79320	0.00950	0.39058	Proteobacteria	*Campylobacter*
		OTU-210	0.72039	0.02630	0.40565	Proteobacteria	*Campylobacter*
		**OTU-126**	**0.86338**	**0.00220**	**0.27957**	**Proteobacteria**	***Kingella***
	Healthy	OTU-3415	0.46625	0.02370	0.40565	Bacteroidetes	*Bacteroidetes uncl.*
		OTU-2245	0.46625	0.02330	0.40565	Bacteroidetes	*Prevotellaceae uncl.*
		OTU-1481	0.53486	0.01470	0.40565	Bacteroidetes	*Porphyromonas*
		OTU-1428	0.51828	0.04980	0.45846	Bacteroidetes	*Prevotellaceae uncl.*
		OTU-1263	0.51075	0.01160	0.40565	Bacteroidetes	*Bacteroidetes uncl.*
		OTU-1258	0.46625	0.02470	0.40565	Bacteroidetes	*Bacteroidetes uncl.*

		OTU-1009	0.52536	0.02810	0.40565	Bacteroidetes	*Flavobacteriaceae uncl.*
		OTU-814	0.64764	0.00310	0.27957	Bacteroidetes	*Bacteroidetes uncl.*
		OTU-710	0.65642	0.01900	0.40565	Bacteroidetes	*Prevotellaceae uncl.*
		OTU-652	0.60069	0.04540	0.45846	Bacteroidetes	*Bacteroidales uncl.*
		OTU-578	0.65450	0.01730	0.40565	Bacteroidetes	*Porphyromonadaceae uncl.*
		OTU-548	0.74089	0.00110	0.25627	Bacteroidetes	*Prevotellaceae uncl.*
		OTU-536	0.54723	0.03360	0.41936	Bacteroidetes	*Porphyromonadaceae uncl.*
		OTU-280	0.63817	0.01640	0.40565	Bacteroidetes	*Prevotellaceae uncl.*
		OTU-266	0.66795	0.03280	0.41936	Bacteroidetes	*Prevotellaceae uncl.*
		OTU-52	0.83957	0.00730	0.37567	Bacteroidetes	*Porphyromonadaceae uncl.*
		OTU-223	0.66954	0.02700	0.40565	Cand. Saccharibacteria	*Cand. Saccharibacteria uncl.*
		OTU-2973	0.46625	0.02160	0.40565	Firmicutes	*Streptococcus*
		OTU-2323	0.47902	0.04540	0.45846	Firmicutes	*Veillonella*
		OTU-1152	0.58828	0.01410	0.40565	Firmicutes	*Streptococcus*
		OTU-670	0.60303	0.02960	0.40565	Firmicutes	*Veillonella*
		OTU-604	0.54836	0.01260	0.40565	Firmicutes	*Veillonella*
		OTU-546	0.46625	0.02440	0.40565	Firmicutes	*Clostridiales uncl.*
		OTU-519	0.70638	0.00300	0.27957	Firmicutes	*Veillonella*
		OTU-82	0.85137	0.00530	0.33676	Firmicutes	*Granulicatella*
		OTU-68	0.85076	0.00830	0.37567	Firmicutes	*Veillonella*
		**OTU-1035**	**0.57727**	**0.02070**	**0.40565**	**Proteobacteria**	***Pasteurellaceae uncl.***
		**OTU-1025**	**0.60850**	**0.02800**	**0.40565**	**Proteobacteria**	***Pasteurellaceae uncl.***
		**OTU-655**	**0.72275**	**0.00790**	**0.37567**	**Proteobacteria**	***Pasteurellaceae uncl.***
		**OTU-347**	**0.75925**	**0.02220**	**0.40565**	**Proteobacteria**	***Haemophilus***
		OTU-318	0.58363	0.01590	0.40565	SR1	*SR1 uncl.*
Relapse	No	OTU-1346	0.68313	0.04070	1.00000	Bacteroidetes	*Bacteroidetes uncl.*
		OTU-996	0.70411	0.04990	1.00000	**Proteobacteria**	***Pasteurellaceae uncl.***
	#	**OTU-279**	**0.85635**	**0.00790**	**1.00000**	**Proteobacteria**	***Kingella***
	Yes	OTU-3986	0.57735	0.04070	1.00000	Actinobacteria	*Actinomyces*
		OTU-4146	0.57735	0.03920	1.00000	Bacteroidetes	*Porphyromonadaceae uncl.*
		OTU-2814	0.57735	0.04280	1.00000	Bacteroidetes	*Prevotellaceae uncl.*
		OTU-2179	0.57735	0.03920	1.00000	Bacteroidetes	*Capnocytophaga*
		OTU-1071	0.67420	0.04140	1.00000	Bacteroidetes	*Prevotella*
		OTU-5082	0.57735	0.03780	1.00000	Firmicutes	*Streptococcus*
		OTU-3989	0.57735	0.04170	1.00000	Firmicutes	*Streptococcus*
		OTU-2714	0.66667	0.01270	1.00000	Firmicutes	*Streptococcus*
		OTU-2628	0.63564	0.02990	1.00000	Firmicutes	*Streptococcus*

		OTU-1607	0.57735	0.04430	1.00000	Firmicutes	*Streptococcus*
		**OTU-2344**	**0.63564**	**0.03000**	**1.00000**	**Proteobacteria**	***Pasteurellaceae uncl.***
		**OTU-2318**	**0.57735**	**0.04120**	**1.00000**	**Proteobacteria**	***Pasteurellaceae uncl.***
		**OTU-1853**	**0.57735**	**0.03780**	**1.00000**	**Proteobacteria**	***Aggregatibacter***
		**OTU-1030**	**0.63717**	**0.03060**	**1.00000**	**Proteobacteria**	***Pasteurellaceae uncl.***
		**OTU-149**	**0.88434**	**0.02500**	**1.00000**	**Proteobacteria**	***Haemophilus***

Table S2: Top twenty five most important bacterial genera and species level OTUs of fecal and oral microbial communities separating healthy and CRMO individuals using supervised classification via RandomForest (potential HACEK-group members are highlighted in **bold** face).

Data	OTU-ID	Classification	Indicator association	Gini importance
Oral	-	*Corynebacterium*	CRMO	0.38480
	-	*Leptotrichia*	CRMO	0.38219
	-	*Bacteroidales uncl.*		0.37886
	-	***Kingella***	CRMO	0.36425
	-	*Prevotella*		0.34125
	-	*Capnocytophaga*		0.33386
	-	*Actinomyces*	CRMO	0.28600
	-	*Campylobacter*	CRMO	0.26798
	-	***Eikenella***	CRMO	0.26667
	-	*Propionibacteriaceae uncl.*	CRMO	0.24988
	-	*Prevotellaceae uncl.*		0.23891
	-	*Rothia*		0.23160
	-	***Cardiobacterium***	CRMO	0.22993
	-	*Fusobacterium*		0.22473
	-	*Alloprevotella*		0.21320
	-	*Leptotrichiaceae uncl.*		0.21036
	-	*Bacteroides*		0.21025
	-	*Oribacterium*		0.20826
	-	*Neisseria*		0.20059
	-	*Veillonella*	Healthy	0.19530
	-	***Aggregatibacter***		0.19048
	-	*Streptococcus*		0.18800
	-	*Fusobacteriales uncl.*		0.18666
	-	*Bacteria uncl.*		0.18005
	-	*Lautropia*	CRMO	0.17536
Fecal	-	*Oscillibacter*	Healthy; No-Relapse	1.23055
	-	*Barnesiella*	Healthy; No-Relapse	0.98788
	-	*Faecalibacterium*	CRMO	0.90745
	-	*Ruminococcaceae uncl.*		0.85466
	-	*Enterobacteriaceae uncl.*		0.80744
	-	*Clostridiales uncl.*		0.59859
	-	*Coprococcus*		0.58542
	-	*Bacteria uncl.*		0.58435
	-	*Odoribacter*	No-Relapse	0.55470
	-	*Subdoligranulum*		0.53603
	-	*Holdemania*	Healthy	0.52568
	-	*Coprobacter*	Healthy	0.41625
	-	*Fusicatenibacter*		0.41393
	-	*Sutterella*		0.39534
	-	*Bilophila*	Healthy	0.37266
	-	*Dialister*		0.34178
	-	*Paraprevotella*	Healthy	0.33044
	-	*Clostridia uncl.*		0.32762
	-	*Anaerostipes*		0.31833
	-	***Haemophilus***		0.31725
	-	*Alistipes*	No-Relapse	0.27261

	-	*Erysipelotrichaceae uncl.*		0.25180
	-	*Butyricicoccus*		0.25149
	-	*Bacteroidales uncl.*		0.23852
	-	*Bacteroides*		0.22645
Oral	Otu00512	***Kingella***	CRMO	5.14815
	Otu00126	***Kingella***	CRMO	1.53027
	Otu00046	*Streptococcus*	CRMO	0.60750
	Otu00109	***Pasteurellaceae uncl.***		0.51713
	Otu00038	***Haemophilus***		0.46161
	Otu00015	*Rothia*	CRMO	0.45291
	Otu00033	*Veillonella*		0.44075
	Otu00304	*Actinomyces*		0.43888
	Otu00030	*Streptococcus*	CRMO	0.36194
	Otu00068	*Veillonella*	Healthy	0.32744
	Otu00462	*Propionibacteriaceae uncl.*	CRMO	0.32691
	Otu00252	*Prevotella*		0.32002
	Otu00001	*Streptococcus*	Fecal-healthy/Fecal-Relapse	0.31659
	Otu00024	*Rothia*		0.30075
	Otu00200	*Corynebacterium*	CRMO	0.27309
	Otu00130	*Prevotella*		0.26024
	Otu00935	***Pasteurellaceae uncl.***		0.22326
	Otu00548	*Prevotellaceae uncl.*	Healthy	0.20355
	Otu00052	*Porphyromonadaceae uncl.*	Healthy	0.19980
	Otu00004	*Streptococcus*		0.19860
	Otu00007	*Gemella*	Fecal-Relapse	0.19364
	Otu00136	*Neisseria*		0.19355
	Otu00135	*Porphyromonadaceae uncl.*		0.19350
	Otu00463	*Prevotella*		0.18858
	Otu00355	***Pasteurellaceae uncl.***		0.18359
Fecal	Otu00047	*Ruminococcaceae uncl.*	Healthy	0.15949
	Otu00060	*Ruminococcaceae uncl.*	Healthy	0.13304
	Otu00110	*Oscillibacter*	Healthy	0.12736
	Otu00042	*Clostridiales uncl.*		0.09481
	Otu00026	*Oscillibacter*		0.09259
	Otu00089	*Ruminococcaceae uncl.*		0.08894
	Otu00788	*Lachnospiraceae uncl.*	Healthy	0.08235
	Otu00238	*Oscillibacter*	Healthy	0.08116
	Otu00426	*Lachnospiraceae uncl.*	CRMO	0.08052
	Otu00053	*Faecalibacterium*	CRMO	0.07929
	Otu00178	*Ruminococcus*		0.07207
	Otu01297	*Ruminococcaceae uncl.*	Healthy	0.07047
	Otu00208	*Bacteria uncl.*	Healthy	0.06995
	Otu00118	*Coprococcus*	Healthy	0.06865
	Otu00974	*Ruminococcaceae uncl.*	Healthy	0.06700
	Otu00196	*Ruminococcaceae uncl.*	Healthy	0.06689
	Otu00117	*Lachnospiraceae uncl.*	Healthy	0.06504
	Otu00036	*Alistipes*		0.06475
	Otu00013	*Ruminococcaceae uncl.*		0.06462
	Otu00128	*Sutterella*	Healthy	0.05892
	Otu00058	*Ruminococcaceae uncl.*		0.05882
	Otu00587	*Clostridium sensu stricto*		0.05800
	Otu00077	*Dialister*	Healthy	0.05747

	Otu00903	*Holdemania*	Healthy	0.05711
	Otu00360	*Oscillibacter*	Healthy	0.05636

Table S3: Significant taxon centralities in the consensus genus networks of fecal and oral communities based on network permutation (10’000 permutations, one-sided Z test). Potential members of the HACEK-group members are highlighted in bold face and taxa in red mark repeated detections.

Data	Centrality	Genus classification	Z	Observed	*P* (one-sided Z)	Indicator
Oral	Betweeness	*Fusobacteriales uncl.*	5.31500	0.08117	5.33297×10^{-8}	
		Streptococcus	4.53384	0.07277	2.89604×10^{-6}	
		Fusobacterium	2.55654	0.04452	0.00529	
		Campylobacter	2.55403	0.04452	0.00532	CRMO
		SR1 uncl.	2.27059	0.04145	0.01159	
		Proteobacteria uncl.	1.86658	0.03559	0.03098	
		Bacteria uncl.	1.81297	0.03479	0.03492	
		Eikenella	**1.72483**	**0.03439**	**0.04228**	**CRMO**
		Kingella	**1.67379**	**0.03292**	**0.04709**	**CRMO**
	Degree	*Streptococcus*	3.83418	0.36585	0.00006	
		Leptotrichiaceae uncl.	2.90685	0.30081	0.00183	
		Capnocytophaga	2.82413	0.29268	0.00237	
		Campylobacter	2.65533	0.28455	0.00396	CRMO
		Fusobacterium	2.45213	0.26829	0.00710	
		Eikenella	**2.30821**	**0.26016**	**0.01049**	**CRMO**
		Bacteria uncl.	2.10684	0.24390	0.01757	
		Fusobacteriales uncl.	2.09644	0.24390	0.01802	
		Prevotella	1.73242	0.21951	0.04160	
	PageRank	*Streptococcus*	7.60251	0.04849	1.45220×10^{-14}	
		Haemophilus	**1.78710**	**0.01739**	**0.03696**	
		Capnocytophaga	1.76531	0.01732	0.03876	
		Gemella	1.66045	0.01671	0.04841	
Fecal	Betweeness	*Bacteroides*	6.25999	0.12373	1.92505×10^{-10}	
		Paraprevotella	2.54597	0.05562	0.00545	Healthy
		Faecalibacterium	2.33675	0.05449	0.00973	
		Clostridium XVIII	2.21830	0.05149	0.01327	Yes-Relapse
		Coprococcus	2.09234	0.04962	0.01820	
		Gemella	1.87831	0.04674	0.03017	
		Anaerovorax	1.72146	0.04312	0.04258	No-Relapse
		Hungatella	1.65144	0.04262	0.04932	
	Degree	*Bacteroides*	5.74324	0.40157	4.64421×10^{-9}	
		Faecalibacterium	3.69824	0.28346	0.00011	CRMO
		Bacteria uncl.	3.65812	0.27559	0.00013	
		Clostridium XVIII	2.65487	0.22047	0.00397	Yes-Relapse
		Oscillibacter	1.86420	0.17323	0.03115	Healthy, No-Relapse
		Odoribacter	1.67843	0.16535	0.04663	No-Relapse
	PageRank	*Bacteroides*	6.94606	0.04751	1.87819×10^{-12}	
		Faecalibacterium	4.49562	0.03345	3.46840×10^{-6}	CRMO
		Bacteria uncl.	3.63793	0.02793	0.00014	
		Clostridium XVIII	3.03313	0.02470	0.00121	Yes-Relapse
		Clostridiales uncl.	2.27230	0.02052	0.01153	

Table S3: Significant taxon centralities in the species level OTU networks of fecal and oral communities based on network permutation (10’000 permutations, one-sided Z test). Potential members of the HACEK-group members are highlighted in bold face and taxa in red mark repeated detections.

Data	Centrality	OTU-ID	Z	Observed	*P*(one-sided Z)	Phylum	Genus	Indicator
Oral	Betweenness	**OTU-257**	**7.01483**	**0.01860**	**1.15×10^{-12}**	**Proteobacteria**	***Eikenella***	**CRMO**
		OTU-39	5.98563	0.01525	1.08×10^{-9}	Bacteroidetes	*Prevotella*	
		OTU-255	5.64501	0.01468	8.26×10^{-9}	Proteobacteria	*Campylobacter*	CRMO
		OTU-412	4.78521	0.01277	8.54×10^{-7}	Fusobacteria	*Fusobacterium*	
		OTU-130	4.45715	0.01201	4.15×10^{-6}	Bacteroidetes	*Prevotella*	
		OTU-88	3.75790	0.01036	0.00009	Fusobacteria	*Leptotrichiaceae uncl.*	
		OTU-635	3.50893	0.00978	0.00022	Firmicutes	*Selenomonas*	
		OTU-972	3.39123	0.00976	0.00035	Firmicutes	*Streptococcus*	
		OTU-137	3.42315	0.00967	0.00031	Bacteroidetes	*Porphyromonadaceae uncl.*	
		OTU-104	3.13234	0.00943	0.00087	Fusobacteria	*Fusobacterium*	
		OTU-409	3.26081	0.00914	0.00056	Bacteroidetes	*Flavobacteriaceae uncl.*	
		OTU-50	3.00009	0.00911	0.00135	Actinobacteria	*Corynebacterium*	CRMO
		OTU-548	3.04141	0.00892	0.00118	Bacteroidetes	*Prevotellaceae uncl.*	Healthy
		OTU-121	2.93745	0.00883	0.00165	Fusobacteria	*Leptotrichia*	
		OTU-240	2.94976	0.00851	0.00159	Fusobacteria	*Fusobacterium*	
		**OTU-100**	**2.75081**	**0.00837**	**0.00297**	**Proteobacteria**	***Haemophilus***	
		OTU-358	2.65973	0.00808	0.00391	Firmicutes	*Selenomonas*	
		OTU-275	2.76833	0.00802	0.00282	Firmicutes	*Gemella*	
		**OTU-749**	**2.58830**	**0.00790**	**0.00482**	**Proteobacteria**	***Pasteurellaceae uncl.***	
		**OTU-126**	**2.65121**	**0.00789**	**0.00401**	**Proteobacteria**	***Kingella***	**CRMO**
		OTU-210	2.56354	0.00782	0.00518	Proteobacteria	*Campylobacter*	CRMO
		OTU-431	2.47820	0.00781	0.00660	SR1	*SR1 uncl.*	
		OTU-78	2.60280	0.00773	0.00462	Fusobacteria	*Fusobacterium*	
		OTU-353	2.36830	0.00728	0.00893	Actinobacteria	*Corynebacterium*	
		OTU-204	2.25135	0.00727	0.01218	Bacteroidetes	*Capnocytophaga*	
		OTU-232	2.27317	0.00718	0.01151	Bacteroidetes	*Prevotella*	
		**OTU-1025**	**2.23318**	**0.00716**	**0.01277**	**Proteobacteria**	***Pasteurellaceae uncl.***	**Healthy**
		OTU-62	1.89637	0.00678	0.02896	Bacteroidetes	*Porphyromonas*	
		OTU-715	2.09214	0.00662	0.01821	Bacteria uncl.	*Bacteria uncl.*	
		OTU-1022	2.00844	0.00661	0.02230	Fusobacteria	*Leptotrichia*	
		OTU-40	2.02003	0.00658	0.02169	Bacteroidetes	*Porphyromonas*	

	OTU-132	2.02391	0.00658	0.02149	Proteobacteria	*Campylobacter*	
	**OTU-740**	**1.96111**	**0.00655**	**0.02493**	**Proteobacteria**	***Pasteurellaceae uncl.***	**CRMO**
	OTU-162	1.90520	0.00655	0.02838	Actinobacteria	*Actinomyces*	
	OTU-297	1.98380	0.00652	0.02364	Fusobacteria	*Leptotrichiaceae uncl.*	
	OTU-1009	1.99249	0.00651	0.02316	Bacteroidetes	*Flavobacteriaceae uncl.*	Healthy
	OTU-314	1.94760	0.00642	0.02573	Bacteroidetes	*Capnocytophaga*	
	OTU-1018	1.96734	0.00642	0.02457	Bacteroidetes	*Bacteroidetes uncl.*	
	OTU-293	1.88071	0.00640	0.03001	Bacteroidetes	*Prevotella*	
	OTU-469	1.90871	0.00624	0.02815	Bacteria uncl.	*Bacteria uncl.*	
	**OTU-99**	**1.79125**	**0.00622**	**0.03663**	**Proteobacteria**	***Pasteurellaceae uncl.***	
	OTU-296	1.85720	0.00610	0.03164	Bacteroidetes	*Prevotella*	
	**OTU-479**	**1.69040**	**0.00596**	**0.04548**	**Proteobacteria**	***Haemophilus***	
	**OTU-1221**	**1.72569**	**0.00590**	**0.04220**	**Proteobacteria**	***Cardiobacterium***	
	**OTU-512**	**1.67355**	**0.00587**	**0.04711**	**Proteobacteria**	***Kingella***	**CRMO**
	OTU-790	1.73929	0.00586	0.04099	Bacteroidetes	*Porphyromonas*	
	OTU-340	1.67386	0.00586	0.04708	Bacteria uncl.	*Bacteria uncl.*	
	OTU-179	1.75758	0.00581	0.03941	Firmicutes	*Streptococcus*	
Degree	**OTU-257**	**3.74468**	**0.20839**	**0.00009**	**Proteobacteria**	***Eikenella***	**CRMO**
	**OTU-126**	**3.65999**	**0.20116**	**0.00013**	**Proteobacteria**	***Kingella***	**CRMO**
	**OTU-417**	**3.39320**	**0.19392**	**0.00035**	**Proteobacteria**	***Haemophilus***	
	OTU-255	3.10827	0.18090	0.00094	Proteobacteria	*Campylobacter*	CRMO
	OTU-43	3.06498	0.17656	0.00109	Fusobacteria	*Leptotrichia*	CRMO
	OTU-210	3.03738	0.17656	0.00119	Proteobacteria	*Campylobacter*	CRMO
	OTU-55	2.93148	0.17366	0.00169	Bacteroidetes	*Capnocytophaga*	CRMO
	OTU-652	2.95033	0.17366	0.00159	Bacteroidetes	*Bacteroidales uncl.*	Healthy
	**OTU-100**	**2.92474**	**0.17221**	**0.00172**	**Proteobacteria**	***Haemophilus***	
	OTU-327	2.86030	0.16932	0.00212	Bacteroidetes	*Bacteroidales uncl.*	
	OTU-132	2.83173	0.16787	0.00231	Proteobacteria	*Campylobacter*	
	**OTU-538**	**2.79986**	**0.16643**	**0.00256**	**Proteobacteria**	***Haemophilus***	
	OTU-33	2.62956	0.16208	0.00427	Firmicutes	*Veillonella*	
	OTU-39	2.54691	0.15630	0.00543	Bacteroidetes	*Prevotella*	
	OTU-50	2.43571	0.15630	0.00743	Actinobacteria	*Corynebacterium*	CRMO
	OTU-88	2.52356	0.15340	0.00581	Fusobacteria	*Leptotrichiaceae uncl.*	
	OTU-293	2.42143	0.15340	0.00773	Bacteroidetes	*Prevotella*	
	OTU-790	2.52364	0.15340	0.00581	Bacteroidetes	*Porphyromonas*	
	OTU-121	2.37625	0.15195	0.00874	Fusobacteria	*Leptotrichia*	
	**OTU-311**	**2.42309**	**0.15195**	**0.00769**	**Proteobacteria**	***Cardiobacterium***	**CRMO**

	OTU-631	2.44165	0.15195	0.00731	Bacteroidetes	*Bacteroidetes uncl.*	
	OTU-97	2.36158	0.14906	0.00910	Firmicutes	*Streptococcus*	
	OTU-635	2.37157	0.14761	0.00886	Firmicutes	*Selenomonas*	
	**OTU-12**	**2.33426**	**0.14616**	**0.00979**	**Proteobacteria**	***Haemophilus***	
	OTU-409	2.29507	0.14616	0.01086	Bacteroidetes	*Flavobacteriaceae uncl.*	
	OTU-578	2.28016	0.14616	0.01130	Bacteroidetes	*Porphyromonadaceae uncl.*	Healthy
	OTU-814	2.21756	0.14472	0.01329	Bacteroidetes	*Bacteroidetes uncl.*	Healthy
	OTU-1018	2.27704	0.14472	0.01139	Bacteroidetes	*Bacteroidetes uncl.*	
	OTU-1062	2.22848	0.14472	0.01292	Bacteroidetes	*Bacteroidetes uncl.*	
	OTU-115	2.16673	0.14038	0.01513	Actinobacteria	*Actinomyces*	
	OTU-137	2.18679	0.14038	0.01438	Bacteroidetes	*Porphyromonadaceae uncl.*	
	**OTU-149**	**2.08685**	**0.13893**	**0.01845**	**Proteobacteria**	***Haemophilus***	**Yes-Relapse**
	**OTU-401**	**2.10843**	**0.13893**	**0.01750**	**Proteobacteria**	***Haemophilus***	
	OTU-412	2.11745	0.13893	0.01711	Fusobacteria	*Fusobacterium*	
	OTU-52	2.06203	0.13748	0.01960	Bacteroidetes	*Porphyromonadaceae uncl.*	Healthy
	OTU-422	2.08759	0.13748	0.01842	Bacteroidetes	*Bacteroidales uncl.*	
	OTU-135	2.02941	0.13603	0.02121	Bacteroidetes	*Porphyromonadaceae uncl.*	
	OTU-867	2.08180	0.13603	0.01868	Bacteroidetes	*Porphyromonas*	
	OTU-892	2.03591	0.13459	0.02088	Fusobacteria	*Streptobacillus*	
	OTU-536	1.95747	0.13314	0.02515	Bacteroidetes	*Porphyromonadaceae uncl.*	Healthy
	OTU-972	1.93169	0.13169	0.02670	Firmicutes	*Streptococcus*	
	OTU-162	1.87560	0.13025	0.03036	Actinobacteria	*Actinomyces*	
	OTU-240	2.00759	0.13025	0.02234	Fusobacteria	*Fusobacterium*	
	OTU-358	1.85347	0.12735	0.03191	Firmicutes	*Selenomonas*	
	OTU-462	1.89486	0.12735	0.02906	Actinobacteria	*Propionibacteriaceae uncl.*	CRMO
	**OTU-512**	**1.81875**	**0.12735**	**0.03447**	**Proteobacteria**	***Kingella***	**CRMO**
	OTU-46	1.82351	0.12590	0.03411	Firmicutes	*Streptococcus*	CRMO
	OTU-104	1.81862	0.12590	0.03448	Fusobacteria	*Fusobacterium*	
	OTU-324	1.83547	0.12590	0.03322	Firmicutes	*Lachnoanaerobaculum*	CRMO
	OTU-78	1.77748	0.12446	0.03774	Fusobacteria	*Fusobacterium*	
	OTU-91	1.77529	0.12446	0.03792	Firmicutes	*Streptococcus*	
	OTU-553	1.71990	0.12301	0.04273	Firmicutes	*Veillonella*	
	**OTU-158**	**1.66536**	**0.12156**	**0.04792**	**Proteobacteria**	***Pasteurellaceae uncl.***	
	OTU-275	1.72737	0.12156	0.04205	Firmicutes	*Gemella*	
	OTU-51	1.71330	0.12012	0.04333	Proteobacteria	*Lautropia*	
	OTU-68	1.70005	0.12012	0.04456	Firmicutes	*Veillonella*	Healthy

	OTU-94	1.68118	0.12012	0.04636	Bacteroidetes	*Capnocytophaga*	
	OTU-516	1.68103	0.12012	0.04638	Bacteroidetes	*Bacteroidetes uncl.*	
	OTU-264	1.66064	0.11867	0.04839	Bacteroidetes	*Capnocytophaga*	
	OTU-296	1.65631	0.11867	0.04883	Bacteroidetes	*Prevotella*	
	**OTU-730**	**1.65849**	**0.11867**	**0.04861**	**Proteobacteria**	***Haemophilus***	
PageRank	**OTU-417**	**2.93885**	**0.00451**	**0.00165**	**Proteobacteria**	***Haemophilus***	
	OTU-327	2.85214	0.00437	0.00217	Bacteroidetes	*Bacteroidales uncl.*	
	**OTU-538**	**2.80779**	**0.00432**	**0.00249**	**Proteobacteria**	***Haemophilus***	
	**OTU-100**	**2.67949**	**0.00421**	**0.00369**	**Proteobacteria**	***Haemophilus***	
	OTU-652	2.49208	0.00401	0.00635	Bacteroidetes	*Bacteroidales uncl.*	Healthy
	OTU-422	2.45905	0.00396	0.00697	Bacteroidetes	*Bacteroidales uncl.*	
	**OTU-12**	**2.44515**	**0.00392**	**0.00724**	**Proteobacteria**	***Haemophilus***	
	OTU-210	2.39445	0.00391	0.00832	Proteobacteria	*Campylobacter*	CRMO
	OTU-137	2.35719	0.00383	0.00921	Bacteroidetes	*Porphyromonadaceae uncl.*	
	OTU-50	2.22299	0.00382	0.01311	Actinobacteria	*Corynebacterium*	CRMO
	OTU-536	2.25334	0.00378	0.01212	Bacteroidetes	*Porphyromonadaceae uncl.*	Healthy
	OTU-88	2.26378	0.00372	0.01179	Fusobacteria	*Leptotrichiaceae uncl.*	
	OTU-578	2.17505	0.00371	0.01481	Bacteroidetes	*Porphyromonadaceae uncl.*	Healthy
	**OTU-149**	**2.15508**	**0.00369**	**0.01558**	**Proteobacteria**	***Haemophilus***	**Yes-Relapse**
	OTU-516	2.15843	0.00364	0.01545	Bacteroidetes	*Bacteroidetes uncl.*	
	OTU-121	2.04898	0.00360	0.02023	Fusobacteria	*Leptotrichia*	
	OTU-52	2.05281	0.00358	0.02005	Bacteroidetes	*Porphyromonadaceae uncl.*	Healthy
	**OTU-311**	**2.03772**	**0.00355**	**0.02079**	**Proteobacteria**	***Cardiobacterium***	**CRMO**
	OTU-462	2.10329	0.00354	0.01772	Actinobacteria	*Propionibacteriaceae uncl.*	CRMO
	OTU-631	2.06075	0.00354	0.01966	Bacteroidetes	*Bacteroidetes uncl.*	
	OTU-1062	2.01250	0.00354	0.02208	Bacteroidetes	*Bacteroidetes uncl.*	
	**OTU-126**	**2.01481**	**0.00350**	**0.02196**	**Proteobacteria**	***Kingella***	**CRMO**
	OTU-255	1.97341	0.00349	0.02422	Proteobacteria	*Campylobacter*	CRMO
	**OTU-158**	**1.94885**	**0.00348**	**0.02566**	**Proteobacteria**	***Pasteurellaceae uncl.***	
	OTU-135	1.95697	0.00348	0.02518	Bacteroidetes	*Porphyromonadaceae uncl.*	
	OTU-867	1.94053	0.00341	0.02616	Bacteroidetes	*Porphyromonas*	
	OTU-55	1.88568	0.00341	0.02967	Bacteroidetes	*Capnocytophaga*	CRMO
	**OTU-257**	**1.82665**	**0.00336**	**0.03388**	**Proteobacteria**	***Eikenella***	**CRMO**
	OTU-892	1.88095	0.00336	0.02999	Fusobacteria	*Streptobacillus*	
	OTU-445	1.84580	0.00336	0.03246	Bacteroidetes	*Bacteroidales uncl.*	
	**OTU-401**	**1.84765**	**0.00336**	**0.03233**	**Proteobacteria**	***Haemophilus***	

		OTU-814	1.79598	0.00333	0.03625	Bacteroidetes	*Bacteroidetes uncl.*	Healthy
		OTU-368	1.81743	0.00333	0.03458	Proteobacteria	*Actinobacillus*	
		OTU-162	1.78646	0.00332	0.03701	Actinobacteria	*Actinomyces*	
		OTU-97	1.79183	0.00328	0.03658	Firmicutes	*Streptococcus*	
		OTU-68	1.79773	0.00327	0.03611	Firmicutes	*Veillonella*	Healthy
		OTU-91	1.78807	0.00327	0.03688	Firmicutes	*Streptococcus*	
		OTU-297	1.75222	0.00324	0.03987	Fusobacteria	*Leptotrichiaceae uncl.*	
		OTU-409	1.74653	0.00324	0.04036	Bacteroidetes	*Flavobacteriaceae uncl.*	
		OTU-790	1.76763	0.00321	0.03856	Bacteroidetes	*Porphyromonas*	
		OTU-10	1.68485	0.00319	0.04601	Bacteroidetes	*Prevotellaceae uncl.*	
		OTU-546	1.67180	0.00316	0.04728	Firmicutes	*Clostridiales uncl.*	Healthy
		**OTU-464**	**1.64721**	**0.00315**	**0.04976**	**Proteobacteria**	***Pasteurellaceae uncl.***	
		OTU-51	1.69830	0.00314	0.04473	Proteobacteria	*Lautropia*	
		OTU-469	1.64995	0.00314	0.04948	Bacteria uncl.	*Bacteria uncl.*	
Fecal	Betweenness	OTU-191	11.27275	0.01762	8.94×10^{-30}	Bacteria uncl.	*Bacteria uncl.*	
		OTU-303	6.16100	0.00929	3.61×10^{-10}	Firmicutes	*Ruminococcaceae uncl.*	
		OTU-113	5.55280	0.00927	1.41×10^{-8}	Bacteria uncl.	*Bacteria uncl.*	
		OTU-250	5.27608	0.00835	6.60×10^{-8}	Bacteroidetes	*Paraprevotella*	Healthy
		OTU-392	5.18111	0.00829	1.10×10^{-7}	Firmicutes	*Ruminococcaceae uncl.*	
		OTU-174	5.07580	0.00819	1.93×10^{-7}	Firmicutes	*Lachnospiraceae uncl.*	
		OTU-26	4.96332	0.00807	3.46×10^{-7}	Firmicutes	*Oscillibacter*	
		OTU-79	4.81109	0.00806	7.51×10^{-7}	Bacteria uncl.	*Bacteria uncl.*	No-Relapse
		OTU-47	5.07334	0.00802	1.95×10^{-7}	Firmicutes	*Ruminococcaceae uncl.*	Healthy
		OTU-1156	5.21938	0.00796	8.98×10^{-8}	Firmicutes	*Lachnospiraceae uncl.*	
		OTU-378	4.48174	0.00786	3.70×10^{-6}	Firmicutes	*Ruminococcaceae uncl.*	
		OTU-101	4.39616	0.00766	0.00001	Firmicutes	*Ruminococcaceae uncl.*	
		OTU-42	4.52332	0.00733	3.04×10^{-6}	Firmicutes	*Clostridiales uncl.*	
		OTU-1246	4.36532	0.00694	0.00001	Firmicutes	*Lachnospiraceae uncl.*	Yes-Relapse
		OTU-809	3.75619	0.00682	0.00009	Firmicutes	*Clostridium XVIII*	Yes-Relapse
		OTU-393	4.04025	0.00662	0.00003	Firmicutes	*Ruminococcaceae uncl.*	
		OTU-287	4.01804	0.00660	0.00003	Firmicutes	*Lachnospiraceae uncl.*	
		OTU-89	3.66595	0.00640	0.00012	Firmicutes	*Ruminococcaceae uncl.*	
		OTU-502	3.68947	0.00634	0.00011	Firmicutes	*Ruminococcaceae uncl.*	
		OTU-592	3.39270	0.00584	0.00035	Firmicutes	*Clostridiales Incertae Sedis XIII*	

						uncl.	
	OTU-525	3.18927	0.00581	0.00071	Firmicutes	*Ruminococcaceae uncl.*	
	OTU-241	2.91234	0.00546	0.00179	Firmicutes	*Lachnospiraceae uncl.*	
	OTU-735	2.73348	0.00532	0.00313	Firmicutes	*Faecalibacterium*	CRMO
	OTU-1105	2.90925	0.00522	0.00181	Bacteroidetes	*Bacteroides*	
	OTU-1303	2.90499	0.00522	0.00184	Firmicutes	*Faecalibacterium*	
	OTU-453	2.77065	0.00518	0.00280	Proteobacteria	*Oxalobacter*	
	OTU-14	2.81333	0.00514	0.00245	Firmicutes	*Faecalibacterium*	CRMO
	OTU-750	2.86400	0.00506	0.00209	Firmicutes	*Clostridiales uncl.*	
	OTU-506	2.73630	0.00492	0.00311	Firmicutes	*Ruminococcaceae uncl.*	
	OTU-180	2.56317	0.00484	0.00519	Firmicutes	*Lachnospiraceae uncl.*	
	OTU-449	2.58920	0.00483	0.00481	Bacteroidetes	*Bacteroides*	
	OTU-1031	2.39604	0.00467	0.00829	Bacteria uncl.	*Bacteria uncl.*	No-Relapse
	OTU-363	2.57771	0.00465	0.00497	Firmicutes	*Ruminococcaceae uncl.*	Healthy
	OTU-2044	2.39531	0.00453	0.00830	Firmicutes	*Faecalibacterium*	CRMO
	OTU-254	2.39505	0.00453	0.00831	Firmicutes	*Clostridiales uncl.*	
	OTU-984	2.37305	0.00452	0.00882	Firmicutes	*Fusicatenibacter*	Yes-Relapse
	OTU-168	2.43159	0.00447	0.00752	Firmicutes	*Ruminococcaceae uncl.*	
	OTU-373	2.14836	0.00445	0.01584	Bacteroidetes	*Porphyromonadaceae uncl.*	Healthy
	OTU-329	2.35199	0.00443	0.00934	Firmicutes	*Ruminococcaceae uncl.*	
	OTU-348	2.26368	0.00434	0.01180	Firmicutes	*Clostridiales uncl.*	
	OTU-20	2.11373	0.00433	0.01727	Bacteroidetes	*Prevotella*	Healthy
	OTU-1147	2.19642	0.00423	0.01403	Firmicutes	*Subdoligranulum*	
	OTU-428	2.18608	0.00420	0.01440	Firmicutes	*Flavonifractor*	
	OTU-2024	2.07470	0.00414	0.01901	Firmicutes	*Ruminococcaceae uncl.*	
	OTU-1421	2.09472	0.00413	0.01810	Bacteria uncl.	*Bacteria uncl.*	
	OTU-110	2.09935	0.00412	0.01789	Firmicutes	*Oscillibacter*	Healthy
	OTU-571	1.99453	0.00407	0.02305	Firmicutes	*Faecalibacterium*	
	OTU-341	1.93791	0.00401	0.02632	Firmicutes	*Coprococcus*	
	OTU-1126	1.95167	0.00395	0.02549	Firmicutes	*Ruminococcaceae uncl.*	
	OTU-1672	2.01767	0.00395	0.02181	Firmicutes	*Faecalibacterium*	
	OTU-1010	1.93107	0.00393	0.02674	Firmicutes	*Ruminococcaceae uncl.*	
	OTU-904	1.92250	0.00393	0.02727	Firmicutes	*Firmicutes uncl.*	
	OTU-77	1.86406	0.00390	0.03116	Firmicutes	*Dialister*	Healthy
	OTU-102	1.80414	0.00390	0.03560	Firmicutes	*Oscillibacter*	

	OTU-807	1.87962	0.00390	0.03008	Firmicutes	*Faecalibacterium*	
	OTU-309	1.85709	0.00390	0.03165	Firmicutes	*Ruminococcaceae uncl.*	
	OTU-507	1.86804	0.00389	0.03088	Firmicutes	*Ruminococcaceae uncl.*	Healthy
	OTU-193	2.00097	0.00389	0.02270	Bacteria uncl.	*Bacteria uncl.*	
	OTU-323	1.89595	0.00388	0.02898	Firmicutes	*Lachnospiraceae uncl.*	
	OTU-802	1.85614	0.00387	0.03172	Firmicutes	*Roseburia*	
	OTU-2653	1.74242	0.00385	0.04072	Firmicutes	*Roseburia*	
	OTU-451	1.89025	0.00384	0.02936	Firmicutes	*Clostridiales uncl.*	
	OTU-1095	1.76526	0.00382	0.03876	Bacteria uncl.	*Bacteria uncl.*	
	OTU-118	1.78237	0.00382	0.03734	Firmicutes	*Coprococcus*	Healthy
	OTU-577	1.82431	0.00382	0.03405	Bacteria uncl.	*Bacteria uncl.*	
	OTU-151	1.76781	0.00377	0.03855	Firmicutes	*Ruminococcaceae uncl.*	
	OTU-832	1.74439	0.00376	0.04055	Firmicutes	*Lachnospiraceae uncl.*	
	OTU-687	1.75961	0.00374	0.03924	Firmicutes	*Lachnospiraceae uncl.*	Yes-Relapse
	OTU-2020	1.78989	0.00372	0.03674	Bacteroidetes	*Bacteroides*	Yes-Relapse
	OTU-152	1.79488	0.00372	0.03634	Bacteroidetes	*Odoribacter*	No-Relapse
	OTU-172	1.70949	0.00363	0.04368	Firmicutes	*Blautia*	
	OTU-442	1.86639	0.00363	0.03099	Firmicutes	*Ruminococcaceae uncl.*	
	OTU-116	1.72890	0.00362	0.04191	Firmicutes	*Roseburia*	
	OTU-530	1.66234	0.00361	0.04822	Firmicutes	*Clostridiales uncl.*	Healthy
	OTU-103	1.74409	0.00361	0.04057	Firmicutes	*Clostridiales uncl.*	
	OTU-328	1.66377	0.00359	0.04808	Proteobacteria	*Bilophila*	Healthy
Degree	OTU-26	7.15005	0.15108	4.33743×10^{-13}	Firmicutes	*Oscillibacter*	
	OTU-191	6.89925	0.14868	2.61386×10^{-12}	Bacteria uncl.	*Bacteria uncl.*	
	OTU-47	6.81083	0.13989	4.8517×10^{-12}	Firmicutes	*Ruminococcaceae uncl.*	Healthy
	OTU-79	5.02310	0.11431	2.5422×10^{-7}	Bacteria uncl.	*Bacteria uncl.*	No-Relapse
	OTU-42	4.62425	0.10711	1.87976×10^{-6}	Firmicutes	*Clostridiales uncl.*	
	OTU-113	4.48053	0.10392	3.72293×10^{-6}	Bacteria uncl.	*Bacteria uncl.*	
	OTU-303	4.33684	0.09912	0.00001	Firmicutes	*Ruminococcaceae uncl.*	
	OTU-329	4.20460	0.09752	0.00001	Firmicutes	*Ruminococcaceae uncl.*	
	OTU-14	4.13205	0.09592	0.00002	Firmicutes	*Faecalibacterium*	CRMO
	OTU-110	4.16697	0.09592	0.00002	Firmicutes	*Oscillibacter*	Healthy
	OTU-392	3.92523	0.09193	0.00004	Firmicutes	*Ruminococcaceae uncl.*	

	OTU-449	3.63430	0.08873	0.00014	Bacteroidetes	*Bacteroides*	
	OTU-502	3.47881	0.08633	0.00025	Firmicutes	*Ruminococcaceae uncl.*	
	OTU-174	3.45364	0.08553	0.00028	Firmicutes	*Lachnospiraceae uncl.*	
	OTU-525	3.16020	0.08153	0.00079	Firmicutes	*Ruminococcaceae uncl.*	
	OTU-323	3.15596	0.08074	0.00080	Firmicutes	*Lachnospiraceae uncl.*	
	OTU-378	3.09310	0.08074	0.00099	Firmicutes	*Ruminococcaceae uncl.*	
	OTU-592	3.18610	0.08074	0.00072	Firmicutes	*Clostridiales Incertae Sedis XIII uncl.*	
	OTU-101	2.93859	0.07914	0.00165	Firmicutes	*Ruminococcaceae uncl.*	
	OTU-506	3.12080	0.07914	0.00090	Firmicutes	*Ruminococcaceae uncl.*	
	OTU-809	2.90314	0.07834	0.00185	Firmicutes	*Clostridium XVIII*	Yes-Relapse
	OTU-735	2.87685	0.07754	0.00201	Firmicutes	*Faecalibacterium*	CRMO
	OTU-118	2.85360	0.07594	0.00216	Firmicutes	*Coprococcus*	Healthy
	OTU-161	2.89913	0.07594	0.00187	Firmicutes	*Lachnospiraceae uncl.*	
	OTU-60	2.90538	0.07274	0.00183	Firmicutes	*Ruminococcaceae uncl.*	Healthy
	OTU-102	2.70896	0.07274	0.00337	Firmicutes	*Oscillibacter*	
	OTU-442	2.74220	0.07114	0.00305	Firmicutes	*Ruminococcaceae uncl.*	
	OTU-89	2.64702	0.07034	0.00406	Firmicutes	*Ruminococcaceae uncl.*	
	OTU-241	2.63291	0.07034	0.00423	Firmicutes	*Lachnospiraceae uncl.*	
	OTU-250	2.61636	0.06954	0.00444	Bacteroidetes	*Paraprevotella*	Healthy
	OTU-20	2.40168	0.06875	0.00816	Bacteroidetes	*Prevotella*	Healthy
	OTU-328	2.50915	0.06875	0.00605	Proteobacteria	*Bilophila*	Healthy
	OTU-393	2.59789	0.06875	0.00469	Firmicutes	*Ruminococcaceae uncl.*	
	OTU-216	2.43297	0.06635	0.00749	Firmicutes	*Lachnospiraceae uncl.*	Healthy
	OTU-430	2.48756	0.06635	0.00643	Firmicutes	*Faecalibacterium*	CRMO
	OTU-1105	2.49471	0.06635	0.00630	Bacteroidetes	*Bacteroides*	
	OTU-229	2.35108	0.06555	0.00936	Firmicutes	*Butyricicoccus*	No-Relapse
	OTU-75	2.37365	0.06395	0.00881	Firmicutes	*Lachnospiraceae uncl.*	
	OTU-567	2.18965	0.06395	0.01427	Firmicutes	*Ruminococcaceae uncl.*	No-Relapse
	OTU-597	2.19843	0.06395	0.01396	Firmicutes	*Oscillibacter*	
	OTU-348	2.24019	0.06315	0.01254	Firmicutes	*Clostridiales uncl.*	
	OTU-687	2.04035	0.06155	0.02066	Firmicutes	*Lachnospiraceae uncl.*	Yes-Relapse
	OTU-76	2.05627	0.06075	0.01988	Bacteroidetes	*Parabacteroides*	

	OTU-287	2.13969	0.06075	0.01619	Firmicutes	*Lachnospiraceae uncl.*	
	OTU-507	2.01873	0.05995	0.02176	Firmicutes	*Ruminococcaceae uncl.*	Healthy
	OTU-571	2.04885	0.05995	0.02024	Firmicutes	*Faecalibacterium*	
	OTU-230	1.99144	0.05835	0.02322	Firmicutes	*Clostridiales uncl.*	
	OTU-523	1.89650	0.05835	0.02895	Firmicutes	*Lachnospiraceae uncl.*	Healthy
	OTU-177	1.86642	0.05755	0.03099	Firmicutes	*Ruminococcaceae uncl.*	
	OTU-282	1.83841	0.05755	0.03300	Firmicutes	*Ruminococcaceae uncl.*	
	OTU-83	1.84998	0.05675	0.03216	Firmicutes	*Roseburia*	
	OTU-128	1.80700	0.05675	0.03538	Proteobacteria	*Sutterella*	Healthy
	OTU-869	1.82989	0.05675	0.03363	Firmicutes	*Faecalibacterium*	
	OTU-1040	1.83585	0.05675	0.03319	Firmicutes	*Roseburia*	
	OTU-1148	1.82735	0.05675	0.03382	Firmicutes	*Lachnospiraceae uncl.*	
	OTU-1246	1.93719	0.05675	0.02636	Firmicutes	*Lachnospiraceae uncl.*	Yes-Relapse
	OTU-2020	1.90142	0.05675	0.02862	Bacteroidetes	*Bacteroides*	Yes-Relapse
	OTU-63	1.80395	0.05516	0.03562	Firmicutes	*Roseburia*	
	OTU-363	1.83123	0.05516	0.03353	Firmicutes	*Ruminococcaceae uncl.*	Healthy
	OTU-103	1.70411	0.05436	0.04418	Firmicutes	*Clostridiales uncl.*	
	OTU-577	1.72573	0.05436	0.04220	Bacteria uncl.	*Bacteria uncl.*	
	OTU-802	1.71842	0.05436	0.04286	Firmicutes	*Roseburia*	
	OTU-844	1.72321	0.05436	0.04243	Bacteroidetes	*Bacteroides*	
	OTU-58	1.71698	0.05356	0.04299	Firmicutes	*Ruminococcaceae uncl.*	
	OTU-196	1.69503	0.05356	0.04503	Firmicutes	*Ruminococcaceae uncl.*	Healthy
	OTU-299	1.72137	0.05356	0.04259	Bacteroidetes	*Parabacteroides*	Yes-Relapse
	OTU-309	1.68142	0.05356	0.04634	Firmicutes	*Ruminococcaceae uncl.*	
	OTU-410	1.68161	0.05356	0.04632	Firmicutes	*Faecalibacterium*	
	OTU-568	1.73073	0.05356	0.04175	Firmicutes	*Ruminococcaceae uncl.*	
	OTU-451	1.65329	0.05276	0.04914	Firmicutes	*Clostridiales uncl.*	
	OTU-1226	1.67647	0.05276	0.04682	Firmicutes	*Lachnospiraceae uncl.*	
	OTU-234	1.67180	0.05196	0.04728	Firmicutes	*Ruminococcaceae uncl.*	
PageRank	OTU-14	4.21629	0.00304	0.00001	Firmicutes	*Faecalibacterium*	CRMO
	OTU-47	4.08482	0.00292	0.00002	Firmicutes	*Ruminococcaceae uncl.*	Healthy
	OTU-191	3.63633	0.00282	0.00014	Bacteria uncl.	*Bacteria uncl.*	
	OTU-449	3.65752	0.00280	0.00013	Bacteroidetes	*Bacteroides*	
	OTU-26	3.66198	0.00279	0.00013	Firmicutes	*Oscillibacter*	

	OTU-303	3.24508	0.00252	0.00059	Firmicutes	*Ruminococcaceae uncl.*	
	OTU-392	3.13736	0.00246	0.00085	Firmicutes	*Ruminococcaceae uncl.*	
	OTU-809	2.86710	0.00245	0.00207	Firmicutes	*Clostridium XVIII*	Yes-Relapse
	OTU-113	2.91604	0.00239	0.00177	Bacteria uncl.	*Bacteria uncl.*	
	OTU-329	2.80836	0.00230	0.00249	Firmicutes	*Ruminococcaceae uncl.*	
	OTU-323	2.71000	0.00230	0.00336	Firmicutes	*Lachnospiraceae uncl.*	
	OTU-502	2.66945	0.00227	0.00380	Firmicutes	*Ruminococcaceae uncl.*	
	OTU-79	2.67419	0.00227	0.00375	Bacteria uncl.	*Bacteria uncl.*	No-Relapse CRMO
	OTU-430	2.68529	0.00220	0.00362	Firmicutes	*Faecalibacterium*	
	OTU-42	2.50421	0.00219	0.00614	Firmicutes	*Clostridiales uncl.*	
	OTU-177	2.47282	0.00217	0.00670	Firmicutes	*Ruminococcaceae uncl.*	
	OTU-506	2.48203	0.00215	0.00653	Firmicutes	*Ruminococcaceae uncl.*	
	OTU-378	2.37904	0.00214	0.00868	Firmicutes	*Ruminococcaceae uncl.*	
	OTU-282	2.38038	0.00213	0.00865	Firmicutes	*Ruminococcaceae uncl.*	
	OTU-161	2.38768	0.00211	0.00848	Firmicutes	*Lachnospiraceae uncl.*	
	OTU-254	2.38988	0.00210	0.00843	Firmicutes	*Clostridiales uncl.*	
	OTU-326	2.38353	0.00208	0.00857	Bacteroidetes	*Bacteroides*	
	OTU-735	2.19237	0.00206	0.01418	Firmicutes	*Faecalibacterium*	CRMO
	OTU-1040	2.26152	0.00204	0.01186	Firmicutes	*Roseburia*	
	OTU-80	2.23405	0.00203	0.01274	Firmicutes	*Clostridiales uncl.*	
	OTU-410	2.28853	0.00203	0.01105	Firmicutes	*Faecalibacterium*	
	OTU-398	2.13614	0.00201	0.01633	Firmicutes	*Ruminococcaceae uncl.*	
	OTU-250	2.25121	0.00200	0.01219	Bacteroidetes	*Paraprevotella*	Healthy
	OTU-234	2.31380	0.00200	0.01034	Firmicutes	*Ruminococcaceae uncl.*	
	OTU-174	2.17828	0.00199	0.01469	Firmicutes	*Lachnospiraceae uncl.*	
	OTU-687	2.10309	0.00199	0.01773	Firmicutes	*Lachnospiraceae uncl.*	Yes-Relapse
	OTU-525	2.12970	0.00199	0.01660	Firmicutes	*Ruminococcaceae uncl.*	
	OTU-1105	2.27409	0.00198	0.01148	Bacteroidetes	*Bacteroides*	
	OTU-20	2.00570	0.00196	0.02244	Bacteroidetes	*Prevotella*	Healthy
	OTU-411	2.14371	0.00193	0.01603	Bacteroidetes	*Bacteroides*	
	OTU-887	2.06376	0.00191	0.01952	Firmicutes	*Ruminococcaceae uncl.*	
	OTU-705	2.02547	0.00191	0.02141	Firmicutes	*Ruminococcaceae uncl.*	
	OTU-1226	2.08280	0.00190	0.01863	Firmicutes	*Lachnospiraceae uncl.*	
	OTU-408	1.98905	0.00189	0.02335	Firmicutes	*Faecalibacterium*	

		OTU-110	2.06746	0.00189	0.01935	Firmicutes	*Oscillibacter*	Healthy
		OTU-1148	1.94954	0.00187	0.02562	Firmicutes	*Lachnospiraceae uncl.*	
		OTU-568	2.05107	0.00187	0.02013	Firmicutes	*Ruminococcaceae uncl.*	
		OTU-508	1.90887	0.00187	0.02814	Firmicutes	*Clostridium XlVa*	
		OTU-241	1.94029	0.00185	0.02617	Firmicutes	*Lachnospiraceae uncl.*	
		OTU-897	1.92779	0.00184	0.02694	Firmicutes	*Faecalibacterium*	
		OTU-451	1.93656	0.00184	0.02640	Firmicutes	*Clostridiales uncl.*	
		OTU-703	1.84853	0.00182	0.03226	Firmicutes	*Ruminococcaceae uncl.*	
		OTU-802	1.87758	0.00182	0.03022	Firmicutes	*Roseburia*	
		OTU-1061	1.76844	0.00179	0.03849	Firmicutes	*Roseburia*	
		OTU-1048	1.85426	0.00179	0.03185	Firmicutes	*Faecalibacterium*	
		OTU-92	1.83576	0.00179	0.03320	Bacteria uncl.	*Bacteria uncl.*	
		OTU-1094	1.89046	0.00178	0.02935	Firmicutes	*Lachnospiraceae uncl.*	
		OTU-348	1.81841	0.00178	0.03450	Firmicutes	*Clostridiales uncl.*	
		OTU-577	1.77688	0.00176	0.03779	Bacteria uncl.	*Bacteria uncl.*	
		OTU-567	1.71011	0.00176	0.04362	Firmicutes	*Ruminococcaceae uncl.*	No-Relapse
		OTU-83	1.77139	0.00176	0.03825	Firmicutes	*Roseburia*	
		OTU-363	1.80330	0.00174	0.03567	Firmicutes	*Ruminococcaceae uncl.*	Healthy
		OTU-592	1.71526	0.00174	0.04315	Firmicutes	*Clostridiales Incertae Sedis XIII uncl.*	
		OTU-171	1.79512	0.00174	0.03632	Firmicutes	*Blautia*	
		OTU-1112	1.67891	0.00173	0.04658	Firmicutes	*Ruminococcaceae uncl.*	
		OTU-571	1.68691	0.00171	0.04581	Firmicutes	*Faecalibacterium*	
		OTU-781	1.70975	0.00169	0.04366	Firmicutes	*Oscillibacter*	
		OTU-63	1.67632	0.00169	0.04684	Firmicutes	*Roseburia*	
		OTU-894	1.64500	0.00169	0.04999	Firmicutes	*Oscillibacter*	
		OTU-442	1.65897	0.00167	0.04856	Firmicutes	*Ruminococcaceae uncl.*	

Table S5: Consensus genera associated to clinical measurements CRMO fecal and oral communities via Euclidean distance correlation and Spearman rank correlation (SCC-Spearman correlation coefficient). Potential HACEK-group members are highlighted in **bold** face and red highlights taxa with multiple associations. The *P*-value cutoff was set to *P*≤0.010.

	Physiology	ID Genus	Eucl. dist. corr.	*P*-value	P_{FDR}	*SCC*	N	Indicator
Fecal	BSR (1h)	*Coprobacter*	0.5872	0.0056	0.5192	0.3592	21	Healthy
	CRP (mg/l)	*Catabacter*	0.6255	0.0093	0.2964	0.3470	22	
	Hematocrit	*Catabacter*	0.6615	0.0024	0.2458	-0.4493	22	
	MCH (pg/cell)	*Clostridiaceae 1 uncl.*	0.6783	0.0011	0.1173	0.6076	21	
		Clostridium sensu stricto	0.6166	0.0047	0.2402	0.3467	21	
	Eosinophiles %	*Porphyromonas*	0.6248	0.0050	0.1012	0.4707	20	
		Ezakiella	0.6648	0.0007	0.0673	0.6152	20	
		Murdochiella	0.7122	0.0022	0.0711	0.5220	20	
		Negativicoccus	0.6628	0.0028	0.0711	0.5220	20	
		Campylobacter	0.6836	0.0019	0.0711	0.5416	20	
	Basophiles %	*Fusobacterium*	0.7217	0.0042	0.3968	0.7301	19	
		Campylobacter	0.6914	0.0078	0.3968	0.6207	19	
	Lymphocytes %	*Bilophila*	0.6179	0.0065	0.5447	-0.6184	20	Healthy
	LDH (U/l)	*Clostridium XlVa*	0.6845	0.0014	0.1418	0.3988	20	
Oral	MCV (fl)	*Bacteria uncl.*	0.5945	0.0069	0.1801	0.3644	24	
	MCH (pg/cell)	*Propionibacteriaceae uncl.*	0.5790	0.0056	0.3767	0.2540	23	CRMO
		Bacteria uncl.	0.5695	0.0082	0.3767	0.4187	23	
	Eosinophiles %	*Flavobacteriales uncl.*	0.5739	0.0088	0.6066	0.4124	22	
	Basophiles %	*Burkholderiales uncl.*	0.5711	0.0100	0.2089	0.4153	21	
		Kingella*	0.6750	0.0064	0.2089	0.1029	21	CRMO
	Lymphocytes %	*Scardovia*	0.5404	0.0084	0.0557	0.4752	22	
		Parabacteroides	0.6074	0.0058	0.0557	0.4990	22	
		Chitinophagaceae uncl.	0.5033	0.0049	0.0557	0.4990	22	
		Staphylococcus	0.6144	0.0080	0.0557	0.2602	22	
		Lactobacillus	0.5116	0.0038	0.0557	0.4431	22	
		Catonella	0.6569	0.0005	0.0359	0.4972	22	
		Lachnospiraceae uncl.	0.6478	0.0012	0.0368	0.4495	22	
		Ruminococcaceae uncl.	0.5340	0.0069	0.0557	0.2374	22	
		Dialister	0.5619	0.0019	0.0428	0.3685	22	
		Megasphaera	0.5734	0.0085	0.0557	0.5004	22	
		Fusobacterium	0.6002	0.0080	0.0557	0.3266	22	
		Erwinia	0.5111	0.0051	0.0557	0.4990	22	
		Alcanivorax	0.5385	0.0036	0.0557	0.4990	22	
		Pseudomonas	0.5651	0.0008	0.0359	0.3843	22	
	Monocytes %	*Clostridiales uncl.*	0.5957	0.0031	0.1444	-0.3288	21	
		Ottowia	0.5248	0.0011	0.1049	0.5238	21	
	Calcium (mmol/l)	*Bacteria uncl.*	0.7727	0.0000	0.0028	0.7052	22	
		Porphyromonas	0.6257	0.0046	0.1408	0.3560	22	
		Gemella	0.6409	0.0023	0.1044	-0.4660	22	

Table S6: Species level OTUs from CRMO fecal communities associated to clinical measurements via Euclidean distance correlation and Spearman rank correlation (SCC-Spearman correlation coefficient). Indicators for healthy individuals are marked with +; # indicators for CRMO; ‡ indicators for relapse; † indicators for no relapse. HACEK-group members are highlighted in **bold** face and red indicates OTUs with multiple associations. The *P*-value cutoff was set to *P*≤0.010.

Physiology	ID	Eucl. dist. corr.	*P*	P_{FDR}	*SCC*	RDP 16 Classification
AP (U/l)	OTU-337	0.7428	0.0048	1.0000	-0.8398	*Firmicutes(100);Clostridia(100);Clostridiales(100);Lachnospiraceae(100);Lachnospiraceae uncl.(85);*
Basophiles %	OTU-6651	0.6835	0.0075	1.0000	0.5135	*Firmicutes(100);Clostridia(100);Clostridiales(100);Lachnospiraceae(100);Lachnospiraceae uncl.(100);*
	OTU-3008	0.8045	0.0015	1.0000	0.5456	*Firmicutes(100);Clostridia(100);Clostridiales(100);Clostridiales uncl.(100);Clostridiales uncl.(100);*
	OTU-1017	0.6862	0.0078	1.0000	0.5731	*Bacteroidetes(100);Bacteroidia(100);Bacteroidales(100);Bacteroidaceae(100);Bacteroides(100);*
BSR (1h)	OTU-1497	0.6529	0.0013	0.2608	-0.6448	*Firmicutes(100);Clostridia(100);Clostridiales(100);Lachnospiraceae(100);Lachnospiraceae uncl.(100);*
	OTU-3565	0.5572	0.0043	0.4174	-0.6076	*Firmicutes(100);Clostridia(100);Clostridiales(100);Ruminococcaceae(80);Ruminococcaceae uncl.(80);*
	OTU-5198	0.5349	0.0085	0.5403	-0.5513	*Firmicutes(100);Clostridia(100);Clostridiales(100);Lachnospiraceae(100);Lachnospiraceae uncl.(100);*
	OTU-525	0.5549	0.0074	0.4929	0.2420	*Firmicutes(100);Clostridia(100);Clostridiales(100);Ruminococcaceae(100);Ruminococcaceae uncl.(100);*
	OTU-990	0.5643	0.0060	0.4426	0.2444	*Firmicutes(100);Clostridia(100);Clostridiales(100);Ruminococcaceae(97);Ruminococcaceae uncl.(97);*
	OTU-1090	0.5621	0.0061	0.4426	0.3240	*Firmicutes(100);Clostridia(100);Clostridiales(100);Ruminococcaceae(100);Oscillibacter(67);*
	OTU-354	0.6599	0.0003	0.2608	0.3544	*Bacteroidetes(100);Bacteroidia(100);Bacteroidales(100);Porphyromonadaceae(100);Coprobacter(100);*
	OTU-309	0.5708	0.0053	0.4295	0.3954	*Firmicutes(100);Clostridia(99);Clostridiales(99);Ruminococcaceae(96);Ruminococcaceae uncl.(96);*
	OTU-2658	0.5382	0.0083	0.5376	0.4168	*Bacteria uncl.(100);*
	OTU-878	0.5660	0.0049	0.4295	0.4250	*Firmicutes(100);Clostridia(100);Clostridiales(100);Ruminococcaceae(100);Ruminococcaceae uncl.(100);*
	OTU-1740	0.5350	0.0091	0.5626	0.4465	*Firmicutes(100);Clostridia(100);Clostridiales(100);Clostridiales uncl.(100);Clostridiales uncl.(100);*
	OTU-812	0.5767	0.0032	0.3936	0.4579	*Firmicutes(100);Clostridia(100);Clostridiales(100);Ruminococcaceae(100);Ruminococcaceae uncl.(98);*
	OTU-9271	0.5240	0.0053	0.4295	0.4828	*Bacteria uncl.(100);*
	OTU-9282	0.5240	0.0054	0.4295	0.4828	*Firmicutes(100);Clostridia(100);Clostridiales(100);Ruminococcaceae(100);Ruminococcaceae uncl.(100);*
	OTU-1046	0.5496	0.0039	0.4174	0.4848	*Firmicutes(100);Clostridia(100);Clostridiales(100);Clostridiales uncl.(100);Clostridiales uncl.(100);*
	OTU-4317	0.5558	0.0068	0.4630	0.4848	*Firmicutes(100);Clostridia(100);Clostridiales(100);Clostridiales uncl.(75);Clostridiales uncl.(75);*
	OTU-2104	0.5198	0.0095	0.5742	0.4884	*Firmicutes(100);Clostridia(100);Clostridiales(100);Ruminococcaceae(100);Ruminococcaceae uncl.(100);*
	OTU-2426	0.5735	0.0041	0.4174	0.4955	*Firmicutes(100);Clostridia(100);Clostridiales(100);Ruminococcaceae(100);Ruminococcaceae uncl.(100);*
	OTU-1106	0.5329	0.0098	0.5772	0.4991	*Firmicutes(100);Clostridia(100);Clostridiales(100);Ruminococcaceae(100);Ruminococcaceae uncl.(89);*
	OTU-1423	0.5970	0.0025	0.3291	0.5023	*Firmicutes(100);Clostridia(100);Clostridiales(100);Ruminococcaceae(100);Ruminococcaceae uncl.(100);*
	OTU-1828	0.6107	0.0014	0.2608	0.5076	*Firmicutes(100);Clostridia(100);Clostridiales(100);Ruminococcaceae(100);Ruminococcaceae uncl.(67);*
	+OTU-187	0.5438	0.0055	0.4295	0.5078	*Firmicutes(100);Clostridia(100);Clostridiales(100);Lachnospiraceae(100);Lachnospiraceae uncl.(100);*
	OTU-215	0.5962	0.0036	0.4174	0.5078	*Firmicutes(100);Clostridia(100);Clostridiales(100);Ruminococcaceae(96);Ruminococcaceae uncl.(96);*
	OTU-2662	0.5766	0.0041	0.4174	0.5078	*Firmicutes(100);Clostridia(100);Clostridiales(100);Ruminococcaceae(100);Ruminococcaceae uncl.(100);*
	OTU-3085	0.5438	0.0051	0.4295	0.5078	*Firmicutes(100);Clostridia(100);Clostridiales(100);Ruminococcaceae(100);Ruminococcaceae uncl.(100);*
	OTU-4062	0.5766	0.0042	0.4174	0.5078	*Firmicutes(100);Negativicutes(100);Selenomonadales(100);Acidaminococcaceae(100);Phascolarctobacterium(100);*
	OTU-755	0.6043	0.0011	0.2608	0.5097	*Bacteroidetes(100);Bacteroidia(100);Bacteroidales(100);Porphyromonadaceae(100);Coprobacter(100);*
	OTU-3536	0.6043	0.0013	0.2608	0.5097	*Firmicutes(100);Clostridia(100);Clostridiales(100);Ruminococcaceae(100);Ruminococcaceae uncl.(100);*
	OTU-4316	0.6043	0.0013	0.2608	0.5097	*Firmicutes(100);Clostridia(100);Clostridiales(100);Ruminococcaceae(100);Ruminococcaceae uncl.(100);*
	OTU-4365	0.6043	0.0012	0.2608	0.5097	*Firmicutes(100);Clostridia(100);Clostridiales(100);Lachnospiraceae(100);Lachnospiraceae uncl.(100);*
	OTU-5978	0.6043	0.0011	0.2608	0.5097	*Firmicutes(100);Clostridia(100);Clostridiales(100);Ruminococcaceae(100);Butyricicoccus(100);*
	OTU-6195	0.6043	0.0015	0.2608	0.5097	*Firmicutes(100);Clostridia(100);Clostridiales(100);Clostridiales uncl.(100);Clostridiales uncl.(100);*
	OTU-6224	0.6043	0.0013	0.2608	0.5097	*Firmicutes(100);Clostridia(100);Clostridiales(100);Ruminococcaceae(100);Ruminococcaceae uncl.(100);*
	OTU-6452	0.6043	0.0012	0.2608	0.5097	*Firmicutes(100);Clostridia(100);Clostridiales(100);Ruminococcaceae(100);Ruminococcaceae uncl.(100);*
	OTU-6505	0.6043	0.0012	0.2608	0.5097	*Firmicutes(100);Clostridia(100);Clostridiales(100);Ruminococcaceae(100);Ruminococcaceae uncl.(100);*
	OTU-7848	0.6043	0.0013	0.2608	0.5097	*Firmicutes(100);Clostridia(100);Clostridiales(100);Ruminococcaceae(100);Oscillibacter(100);*
	OTU-1992	0.6145	0.0005	0.2608	0.5103	*Firmicutes(100);Clostridia(100);Clostridiales(100);Ruminococcaceae(100);Ruminococcaceae uncl.(100);*

	OTU-2529	0.5790	0.0015	0.2608	0.5103	*Firmicutes(100);Clostridia(100);Clostridiales(100);Ruminococcaceae(100);Ruminococcaceae uncl.(100);*
	OTU-3212	0.6139	0.0019	0.2864	0.5103	*Firmicutes(100);Clostridia(100);Clostridiales(100);Lachnospiraceae(100);Lachnospiraceae uncl.(100);*
	OTU-5781	0.6139	0.0020	0.2864	0.5103	*Firmicutes(100);Clostridia(100);Clostridiales(100);Ruminococcaceae(100);Ruminococcaceae uncl.(100);*
	OTU-2147	0.5816	0.0060	0.4426	0.5470	*Firmicutes(100);Clostridia(100);Clostridiales(100);Ruminococcaceae(100);Ruminococcaceae uncl.(100);*
	OTU-5688	0.5516	0.0068	0.4630	0.5513	*Firmicutes(100);Clostridia(100);Clostridiales(100);Ruminococcaceae(100);Ruminococcaceae uncl.(100);*
	OTU-4168	0.6028	0.0021	0.2905	0.5738	*Firmicutes(100);Clostridia(100);Clostridiales(100);Ruminococcaceae(100);Ruminococcaceae uncl.(75);*
	OTU-4914	0.6028	0.0018	0.2864	0.5738	*Firmicutes(100);Clostridia(100);Clostridiales(100);Lachnospiraceae(100);Clostridium XlVa(67);*
Calcium (mmol/l)	OTU-2502	0.6576	0.0022	1.0000	-0.6447	*Firmicutes(100);Clostridia(100);Clostridiales(100);Lachnospiraceae(100);Lachnospiraceae uncl.(86);*
	OTU-1594	0.6137	0.0059	1.0000	-0.6300	*Firmicutes(100);Clostridia(100);Clostridiales(100);Lachnospiraceae(100);Lachnospiraceae uncl.(65);*
	#OTU-2004	0.5892	0.0098	1.0000	-0.6212	*Firmicutes(100);Clostridia(100);Clostridiales(100);Lachnospiraceae(100);Lachnospiraceae uncl.(100);*
	OTU-5308	0.6119	0.0054	1.0000	-0.5478	*Firmicutes(100);Erysipelotrichia(67);Erysipelotrichales(67);Erysipelotrichaceae(67);Clostridium XVIII(67);*
	#OTU-35	0.6691	0.0017	1.0000	-0.2171	*Firmicutes(100);Clostridia(100);Clostridiales(100);Ruminococcaceae(100);Faecalibacterium(100);*
	OTU-2804	0.6255	0.0068	1.0000	0.4927	*Firmicutes(100);Clostridia(100);Clostridiales(100);Ruminococcaceae(100);Faecalibacterium(100);*
	OTU-4491	0.6255	0.0068	1.0000	0.4927	*Firmicutes(100);Clostridia(100);Clostridiales(100);Clostridiaceae 1(67);Clostridiaceae 1 uncl.(67);*
	OTU-6585	0.6551	0.0032	1.0000	0.5216	*Firmicutes(100);Clostridia(100);Clostridiales(100);Lachnospiraceae(100);Lachnospiraceae uncl.(100);*
	OTU-455	0.5904	0.0018	1.0000	0.5535	*Bacteroidetes(100);Bacteroidia(100);Bacteroidales(100);Prevotellaceae(100);Prevotella(97);*
CRP (mg/l)	OTU-209	0.6377	0.0069	0.6756	0.1059	*Bacteroidetes(100);Bacteroidia(95);Bacteroidales(95);Rikenellaceae(95);Alistipes(95);*
	†OTU-697	0.6658	0.0051	0.6756	0.218	*Firmicutes(100);Clostridia(100);Clostridiales(100);Ruminococcaceae(97);Ruminococcaceae uncl.(97);*
	OTU-958	0.5923	0.0065	0.6756	0.2574	*Firmicutes(100);Clostridia(100);Clostridiales(100);Ruminococcaceae(100);Ruminococcaceae uncl.(100);*
	OTU-2686	0.6645	0.0078	0.6756	0.2711	*Bacteroidetes(100);Bacteroidia(100);Bacteroidales(100);Rikenellaceae(100);Alistipes(100);*
	OTU-1645	0.6059	0.0083	0.6756	0.3322	*Firmicutes(100);Clostridia(100);Clostridiales(100);Clostridiales Incertae Sedis XIII(100);Clostridiales Incertae Sedis XIII uncl.(100);*
	OTU-2356	0.6264	0.0086	0.6756	0.3479	*Bacteroidetes(100);Bacteroidia(75);Bacteroidales(75);Rikenellaceae(75);Alistipes(75);*
	OTU-912	0.6700	0.0036	0.6756	0.3707	*Firmicutes(100);Clostridia(100);Clostridiales(100);Catabacteriaceae(98);Catabacter(98);*
	OTU-1287	0.6678	0.0021	0.6756	0.4309	*Firmicutes(100);Clostridia(100);Clostridiales(100);Ruminococcaceae(100);Faecalibacterium(100);*
	OTU-2843	0.6577	0.0056	0.6756	0.4323	*Firmicutes(100);Clostridia(100);Clostridiales(100);Ruminococcaceae(100);Oscillibacter(100);*
	OTU-678	0.6133	0.0097	0.6756	0.4398	*Firmicutes(100);Clostridia(100);Clostridiales(100);Ruminococcaceae(100);Oscillibacter(51);*
	OTU-2337	0.7221	0.0009	0.6756	0.4516	*Firmicutes(100);Clostridia(100);Clostridiales(100);Lachnospiraceae(100);Lachnospiraceae uncl.(88);*
	OTU-5370	0.5481	0.0089	0.6756	0.4531	*Firmicutes(100);Clostridia(100);Clostridiales(100);Ruminococcaceae(100);Clostridium IV(100);*
	OTU-7925	0.5481	0.0092	0.6756	0.4531	*Firmicutes(100);Clostridia(100);Clostridiales(100);Ruminococcaceae(100);Faecalibacterium(100);*
	OTU-550	0.5920	0.0058	0.6756	0.4560	*Firmicutes(100);Clostridia(100);Clostridiales(100);Clostridiales uncl.(99);Clostridiales uncl.(99);*
	OTU-2085	0.6137	0.0081	0.6756	0.4560	*Firmicutes(100);Clostridia(100);Clostridiales(100);Ruminococcaceae(100);Faecalibacterium(70);*
	OTU-3013	0.6112	0.0069	0.6756	0.4560	*Firmicutes(100);Clostridia(100);Clostridiales(100);Lachnospiraceae(100);Roseburia(84);*
	OTU-6049	0.5974	0.0081	0.6756	0.4560	*Bacteria uncl.(100);*
	OTU-1424	0.5951	0.0076	0.6756	0.4591	*Firmicutes(100);Clostridia(100);Clostridiales(100);Ruminococcaceae(100);Oscillibacter(53);*
	OTU-4521	0.5614	0.0057	0.6756	0.4783	*Firmicutes(100);Clostridia(100);Clostridiales(100);Lachnospiraceae(100);Roseburia(100);*
	OTU-8000	0.5614	0.0061	0.6756	0.4783	*Firmicutes(100);Clostridia(100);Clostridiales(100);Lachnospiraceae(100);Roseburia(100);*
	OTU-2803	0.6214	0.0048	0.6756	0.4800	*Firmicutes(100);Clostridia(100);Clostridiales(100);Ruminococcaceae(100);Faecalibacterium(100);*
	OTU-837	0.8147	0.0048	0.6756	0.5017	*Firmicutes(100);Clostridia(100);Clostridiales(100);Clostridiales uncl.(96);Clostridiales uncl.(96);*
	OTU-2275	0.8242	0.0043	0.6756	0.5034	*Bacteroidetes(100);Bacteroidia(100);Bacteroidales(100);Porphyromonadaceae(100);Coprobacter(100);*
	OTU-5605	0.8242	0.0041	0.6756	0.5034	*Bacteroidetes(100);Bacteroidia(100);Bacteroidales(100);Bacteroidaceae(100);Bacteroides(100);*
	OTU-7850	0.8242	0.0041	0.6756	0.5034	*Firmicutes(100);Clostridia(100);Clostridiales(100);Ruminococcaceae(100);Faecalibacterium(100);*
	OTU-7969	0.8242	0.0042	0.6756	0.5034	*Bacteroidetes(100);Bacteroidia(100);Bacteroidales(100);Rikenellaceae(100);Alistipes(100);*
	OTU-8003	0.8242	0.0042	0.6756	0.5034	*Firmicutes(100);Clostridia(100);Clostridiales(100);Lachnospiraceae(100);Dorea(100);*
	OTU-1500	0.7700	0.0006	0.6756	0.5040	*Firmicutes(100);Clostridia(100);Clostridiales(100);Ruminococcaceae(100);Ruminococcaceae uncl.(100);*
	OTU-1627	0.5951	0.0096	0.6756	0.5914	*Firmicutes(100);Clostridia(100);Clostridiales(100);Lachnospiraceae(100);Lachnospiraceae uncl.(79);*
	OTU-153	0.6129	0.0060	0.6756	0.5991	*Firmicutes(100);Clostridia(100);Clostridiales(100);Lachnospiraceae(100);Lachnospiraceae uncl.(63);*
	OTU-221	0.5818	0.0089	0.6756	0.6018	*Firmicutes(100);Clostridia(100);Clostridiales(100);Lachnospiraceae(100);Fusicatenibacter(100);*
	OTU-1093	0.7071	0.0039	0.6756	0.6840	*Firmicutes(100);Clostridia(100);Clostridiales(100);Lachnospiraceae(100);Roseburia(100);*
Eosinophiles %	OTU-1274	0.6252	0.0079	0.8147	0.3389	*Firmicutes(100);Clostridia(100);Clostridiales(100);Ruminococcaceae(100);Ruminococcaceae uncl.(100);*

	OTU-1134	0.6999	0.0027	0.6801	0.3720	*Bacteroidetes(100);Bacteroidia(100);Bacteroidales(100);Prevotellaceae(100);Prevotella(100);*
	OTU-1156	0.6306	0.0073	0.8147	0.4386	*Firmicutes(100);Clostridia(100);Clostridiales(100);Lachnospiraceae(100);Lachnospiraceae uncl.(100);*
	OTU-220	0.6634	0.0099	0.8147	0.4923	*Firmicutes(100);Bacilli(100);Bacillales(100);Bacillales Incertae Sedis XI(100);Gemella(100);*
	OTU-3774	0.6634	0.0095	0.8147	0.4923	*Firmicutes(100);Firmicutes uncl.(75);Firmicutes uncl.(75);Firmicutes uncl.(75);Firmicutes uncl.(75);*
	OTU-8701	0.6634	0.0093	0.8147	0.4923	*Firmicutes(100);Clostridia(100);Clostridiales(100);Ruminococcaceae(100);Ruminococcaceae uncl.(100);*
	OTU-1308	0.6756	0.0071	0.8147	0.5191	*Bacteroidetes(100);Bacteroidia(100);Bacteroidales(100);Prevotellaceae(100);Prevotella(100);*
	OTU-2759	0.6971	0.0038	0.6801	0.5212	*Bacteroidetes(100);Bacteroidia(100);Bacteroidales(100);Bacteroidaceae(100);Bacteroides(100);*
	OTU-5319	0.6971	0.0042	0.6801	0.5212	*Firmicutes(100);Clostridia(100);Clostridiales(100);Clostridiales uncl.(100);Clostridiales uncl.(100);*
	OTU-8634	0.6971	0.0041	0.6801	0.5212	*Firmicutes(100);Clostridia(100);Clostridiales(100);Clostridiales uncl.(100);Clostridiales uncl.(100);*
	OTU-8700	0.6971	0.0045	0.6889	0.5212	*Firmicutes(100);Clostridia(100);Clostridiales(100);Lachnospiraceae(100);Lachnospiraceae uncl.(100);*
	OTU-780	0.6736	0.0026	0.6801	0.5220	*Bacteroidetes(100);Bacteroidia(100);Bacteroidales(100);Porphyromonadaceae(100);Porphyromonas(100);*
	+OTU-865	0.6911	0.0025	0.6801	0.5220	*Proteobacteria(100);Epsilonproteobacteria(100);Campylobacterales(100);Campylobacteraceae(100);Campylobacter(100);*
	OTU-1247	0.7122	0.0022	0.6801	0.5220	*Firmicutes(100);Clostridia(100);Clostridiales(100);Clostridiales Incertae Sedis XI(100);Murdochiella(100);*
	OTU-4456	0.6641	0.0053	0.7571	0.5231	*Firmicutes(100);Clostridia(100);Clostridiales(100);Clostridiales uncl.(100);Clostridiales uncl.(100);*
	OTU-1668	0.6481	0.0028	0.6801	0.5254	*Firmicutes(100);Clostridia(100);Clostridiales(100);Ruminococcaceae(100);Faecalibacterium(100);*
	OTU-686	0.7170	0.0016	0.6801	0.5357	*Firmicutes(100);Clostridia(100);Clostridiales(100);Lachnospiraceae(100);Blautia(100);*
	OTU-3565	0.7023	0.0040	0.6801	0.5596	*Firmicutes(100);Clostridia(100);Clostridiales(100);Ruminococcaceae(80);Ruminococcaceae uncl.(80);*
	OTU-860	0.6966	0.0038	0.6801	0.5622	***Proteobacteria(100);Gammaproteobacteria(100);Pasteurellales(100);Pasteurellaceae(100);Haemophilus(100);***
	OTU-3219	0.6664	0.0035	0.6801	0.5864	*Bacteroidetes(100);Bacteroidia(100);Bacteroidales(100);Bacteroidaceae(100);Bacteroides(100);*
	OTU-4298	0.7564	0.0011	0.6801	0.5877	*Firmicutes(100);Clostridia(100);Clostridiales(100);Lachnospiraceae(100);Lachnospiraceae uncl.(100);*
	OTU-1602	0.6681	0.0040	0.6801	0.6022	*Firmicutes(100);Clostridia(100);Clostridiales(100);Lachnospiraceae(100);Lachnospiraceae uncl.(100);*
	OTU-1165	0.7284	0.0007	0.6801	0.6198	*Firmicutes(100);Clostridia(100);Clostridiales(100);Lachnospiraceae(100);Lachnospiraceae uncl.(100);*
	OTU-856	0.6741	0.0002	0.5959	0.6222	*Firmicutes(100);Clostridia(100);Clostridiales(100);Clostridiales Incertae Sedis XI(100);Ezakiella(100);*
Erythrocytes (cells/pl)	OTU-3332	0.7921	0.0001	0.1814	-0.7097	*Firmicutes(100);Clostridia(100);Clostridiales(100);Ruminococcaceae(100);Faecalibacterium(100);*
	OTU-1608	0.7049	0.0002	0.2246	-0.5966	*Bacteria uncl.(79);*
	OTU-1988	0.6885	0.0005	0.3310	-0.5952	*Firmicutes(100);Clostridia(100);Clostridiales(100);Clostridiales uncl.(100);Clostridiales uncl.(100);*
	OTU-1744	0.6708	0.0003	0.2246	-0.5767	*Firmicutes(100);Clostridia(100);Clostridiales(100);Ruminococcaceae(100);Oscillibacter(93);*
	OTU-2084	0.6324	0.0021	0.3310	-0.5654	*Firmicutes(100);Clostridia(100);Clostridiales(100);Clostridiales Incertae Sedis XIII(90);Clostridiales Incertae Sedis XIII uncl.(90);*
	OTU-2304	0.5800	0.0058	0.4189	-0.5389	*Firmicutes(100);Clostridia(100);Clostridiales(100);Ruminococcaceae(100);Ruminococcaceae uncl.(100);*
	OTU-3190	0.5642	0.0096	0.5766	-0.5057	*Bacteroidetes(100);Bacteroidia(100);Bacteroidales(100);Porphyromonadaceae(100);Odoribacter(100);*
	OTU-550	0.5394	0.0013	0.3310	-0.4992	*Firmicutes(100);Clostridia(100);Clostridiales(100);Clostridiales uncl.(99);Clostridiales uncl.(99);*
	OTU-2085	0.6121	0.0013	0.3310	-0.4992	*Firmicutes(100);Clostridia(100);Clostridiales(100);Ruminococcaceae(100);Faecalibacterium(70);*
	OTU-3013	0.6242	0.0016	0.3310	-0.4992	*Firmicutes(100);Clostridia(100);Clostridiales(100);Lachnospiraceae(100);Roseburia(84);*
	OTU-6049	0.6346	0.0012	0.3310	-0.4992	*Bacteria uncl.(100);*
	OTU-2019	0.6221	0.0034	0.3310	-0.4986	*Firmicutes(100);Clostridia(100);Clostridiales(100);Ruminococcaceae(100);Ruminococcaceae uncl.(100);*
	OTU-3830	0.6221	0.0036	0.3310	-0.4986	*Firmicutes(100);Clostridia(100);Clostridiales(100);Clostridiales uncl.(100);Clostridiales uncl.(100);*
	OTU-4671	0.6221	0.0035	0.3310	-0.4986	*Firmicutes(100);Clostridia(100);Clostridiales(100);Ruminococcaceae(100);Faecalibacterium(100);*
	OTU-4732	0.6221	0.0037	0.3310	-0.4986	*Firmicutes(100);Clostridia(100);Clostridiales(100);Clostridiales uncl.(67);Clostridiales uncl.(67);*
	OTU-4800	0.6221	0.0034	0.3310	-0.4986	*Firmicutes(100);Clostridia(100);Clostridiales(100);Ruminococcaceae(100);Ruminococcaceae uncl.(100);*
	OTU-5344	0.6221	0.0037	0.3310	-0.4986	*Firmicutes(100);Clostridia(100);Clostridiales(100);Clostridiales uncl.(100);Clostridiales uncl.(100);*
	OTU-5492	0.6221	0.0035	0.3310	-0.4986	*Firmicutes(100);Clostridia(100);Clostridiales(100);Lachnospiraceae(100);Lachnospiraceae uncl.(100);*
	OTU-5758	0.6221	0.0039	0.3310	-0.4986	*Firmicutes(100);Clostridia(100);Clostridiales(100);Ruminococcaceae(100);Ruminococcaceae uncl.(100);*
	OTU-7644	0.6221	0.0038	0.3310	-0.4986	*Firmicutes(100);Clostridia(100);Clostridiales(100);Lachnospiraceae(100);Anaerostipes(100);*
	OTU-7773	0.6221	0.0035	0.3310	-0.4986	*Firmicutes(100);Clostridia(100);Clostridiales(100);Clostridiales uncl.(100);Clostridiales uncl.(100);*
	OTU-7790	0.6221	0.0036	0.3310	-0.4986	*Firmicutes(100);Clostridia(100);Clostridiales(100);Clostridiales uncl.(100);Clostridiales uncl.(100);*
	OTU-7840	0.6221	0.0035	0.3310	-0.4986	*Firmicutes(100);Clostridia(100);Clostridiales(100);Lachnospiraceae(100);Lachnospiraceae uncl.(100);*
	OTU-7980	0.6221	0.0037	0.3310	-0.4986	*Firmicutes(100);Clostridia(100);Clostridiales(100);Clostridiales uncl.(100);Clostridiales uncl.(100);*
	OTU-7981	0.6221	0.0031	0.3310	-0.4986	*Firmicutes(100);Clostridia(100);Clostridiales(100);Ruminococcaceae(100);Ruminococcus(100);*
	OTU-7993	0.6221	0.0034	0.3310	-0.4986	*Bacteria uncl.(100);*
	OTU-9136	0.6221	0.0037	0.3310	-0.4986	*Bacteria uncl.(100);*

	OTU-9302	0.6221	0.0034	0.3310	-0.4986	*Firmicutes(100);Clostridia(100);Clostridiales(100);Ruminococcaceae(100);Faecalibacterium(100);*
	OTU-3169	0.5929	0.0076	0.4822	-0.4969	*Bacteria uncl.(61);*
	OTU-894	0.6078	0.0017	0.3310	-0.4771	*Firmicutes(100);Clostridia(100);Clostridiales(100);Ruminococcaceae(95);Oscillibacter(78);*
	OTU-2482	0.5943	0.0027	0.3310	-0.4754	*Firmicutes(100);Clostridia(100);Clostridiales(100);Ruminococcaceae(100);Ruminococcaceae uncl.(100);*
	OTU-4333	0.5943	0.0027	0.3310	-0.4754	*Bacteroidetes(100);Bacteroidia(100);Bacteroidales(100);Rikenellaceae(100);Alistipes(100);*
	OTU-5543	0.5943	0.0031	0.3310	-0.4754	*Firmicutes(100);Clostridia(100);Clostridiales(100);Ruminococcaceae(100);Faecalibacterium(67);*
	OTU-2553	0.5748	0.0062	0.4192	-0.4516	*Firmicutes(100);Clostridia(100);Clostridiales(100);Ruminococcaceae(100);Oscillibacter(58);*
	OTU-750	0.5730	0.0054	0.4189	-0.4249	*Firmicutes(100);Clostridia(97);Clostridiales(97);Clostridiales uncl.(97);Clostridiales uncl.(97);*
	OTU-1363	0.5716	0.0084	0.5170	-0.3998	*Firmicutes(100);Clostridia(100);Clostridiales(100);Lachnospiraceae(100);Fusicatenibacter(100);*
	OTU-912	0.5811	0.0053	0.4189	-0.3902	*Firmicutes(100);Clostridia(100);Clostridiales(100);Catabacteriaceae(98);Catabacter(98);*
	OTU-190	0.5915	0.0061	0.4192	-0.3818	*Firmicutes(100);Clostridia(100);Clostridiales(100);Ruminococcaceae(100);Ruminococcaceae uncl.(100);*
	OTU-3515	0.5878	0.0057	0.4189	0.4986	*Firmicutes(100);Clostridia(100);Clostridiales(100);Lachnospiraceae(100);Lachnospiraceae uncl.(100);*
	OTU-3863	0.5878	0.0055	0.4189	0.4986	*Firmicutes(100);Clostridia(100);Clostridiales(100);Lachnospiraceae(100);Roseburia(100);*
	‡OTU-2020	0.5751	0.0076	0.4822	0.5415	*Bacteroidetes(100);Bacteroidia(100);Bacteroidales(100);Bacteroidaceae(100);Bacteroides(100);*
	‡OTU-2811	0.5759	0.0063	0.4192	0.5909	*Firmicutes(100);Clostridia(100);Clostridiales(100);Lachnospiraceae(100);Lachnospiraceae uncl.(100);*
	OTU-593	0.6158	0.0040	0.3310	0.6426	*Bacteroidetes(100);Bacteroidia(100);Bacteroidales(100);Bacteroidaceae(100);Bacteroides(100);*
Hb (g/dl)	OTU-678	0.6650	0.0021	0.7154	-0.6462	*Firmicutes(100);Clostridia(100);Clostridiales(100);Ruminococcaceae(100);Oscillibacter(51);*
	OTU-2843	0.6613	0.0015	0.7154	-0.5540	*Firmicutes(100);Clostridia(100);Clostridiales(100);Ruminococcaceae(100);Oscillibacter(100);*
	OTU-2969	0.5654	0.0079	0.7154	-0.5209	*Firmicutes(100);Clostridia(100);Clostridiales(100);Ruminococcaceae(100);Faecalibacterium(100);*
	OTU-688	0.6598	0.0039	0.7154	-0.5093	*Firmicutes(100);Clostridia(100);Clostridiales(100);Clostridiales Incertae Sedis XIII(97);Clostridiales Incertae Sedis XIII uncl.(94);*
	OTU-2084	0.6530	0.0029	0.7154	-0.5042	*Firmicutes(100);Clostridia(100);Clostridiales(100);Clostridiales Incertae Sedis XIII(90);Clostridiales Incertae Sedis XIII uncl.(90);*
	OTU-1327	0.6881	0.0044	0.7154	-0.5040	*Firmicutes(100);Clostridia(100);Clostridiales(100);Ruminococcaceae(100);Oscillibacter(100);*
	OTU-2553	0.7107	0.0006	0.7154	-0.4997	*Firmicutes(100);Clostridia(100);Clostridiales(100);Ruminococcaceae(100);Oscillibacter(58);*
	OTU-2672	0.6884	0.0018	0.7154	-0.4991	*Firmicutes(100);Clostridia(100);Clostridiales(100);Ruminococcaceae(100);Ruminococcaceae uncl.(72);*
	OTU-3785	0.6884	0.0015	0.7154	-0.4991	*Firmicutes(100);Clostridia(100);Clostridiales(100);Lachnospiraceae(100);Lachnospiraceae uncl.(100);*
	OTU-6395	0.6884	0.0016	0.7154	-0.4991	*Firmicutes(100);Clostridia(100);Clostridiales(100);Lachnospiraceae(100);Lachnospiraceae uncl.(100);*
	OTU-3324	0.7023	0.0044	0.7154	-0.4759	*Bacteroidetes(100);Bacteroidetes uncl.(61);Bacteroidetes uncl.(61);Bacteroidetes uncl.(61);Bacteroidetes uncl.(61);*
	OTU-4886	0.7139	0.0036	0.7154	-0.4759	*Firmicutes(100);Clostridia(100);Clostridiales(100);Ruminococcaceae(100);Ruminococcaceae uncl.(100);*
	OTU-1802	0.6737	0.0062	0.7154	-0.4742	*Firmicutes(100);Clostridia(100);Clostridiales(100);Ruminococcaceae(100);Ruminococcaceae uncl.(100);*
	OTU-3680	0.6737	0.0065	0.7154	-0.4742	*Firmicutes(100);Clostridia(100);Clostridiales(100);Ruminococcaceae(75);Ruminococcaceae uncl.(75);*
	OTU-3955	0.6737	0.0068	0.7154	-0.4742	*Firmicutes(100);Clostridia(100);Clostridiales(100);Ruminococcaceae(100);Faecalibacterium(100);*
	OTU-4568	0.6737	0.0061	0.7154	-0.4742	*Firmicutes(100);Clostridia(100);Clostridiales(100);Ruminococcaceae(100);Ruminococcaceae uncl.(67);*
	OTU-6824	0.6737	0.0065	0.7154	-0.4742	*Firmicutes(100);Erysipelotrichia(100);Erysipelotrichales(100);Erysipelotrichaceae(100);Holdemania(100);*
	OTU-7833	0.6737	0.0066	0.7154	-0.4742	*Firmicutes(100);Clostridia(100);Clostridiales(100);Ruminococcaceae(100);Faecalibacterium(100);*
	OTU-7844	0.6737	0.0061	0.7154	-0.4742	*Firmicutes(100);Clostridia(100);Clostridiales(100);Ruminococcaceae(100);Ruminococcaceae uncl.(100);*
	OTU-1457	0.6162	0.0050	0.7154	-0.4543	*Firmicutes(100);Clostridia(100);Clostridiales(100);Ruminococcaceae(100);Faecalibacterium(100);*
	OTU-1500	0.6629	0.0053	0.7154	-0.4521	*Firmicutes(100);Clostridia(100);Clostridiales(100);Ruminococcaceae(100);Ruminococcaceae uncl.(100);*
	OTU-1744	0.6754	0.0038	0.7154	-0.448	*Firmicutes(100);Clostridia(100);Clostridiales(100);Ruminococcaceae(100);Oscillibacter(93);*
	OTU-1694	0.6106	0.0059	0.7154	-0.4379	*Firmicutes(100);Clostridia(100);Clostridiales(100);Lachnospiraceae(100);Lachnospiraceae uncl.(100);*
	OTU-750	0.6690	0.0072	0.7154	-0.4377	*Firmicutes(100);Clostridia(97);Clostridiales(97);Clostridiales uncl.(97);Clostridiales uncl.(97);*
	OTU-874	0.6301	0.0091	0.7154	-0.3926	*Bacteria uncl.(84);*
	†OTU-697	0.6349	0.0034	0.7154	-0.3553	*Firmicutes(100);Clostridia(100);Clostridiales(100);Ruminococcaceae(97);Ruminococcaceae uncl.(97);*
	OTU-948	0.6244	0.0095	0.7154	-0.1391	*Firmicutes(100);Clostridia(100);Clostridiales(100);Ruminococcaceae(100);Faecalibacterium(100);*
	OTU-744	0.6653	0.0018	0.7154	0.0234	*Firmicutes(100);Clostridia(100);Clostridiales(100);Ruminococcaceae(100);Faecalibacterium(100);*
Hematocrit	OTU-134	0.5994	0.0098	0.5633	-0.6347	*Firmicutes(100);Clostridia(100);Clostridiales(100);Ruminococcaceae(100);Oscillibacter(92);*
	OTU-3332	0.5979	0.0038	0.5633	-0.6137	*Firmicutes(100);Clostridia(100);Clostridiales(100);Ruminococcaceae(100);Faecalibacterium(100);*
	OTU-678	0.5819	0.0063	0.5633	-0.5997	*Firmicutes(100);Clostridia(100);Clostridiales(100);Ruminococcaceae(100);Oscillibacter(51);*
	OTU-2969	0.5817	0.0041	0.5633	-0.5635	*Firmicutes(100);Clostridia(100);Clostridiales(100);Ruminococcaceae(100);Faecalibacterium(100);*

	OTU-1744	0.6775	0.0002	0.5633	-0.5629	*Firmicutes(100);Clostridia(100);Clostridiales(100);Ruminococcaceae(100);Oscillibacter(93);*
	OTU-1457	0.6107	0.0023	0.5633	-0.5607	*Firmicutes(100);Clostridia(100);Clostridiales(100);Ruminococcaceae(100);Faecalibacterium(100);*
	†OTU-697	0.6016	0.0041	0.5633	-0.5519	*Firmicutes(100);Clostridia(100);Clostridiales(100);Ruminococcaceae(97);Ruminococcaceae uncl.(97);*
	OTU-912	0.6832	0.0006	0.5633	-0.5391	*Firmicutes(100);Clostridia(100);Clostridiales(100);Catabacteriaceae(98);Catabacter(98);*
	OTU-2553	0.6620	0.0020	0.5633	-0.5046	*Firmicutes(100);Clostridia(100);Clostridiales(100);Ruminococcaceae(100);Oscillibacter(58);*
	OTU-2672	0.5966	0.0065	0.5633	-0.504	*Firmicutes(100);Clostridia(100);Clostridiales(100);Ruminococcaceae(100);Ruminococcaceae uncl.(72);*
	OTU-3785	0.5966	0.0061	0.5633	-0.504	*Firmicutes(100);Clostridia(100);Clostridiales(100);Lachnospiraceae(100);Lachnospiraceae uncl.(100);*
	OTU-6395	0.5966	0.0063	0.5633	-0.504	*Firmicutes(100);Clostridia(100);Clostridiales(100);Lachnospiraceae(100);Lachnospiraceae uncl.(100);*
	OTU-1608	0.6127	0.0039	0.5633	-0.5026	*Bacteria uncl.(79);*
	OTU-750	0.6572	0.0034	0.5633	-0.5022	*Firmicutes(100);Clostridia(97);Clostridiales(97);Clostridiales uncl.(97);Clostridiales uncl.(97);*
	OTU-1327	0.6512	0.0030	0.5633	-0.5022	*Firmicutes(100);Clostridia(100);Clostridiales(100);Ruminococcaceae(100);Oscillibacter(100);*
	OTU-894	0.6621	0.0014	0.5633	-0.5002	*Firmicutes(100);Clostridia(100);Clostridiales(100);Ruminococcaceae(95);Oscillibacter(78);*
	OTU-2084	0.5956	0.0061	0.5633	-0.4466	*Firmicutes(100);Clostridia(100);Clostridiales(100);Clostridiales Incertae Sedis XIII(90);Clostridiales Incertae Sedis XIII uncl.(90);*
	OTU-550	0.6329	0.0062	0.5633	-0.4325	*Firmicutes(100);Clostridia(100);Clostridiales(100);Clostridiales uncl.(99);Clostridiales uncl.(99);*
	OTU-1500	0.6301	0.0066	0.5633	-0.4325	*Firmicutes(100);Clostridia(100);Clostridiales(100);Ruminococcaceae(100);Ruminococcaceae uncl.(100);*
	OTU-2085	0.6381	0.0082	0.5633	-0.4325	*Firmicutes(100);Clostridia(100);Clostridiales(100);Ruminococcaceae(100);Faecalibacterium(70);*
	OTU-3013	0.6301	0.0061	0.5633	-0.4325	*Firmicutes(100);Clostridia(100);Clostridiales(100);Lachnospiraceae(100);Roseburia(84);*
	OTU-190	0.6075	0.0034	0.5633	-0.4325	*Firmicutes(100);Clostridia(100);Clostridiales(100);Ruminococcaceae(100);Ruminococcaceae uncl.(100);*
	OTU-2695	0.6276	0.0060	0.5633	-0.4222	*Bacteria uncl.(100);*
	OTU-3373	0.6089	0.0069	0.5633	-0.4222	*Firmicutes(100);Clostridia(100);Clostridiales(100);Lachnospiraceae(100);Blautia(61);*
	OTU-699	0.6389	0.0098	0.5633	-0.3683	*Firmicutes(100);Clostridia(100);Clostridiales(100);Clostridiales uncl.(100);Clostridiales uncl.(100);*
	OTU-2981	0.5442	0.0081	0.5633	0.5278	*Firmicutes(100);Clostridia(100);Clostridiales(100);Lachnospiraceae(100);Roseburia(100);*
	‡OTU-2811	0.6577	0.0013	0.5633	0.6031	*Firmicutes(100);Clostridia(100);Clostridiales(100);Lachnospiraceae(100);Lachnospiraceae uncl.(100);*
	OTU-593	0.6321	0.0022	0.5633	0.6765	*Bacteroidetes(100);Bacteroidia(100);Bacteroidales(100);Bacteroidaceae(100);Bacteroides(100);*
	OTU-2	0.6605	0.0012	0.5633	0.6848	*Bacteroidetes(100);Bacteroidia(100);Bacteroidales(100);Bacteroidaceae(100);Bacteroides(100);*
LDH (U/l)	OTU-192	0.5963	0.0072	0.9512	-0.0137	*Firmicutes(100);Clostridia(100);Clostridiales(100);Lachnospiraceae(100);Clostridium XlVa(98);*
	OTU-1713	0.6035	0.0086	0.9512	0.2953	*Firmicutes(100);Clostridia(100);Clostridiales(100);Lachnospiraceae(100);Lachnospiraceae uncl.(100);*
	OTU-5965	0.6358	0.0088	0.9512	0.4917	*Firmicutes(100);Clostridia(100);Clostridiales(100);Ruminococcaceae(100);Faecalibacterium(100);*
	OTU-6787	0.6358	0.0084	0.9512	0.4917	*Firmicutes(100);Clostridia(100);Clostridiales(100);Lachnospiraceae(100);Lachnospiraceae uncl.(100);*
	OTU-7580	0.6358	0.0095	0.9512	0.4917	*Firmicutes(100);Clostridia(100);Clostridiales(100);Lachnospiraceae(100);Clostridium XlVa(100);*
	OTU-1890	0.5921	0.0074	0.9512	0.5185	*Firmicutes(100);Clostridia(100);Clostridiales(100);Lachnospiraceae(100);Blautia(91);*
	OTU-2392	0.6558	0.0031	0.9512	0.5207	*Firmicutes(100);Clostridia(100);Clostridiales(100);Lachnospiraceae(100);Lachnospiraceae uncl.(100);*
	OTU-5136	0.6558	0.0028	0.9512	0.5207	*Bacteroidetes(100);Bacteroidia(100);Bacteroidales(100);Bacteroidaceae(100);Bacteroides(100);*
	OTU-5487	0.6558	0.0030	0.9512	0.5207	*Firmicutes(100);Clostridia(100);Clostridiales(100);Lachnospiraceae(100);Lachnospiraceae uncl.(100);*
	OTU-9281	0.6558	0.0030	0.9512	0.5207	*Firmicutes(100);Clostridia(100);Clostridiales(100);Lachnospiraceae(100);Lachnospiraceae uncl.(100);*
Lymphocytes %	OTU-3005	0.6576	0.0025	0.6633	-0.6354	*Firmicutes(100);Clostridia(100);Clostridiales(100);Ruminococcaceae(100);Oscillibacter(84);*
	OTU-1969	0.6961	0.0020	0.6633	-0.6211	*Firmicutes(100);Clostridia(100);Clostridiales(100);Ruminococcaceae(100);Ruminococcaceae uncl.(90);*
	OTU-2658	0.6987	0.0007	0.6633	-0.6211	*Bacteria uncl.(100);*
	OTU-2998	0.7037	0.0010	0.6633	-0.6211	*Firmicutes(100);Clostridia(100);Clostridiales(100);Ruminococcaceae(100);Faecalibacterium(84);*
	+OTU-328	0.6195	0.0072	0.7175	-0.6205	*Proteobacteria(100);Deltaproteobacteria(100);Desulfovibrionales(100);Desulfovibrionaceae(100);Bilophila(100);*
	OTU-3790	0.6821	0.0011	0.6633	-0.6192	*Firmicutes(100);Clostridia(100);Clostridiales(100);Ruminococcaceae(100);Faecalibacterium(100);*
	OTU-884	0.6691	0.0049	0.7175	-0.5781	*Firmicutes(100);Clostridia(100);Clostridiales(100);Ruminococcaceae(100);Flavonifractor(96);*
	OTU-1238	0.6113	0.0074	0.7175	-0.5772	*Firmicutes(100);Clostridia(100);Clostridiales(100);Ruminococcaceae(100);Faecalibacterium(96);*
	OTU-1775	0.5924	0.0025	0.6633	-0.521	*Firmicutes(100);Clostridia(100);Clostridiales(100);Ruminococcaceae(100);Ruminococcaceae uncl.(75);*
	OTU-4176	0.6389	0.0058	0.7175	-0.521	*Firmicutes(75);Firmicutes uncl.(75);Firmicutes uncl.(75);Firmicutes uncl.(75);Firmicutes uncl.(75);*
	OTU-1606	0.6337	0.0088	0.7175	-0.5203	*Firmicutes(100);Clostridia(100);Clostridiales(100);Ruminococcaceae(100);Ruminococcaceae uncl.(79);*
	OTU-2126	0.6337	0.0089	0.7175	-0.5203	*Firmicutes(89);Firmicutes uncl.(56);Firmicutes uncl.(56);Firmicutes uncl.(56);Firmicutes uncl.(56);*
	OTU-3889	0.6337	0.0092	0.7175	-0.5203	*Firmicutes(100);Clostridia(100);Clostridiales(100);Ruminococcaceae(100);Faecalibacterium(75);*
	OTU-5969	0.6337	0.0087	0.7175	-0.5203	***Proteobacteria(67);Gammaproteobacteria(67);Pasteurellales(67);Pasteurellaceae(67);Pasteurellaceae uncl.(67);***

	OTU-6204	0.6337	0.0083	0.7175	-0.5203	*Bacteria uncl.(100);*
	OTU-7133	0.6337	0.0076	0.7175	-0.5203	*Bacteria uncl.(100);*
	OTU-7762	0.6337	0.0087	0.7175	-0.5203	*Firmicutes(100);Clostridia(100);Clostridiales(100);Clostridiales uncl.(100);Clostridiales uncl.(100);*
	OTU-7792	0.6337	0.0089	0.7175	-0.5203	*Firmicutes(100);Clostridia(100);Clostridiales(100);Ruminococcaceae(100);Oscillibacter(100);*
	OTU-712	0.6310	0.0094	0.7175	-0.5181	*Firmicutes(94);Clostridia(58);Clostridia uncl.(58);Clostridia uncl.(58);Clostridia uncl.(58);*
	OTU-2529	0.5896	0.0088	0.7175	-0.4936	*Firmicutes(100);Clostridia(100);Clostridiales(100);Ruminococcaceae(100);Ruminococcaceae uncl.(100);*
	OTU-345	0.6260	0.0089	0.7175	-0.4523	*Firmicutes(100);Clostridia(100);Clostridiales(100);Lachnospiraceae(100);Lachnospiraceae uncl.(83);*
	OTU-442	0.6256	0.0085	0.7175	-0.402	*Firmicutes(100);Clostridia(100);Clostridiales(100);Ruminococcaceae(99);Ruminococcaceae uncl.(98);*
	OTU-106	0.6464	0.0043	0.7175	0.2466	*Bacteroidetes(100);Bacteroidia(100);Bacteroidales(100);Rikenellaceae(100);Alistipes(100);*
	OTU-6435	0.7083	0.0039	0.7175	0.5203	*Firmicutes(100);Clostridia(100);Clostridiales(100);Ruminococcaceae(100);Ruminococcaceae uncl.(100);*
	OTU-1187	0.5968	0.0026	0.6633	0.521	*Firmicutes(100);Clostridia(100);Clostridiales(100);Lachnospiraceae(96);Clostridium XlVb(92);*
	OTU-4614	0.6964	0.0019	0.6633	0.521	*Firmicutes(100);Clostridia(100);Clostridiales(100);Lachnospiraceae(100);Lachnospiraceae uncl.(100);*
	OTU-2266	0.6708	0.0014	0.6633	0.5751	*Firmicutes(100);Clostridia(100);Clostridiales(100);Ruminococcaceae(100);Ruminococcaceae uncl.(100);*
	OTU-1278	0.7310	0.0003	0.6633	0.5806	*Firmicutes(100);Clostridia(100);Clostridiales(100);Lachnospiraceae(100);Clostridium XlVa(100);*
MCH (pg/cell)	OTU-1327	0.6158	0.0017	0.6659	-0.5752	*Firmicutes(100);Clostridia(100);Clostridiales(100);Ruminococcaceae(100);Oscillibacter(100);*
	OTU-1256	0.5586	0.0095	0.6991	-0.5638	*Firmicutes(100);Clostridia(100);Clostridiales(100);Ruminococcaceae(96);Ruminococcaceae uncl.(96);*
	OTU-2843	0.5549	0.0086	0.6991	-0.5515	*Firmicutes(100);Clostridia(100);Clostridiales(100);Ruminococcaceae(100);Oscillibacter(100);*
	OTU-1887	0.5855	0.0062	0.6659	-0.5482	*Firmicutes(100);Clostridia(100);Clostridiales(100);Lachnospiraceae(100);Eisenbergiella(100);*
	OTU-688	0.6008	0.0033	0.6659	-0.546	*Firmicutes(100);Clostridia(100);Clostridiales(100);Clostridiales Incertae Sedis XIII(97);Clostridiales Incertae Sedis XIII uncl.(94);*
	OTU-678	0.5777	0.0077	0.6659	-0.5111	*Firmicutes(100);Clostridia(100);Clostridiales(100);Ruminococcaceae(100);Oscillibacter(51);*
	OTU-2553	0.6006	0.0017	0.6659	-0.4977	*Firmicutes(100);Clostridia(100);Clostridiales(100);Ruminococcaceae(100);Oscillibacter(58);*
	OTU-3324	0.5902	0.0034	0.6659	-0.4977	*Bacteroidetes(100);Bacteroidetes uncl.(61);Bacteroidetes uncl.(61);Bacteroidetes uncl.(61);Bacteroidetes uncl.(61);*
	OTU-4886	0.6022	0.0027	0.6659	-0.4977	*Firmicutes(100);Clostridia(100);Clostridiales(100);Ruminococcaceae(100);Ruminococcaceae uncl.(100);*
	OTU-1802	0.5629	0.0062	0.6659	-0.4964	*Firmicutes(100);Clostridia(100);Clostridiales(100);Ruminococcaceae(100);Ruminococcaceae uncl.(100);*
	OTU-2672	0.5629	0.0061	0.6659	-0.4964	*Firmicutes(100);Clostridia(100);Clostridiales(100);Ruminococcaceae(100);Ruminococcaceae uncl.(72);*
	OTU-3680	0.5629	0.0063	0.6659	-0.4964	*Firmicutes(100);Clostridia(100);Clostridiales(100);Ruminococcaceae(75);Ruminococcaceae uncl.(75);*
	OTU-3785	0.5629	0.0061	0.6659	-0.4964	*Firmicutes(100);Clostridia(100);Clostridiales(100);Lachnospiraceae(100);Lachnospiraceae uncl.(100);*
	OTU-3955	0.5629	0.0059	0.6659	-0.4964	*Firmicutes(100);Clostridia(100);Clostridiales(100);Ruminococcaceae(100);Faecalibacterium(100);*
	OTU-4568	0.5629	0.0062	0.6659	-0.4964	*Firmicutes(100);Clostridia(100);Clostridiales(100);Ruminococcaceae(100);Ruminococcaceae uncl.(67);*
	OTU-6395	0.5629	0.0059	0.6659	-0.4964	*Firmicutes(100);Clostridia(100);Clostridiales(100);Lachnospiraceae(100);Lachnospiraceae uncl.(100);*
	OTU-6824	0.5629	0.0062	0.6659	-0.4964	*Firmicutes(100);Erysipelotrichia(100);Erysipelotrichales(100);Erysipelotrichaceae(100);Holdemania(100);*
	OTU-7833	0.5629	0.0070	0.6659	-0.4964	*Firmicutes(100);Clostridia(100);Clostridiales(100);Ruminococcaceae(100);Faecalibacterium(100);*
	OTU-7844	0.5629	0.0064	0.6659	-0.4964	*Firmicutes(100);Clostridia(100);Clostridiales(100);Ruminococcaceae(100);Ruminococcaceae uncl.(100);*
	OTU-804	0.5660	0.0071	0.6659	-0.4594	*Bacteroidetes(100);Bacteroidia(100);Bacteroidales(100);Bacteroidaceae(100);Bacteroides(100);*
	OTU-874	0.5680	0.0061	0.6659	-0.4594	*Bacteria uncl.(84);*
	OTU-5530	0.5571	0.0081	0.6770	-0.4594	*Firmicutes(100);Clostridia(100);Clostridiales(100);Clostridiales uncl.(67);Clostridiales uncl.(67);*
	OTU-1500	0.5684	0.0075	0.6659	-0.4339	*Firmicutes(100);Clostridia(100);Clostridiales(100);Ruminococcaceae(100);Ruminococcaceae uncl.(100);*
	+OTU-123	0.5952	0.0075	0.6659	0.3283	*Firmicutes(100);Clostridia(100);Clostridiales(100);Ruminococcaceae(100);Oscillibacter(90);*
	OTU-38	0.5804	0.0077	0.6659	0.5023	***Proteobacteria(100);Gammaproteobacteria(100);Pasteurellales(100);Pasteurellaceae(100);Haemophilus(82);***
	OTU-3120	0.5723	0.0042	0.6659	0.5098	*Firmicutes(100);Clostridia(100);Clostridiales(100);Lachnospiraceae(100);Lachnospiraceae uncl.(100);*
	OTU-4433	0.5723	0.0038	0.6659	0.5098	*Bacteroidetes(100);Bacteroidia(100);Bacteroidales(100);Bacteroidaceae(100);Bacteroides(100);*
	OTU-5690	0.5723	0.0042	0.6659	0.5098	*Firmicutes(100);Clostridia(100);Clostridiales(100);Lachnospiraceae(100);Lachnospiraceae uncl.(100);*
	OTU-6598	0.5723	0.0042	0.6659	0.5098	*Firmicutes(100);Clostridia(100);Clostridiales(100);Lachnospiraceae(100);Lachnospiraceae uncl.(100);*
	OTU-7642	0.5723	0.0042	0.6659	0.5098	*Firmicutes(100);Clostridia(100);Clostridiales(100);Ruminococcaceae(100);Oscillibacter(100);*
	OTU-5292	0.5622	0.0074	0.6659	0.529	*Firmicutes(100);Clostridia(100);Clostridiales(100);Lachnospiraceae(100);Lachnospiraceae uncl.(67);*
	OTU-1606	0.5728	0.0048	0.6659	0.5515	*Firmicutes(100);Clostridia(100);Clostridiales(100);Ruminococcaceae(100);Ruminococcaceae uncl.(79);*
	OTU-987	0.6692	0.0006	0.6659	0.6035	*Firmicutes(100);Clostridia(100);Clostridiales(100);Lachnospiraceae(100);Lachnospiraceae uncl.(100);*
MCHC (g/dl)	OTU-1887	0.6636	0.0020	0.5491	-0.6021	*Firmicutes(100);Clostridia(100);Clostridiales(100);Lachnospiraceae(100);Eisenbergiella(100);*
	OTU-829	0.6639	0.0013	0.5491	-0.5915	*Bacteroidetes(100);Bacteroidia(100);Bacteroidales(100);Bacteroidaceae(100);Bacteroides(100);*

	OTU-397	0.5185	0.0053	0.5491	-0.5172	*Firmicutes(100);Clostridia(100);Clostridiales(100);Lachnospiraceae(100);Lachnospiraceae uncl.(85);*
	OTU-688	0.5994	0.0045	0.5491	-0.5096	*Firmicutes(100);Clostridia(100);Clostridiales(100);Clostridiales Incertae Sedis XIII(97);Clostridiales Incertae Sedis XIII uncl.(94);*
	OTU-80	0.4501	0.0049	0.5491	0.317	*Firmicutes(99);Clostridia(98);Clostridiales(98);Clostridiales uncl.(98);Clostridiales uncl.(98);*
	OTU-243	0.5486	0.0088	0.6298	0.3739	*Bacteria uncl.(94);*
	OTU-1743	0.5550	0.0077	0.6251	0.4596	*Firmicutes(100);Clostridia(100);Clostridiales(100);Ruminococcaceae(100);Faecalibacterium(100);*
	OTU-1702	0.5173	0.0058	0.5519	0.494	*Firmicutes(54);Clostridia(54);Clostridia uncl.(54);Clostridia uncl.(54);Clostridia uncl.(54);*
	OTU-2111	0.5445	0.0089	0.6298	0.494	*Firmicutes(100);Clostridia(100);Clostridiales(100);Clostridiales uncl.(100);Clostridiales uncl.(100);*
	OTU-2338	0.5173	0.0055	0.5491	0.494	*Firmicutes(100);Clostridia(100);Clostridiales(100);Ruminococcaceae(100);Ruminococcaceae uncl.(88);*
	OTU-3665	0.5445	0.0095	0.6298	0.494	*Firmicutes(100);Clostridia(100);Clostridiales(100);Ruminococcaceae(100);Faecalibacterium(100);*
	OTU-1344	0.5645	0.0043	0.5491	0.4966	*Firmicutes(100);Clostridia(100);Clostridiales(100);Lachnospiraceae(100);Lachnospiraceae uncl.(100);*
	OTU-2333	0.5645	0.0029	0.5491	0.4966	*Firmicutes(75);Clostridia(75);Clostridiales(75);Clostridiales uncl.(63);Clostridiales uncl.(63);*
	OTU-2423	0.5645	0.0030	0.5491	0.4966	*Bacteria uncl.(75);*
	OTU-4557	0.5645	0.0030	0.5491	0.4966	*Bacteroidetes(100);Bacteroidia(100);Bacteroidales(100);Rikenellaceae(100);Alistipes(100);*
	OTU-5354	0.5645	0.0029	0.5491	0.4966	*Bacteria uncl.(100);*
	OTU-5364	0.5645	0.0064	0.5519	0.4966	*Firmicutes(100);Clostridia(100);Clostridiales(100);Lachnospiraceae(100);Lachnospiraceae uncl.(100);*
	OTU-6037	0.5645	0.0064	0.5519	0.4966	*Firmicutes(100);Clostridia(100);Clostridiales(100);Ruminococcaceae(100);Faecalibacterium(100);*
	OTU-6053	0.5645	0.0030	0.5491	0.4966	*Firmicutes(100);Clostridia(100);Clostridiales(100);Lachnospiraceae(100);Lachnospiraceae uncl.(67);*
	OTU-7634	0.5645	0.0062	0.5519	0.4966	*Firmicutes(100);Clostridia(100);Clostridiales(100);Lachnospiraceae(100);Lachnospiraceae uncl.(100);*
	OTU-7793	0.5645	0.0031	0.5491	0.4966	*Firmicutes(100);Clostridia(100);Clostridiales(100);Ruminococcaceae(100);Faecalibacterium(100);*
	OTU-7796	0.5645	0.0029	0.5491	0.4966	*Firmicutes(100);Clostridia(100);Clostridiales(100);Lachnospiraceae(100);Lachnospiraceae uncl.(100);*
	OTU-8614	0.5645	0.0031	0.5491	0.4966	*Firmicutes(100);Clostridia(100);Clostridiales(100);Ruminococcaceae(100);Faecalibacterium(100);*
	OTU-8639	0.5645	0.0033	0.5491	0.4966	*Bacteria uncl.(100);*
	OTU-8693	0.5645	0.0032	0.5491	0.4966	*Firmicutes(100);Clostridia(100);Clostridiales(100);Clostridiales uncl.(100);Clostridiales uncl.(100);*
	OTU-8784	0.5645	0.0067	0.5591	0.4966	*Firmicutes(100);Clostridia(100);Clostridiales(100);Ruminococcaceae(100);Ruminococcaceae uncl.(100);*
	OTU-1610	0.5208	0.0089	0.6298	0.4974	*Firmicutes(100);Clostridia(100);Clostridiales(100);Clostridiales uncl.(100);Clostridiales uncl.(100);*
	OTU-1670	0.5433	0.0034	0.5491	0.4979	*Firmicutes(100);Clostridia(100);Clostridiales(100);Clostridiales uncl.(93);Clostridiales uncl.(93);*
	OTU-1956	0.5066	0.0039	0.5491	0.4979	*Firmicutes(91);Clostridia(91);Clostridiales(91);Clostridiales uncl.(82);Clostridiales uncl.(82);*
	OTU-1957	0.5542	0.0045	0.5491	0.4979	*Firmicutes(100);Clostridia(100);Clostridiales(100);Ruminococcaceae(100);Ruminococcaceae uncl.(100);*
	OTU-2219	0.5542	0.0046	0.5491	0.4979	*Firmicutes(100);Clostridia(100);Clostridiales(100);Ruminococcaceae(100);Faecalibacterium(100);*
	OTU-3348	0.5542	0.0043	0.5491	0.4979	*Bacteria uncl.(80);*
	OTU-3811	0.5542	0.0049	0.5491	0.4979	*Firmicutes(100);Clostridia(100);Clostridiales(100);Ruminococcaceae(100);Faecalibacterium(100);*
	OTU-1846	0.5891	0.0095	0.6298	0.5192	*Firmicutes(100);Clostridia(100);Clostridiales(100);Lachnospiraceae(100);Lachnospiraceae uncl.(100);*
	OTU-561	0.5770	0.0016	0.5491	0.5674	*Bacteroidetes(100);Bacteroidia(100);Bacteroidales(100);Rikenellaceae(100);Alistipes(100);*
	OTU-2779	0.6138	0.0053	0.5491	0.5679	*Firmicutes(100);Negativicutes(100);Selenomonadales(100);Veillonellaceae(100);Dialister(100);*
	OTU-1045	0.4822	0.0007	0.5491	0.5775	*Bacteroidetes(100);Bacteroidia(100);Bacteroidales(100);Rikenellaceae(100);Alistipes(100);*
MCV (fl)	OTU-1282	0.7216	0.0003	0.5052	-0.5908	*Firmicutes(100);Clostridia(100);Clostridiales(100);Lachnospiraceae(100);Lachnospiraceae uncl.(80);*
	OTU-1109	0.7611	0.0012	0.9998	-0.5027	*Bacteroidetes(100);Bacteroidia(100);Bacteroidales(100);Bacteroidaceae(100);Bacteroides(100);*
	OTU-3426	0.7736	0.0019	0.9998	-0.5027	*Bacteroidetes(100);Bacteroidia(100);Bacteroidales(100);Bacteroidaceae(100);Bacteroides(100);*
	OTU-2772	0.7480	0.0031	0.9998	-0.5021	*Firmicutes(100);Clostridia(100);Clostridiales(100);Ruminococcaceae(100);Ruminococcaceae uncl.(100);*
	OTU-2480	0.6599	0.0062	0.9998	-0.5007	*Firmicutes(100);Clostridia(100);Clostridiales(100);Lachnospiraceae(100);Lachnospiraceae uncl.(100);*
	OTU-1163	0.7352	0.0004	0.5052	-0.4958	*Firmicutes(100);Clostridia(100);Clostridiales(100);Lachnospiraceae(100);Lachnospiraceae uncl.(100);*
	OTU-1838	0.6847	0.0050	0.9998	-0.4668	*Bacteroidetes(100);Bacteroidia(100);Bacteroidales(100);Rikenellaceae(100);Alistipes(100);*
	OTU-3677	0.5915	0.0048	0.9998	-0.4645	*Bacteroidetes(100);Bacteroidia(100);Bacteroidales(100);Rikenellaceae(100);Alistipes(100);*
	OTU-6489	0.5915	0.0048	0.9998	-0.4645	*Firmicutes(100);Clostridia(100);Clostridiales(100);Lachnospiraceae(100);Lachnospiraceae uncl.(100);*
	OTU-6493	0.5915	0.0044	0.9998	-0.4645	*Firmicutes(100);Clostridia(100);Clostridiales(100);Lachnospiraceae(100);Lachnospiraceae uncl.(100);*
	OTU-1081	0.6796	0.0054	0.9998	-0.4324	*Bacteroidetes(100);Bacteroidia(100);Bacteroidales(100);Bacteroidaceae(100);Bacteroides(100);*
	OTU-2313	0.6459	0.0040	0.9998	-0.3724	*Bacteroidetes(100);Bacteroidia(100);Bacteroidales(100);Bacteroidaceae(100);Bacteroides(100);*
	OTU-269	0.6070	0.0083	0.9998	0.4032	*Firmicutes(100);Clostridia(100);Clostridiales(100);Lachnospiraceae(100);Roseburia(100);*
	OTU-2722	0.6289	0.0064	0.9998	0.598	*Firmicutes(100);Clostridia(100);Clostridiales(100);Clostridiales uncl.(72);Clostridiales uncl.(72);*

Monocytes %	OTU-1887	0.6189	0.0065	0.9484	-0.5409	*Firmicutes(100);Clostridia(100);Clostridiales(100);Lachnospiraceae(100);Eisenbergiella(100);*
	OTU-1109	0.5468	0.0096	0.9484	-0.5333	*Bacteroidetes(100);Bacteroidia(100);Bacteroidales(100);Bacteroidaceae(100);Bacteroides(100);*
	OTU-3426	0.5840	0.0050	0.9484	-0.5333	*Bacteroidetes(100);Bacteroidia(100);Bacteroidales(100);Bacteroidaceae(100);Bacteroides(100);*
	OTU-2772	0.5998	0.0047	0.9484	-0.5325	*Firmicutes(100);Clostridia(100);Clostridiales(100);Ruminococcaceae(100);Ruminococcaceae uncl.(100);*
	OTU-994	0.5316	0.0099	0.9484	0.4159	*Firmicutes(100);Clostridia(100);Clostridiales(100);Ruminococcaceae(60);Ruminococcaceae uncl.(60);*
	OTU-2045	0.5239	0.0090	0.9484	0.4445	*Bacteroidetes(100);Bacteroidia(100);Bacteroidales(100);Porphyromonadaceae(100);Odoribacter(100);*
	OTU-2940	0.5315	0.0094	0.9484	0.4445	*Bacteroidetes(100);Bacteroidia(100);Bacteroidales(100);Rikenellaceae(100);Alistipes(100);*
	OTU-1225	0.5667	0.0090	0.9484	0.4544	*Firmicutes(100);Clostridia(100);Clostridiales(100);Lachnospiraceae(100);Lachnospiraceae uncl.(100);*
	OTU-3120	0.5802	0.0084	0.9484	0.5325	*Firmicutes(100);Clostridia(100);Clostridiales(100);Lachnospiraceae(100);Lachnospiraceae uncl.(100);*
	OTU-4433	0.5802	0.0090	0.9484	0.5325	*Bacteroidetes(100);Bacteroidia(100);Bacteroidales(100);Bacteroidaceae(100);Bacteroides(100);*
	OTU-5690	0.5802	0.0091	0.9484	0.5325	*Firmicutes(100);Clostridia(100);Clostridiales(100);Lachnospiraceae(100);Lachnospiraceae uncl.(100);*
	OTU-6598	0.5802	0.0088	0.9484	0.5325	*Firmicutes(100);Clostridia(100);Clostridiales(100);Lachnospiraceae(100);Lachnospiraceae uncl.(100);*
	OTU-7642	0.5802	0.0092	0.9484	0.5325	*Firmicutes(100);Clostridia(100);Clostridiales(100);Ruminococcaceae(100);Oscillibacter(100);*
	OTU-1727	0.6427	0.0046	0.9484	0.58	*Firmicutes(100);Clostridia(100);Clostridiales(100);Ruminococcaceae(100);Oscillibacter(100);*
	OTU-1010	0.6672	0.0024	0.9484	0.6057	*Firmicutes(100);Clostridia(100);Clostridiales(100);Ruminococcaceae(100);Ruminococcaceae uncl.(97);*
	OTU-1497	0.6302	0.0048	0.9484	0.6525	*Firmicutes(100);Clostridia(100);Clostridiales(100);Lachnospiraceae(100);Lachnospiraceae uncl.(100);*
Neutrophiles %	OTU-392	0.6465	0.0029	0.3174	0.2582	*Firmicutes(100);Clostridia(100);Clostridiales(100);Ruminococcaceae(80);Ruminococcaceae uncl.(70);*
	OTU-1090	0.5868	0.0081	0.4875	0.2596	*Firmicutes(100);Clostridia(100);Clostridiales(100);Ruminococcaceae(100);Oscillibacter(67);*
	OTU-1828	0.5842	0.0086	0.5076	0.2942	*Firmicutes(100);Clostridia(100);Clostridiales(100);Ruminococcaceae(100);Ruminococcaceae uncl.(67);*
	OTU-525	0.6360	0.0027	0.3174	0.3428	*Firmicutes(100);Clostridia(100);Clostridiales(100);Ruminococcaceae(100);Ruminococcaceae uncl.(100);*
	OTU-1740	0.5446	0.0077	0.4781	0.378	*Firmicutes(100);Clostridia(100);Clostridiales(100);Clostridiales uncl.(100);Clostridiales uncl.(100);*
	OTU-3137	0.5446	0.0073	0.4781	0.378	*Firmicutes(100);Clostridia(100);Clostridiales(100);Clostridiales uncl.(100);Clostridiales uncl.(100);*
	OTU-4317	0.5446	0.0069	0.4781	0.378	*Firmicutes(100);Clostridia(100);Clostridiales(100);Clostridiales uncl.(75);Clostridiales uncl.(75);*
	OTU-4470	0.5446	0.0071	0.4781	0.378	*Firmicutes(100);Clostridia(100);Clostridiales(100);Lachnospiraceae(100);Lachnospiraceae uncl.(100);*
	OTU-5250	0.5446	0.0075	0.4781	0.378	*Bacteroidetes(100);Bacteroidia(100);Bacteroidales(100);Prevotellaceae(100);Prevotella(100);*
	OTU-812	0.5791	0.0089	0.5142	0.3817	*Firmicutes(100);Clostridia(100);Clostridiales(100);Ruminococcaceae(100);Ruminococcaceae uncl.(98);*
	OTU-309	0.6430	0.0026	0.3174	0.4162	*Firmicutes(100);Clostridia(99);Clostridiales(99);Ruminococcaceae(96);Ruminococcaceae uncl.(96);*
	OTU-393	0.6042	0.0074	0.4781	0.4316	*Firmicutes(100);Clostridia(100);Clostridiales(100);Ruminococcaceae(100);Ruminococcaceae uncl.(100);*
	OTU-1775	0.5578	0.0095	0.5298	0.4387	*Firmicutes(100);Clostridia(100);Clostridiales(100);Ruminococcaceae(100);Ruminococcaceae uncl.(75);*
	OTU-2131	0.5809	0.0096	0.5298	0.4445	*Firmicutes(100);Clostridia(100);Clostridiales(100);Ruminococcaceae(100);Ruminococcaceae uncl.(67);*
	OTU-1126	0.5911	0.0076	0.4781	0.4538	*Firmicutes(100);Clostridia(100);Clostridiales(100);Ruminococcaceae(100);Ruminococcaceae uncl.(76);*
	OTU-755	0.6278	0.0029	0.3174	0.4903	*Bacteroidetes(100);Bacteroidia(100);Bacteroidales(100);Porphyromonadaceae(100);Coprobacter(100);*
	+OTU-187	0.5940	0.0072	0.4781	0.5181	*Firmicutes(100);Clostridia(100);Clostridiales(100);Lachnospiraceae(100);Lachnospiraceae uncl.(100);*
	OTU-215	0.6500	0.0033	0.3441	0.5181	*Firmicutes(100);Clostridia(100);Clostridiales(100);Ruminococcaceae(96);Ruminococcaceae uncl.(96);*
	OTU-2662	0.6292	0.0042	0.3940	0.5181	*Firmicutes(100);Clostridia(100);Clostridiales(100);Ruminococcaceae(100);Ruminococcaceae uncl.(100);*
	OTU-3085	0.5940	0.0069	0.4781	0.5181	*Firmicutes(100);Clostridia(100);Clostridiales(100);Ruminococcaceae(100);Ruminococcaceae uncl.(100);*
	OTU-4062	0.6292	0.0035	0.3508	0.5181	*Firmicutes(100);Negativicutes(100);Selenomonadales(100);Acidaminococcaceae(100);Phascolarctobacterium(100);*
	OTU-3536	0.6583	0.0013	0.2781	0.5203	*Firmicutes(100);Clostridia(100);Clostridiales(100);Ruminococcaceae(100);Ruminococcaceae uncl.(100);*
	OTU-4168	0.6583	0.0014	0.2781	0.5203	*Firmicutes(100);Clostridia(100);Clostridiales(100);Ruminococcaceae(100);Ruminococcaceae uncl.(75);*
	OTU-4316	0.6583	0.0014	0.2781	0.5203	*Firmicutes(100);Clostridia(100);Clostridiales(100);Ruminococcaceae(100);Ruminococcaceae uncl.(100);*
	OTU-4914	0.6583	0.0015	0.2781	0.5203	*Firmicutes(100);Clostridia(100);Clostridiales(100);Lachnospiraceae(100);Clostridium XlVa(67);*
	OTU-5978	0.6583	0.0015	0.2781	0.5203	*Firmicutes(100);Clostridia(100);Clostridiales(100);Ruminococcaceae(100);Butyricicoccus(100);*
	OTU-6195	0.6583	0.0015	0.2781	0.5203	*Firmicutes(100);Clostridia(100);Clostridiales(100);Clostridiales uncl.(100);Clostridiales uncl.(100);*
	OTU-6224	0.6583	0.0016	0.2781	0.5203	*Firmicutes(100);Clostridia(100);Clostridiales(100);Ruminococcaceae(100);Ruminococcaceae uncl.(100);*
	OTU-6452	0.6583	0.0014	0.2781	0.5203	*Firmicutes(100);Clostridia(100);Clostridiales(100);Ruminococcaceae(100);Ruminococcaceae uncl.(100);*
	OTU-6505	0.6583	0.0015	0.2781	0.5203	*Firmicutes(100);Clostridia(100);Clostridiales(100);Ruminococcaceae(100);Ruminococcaceae uncl.(100);*
	OTU-7848	0.6583	0.0016	0.2781	0.5203	*Firmicutes(100);Clostridia(100);Clostridiales(100);Ruminococcaceae(100);Oscillibacter(100);*
	OTU-1992	0.6674	0.0015	0.2781	0.521	*Firmicutes(100);Clostridia(100);Clostridiales(100);Ruminococcaceae(100);Ruminococcaceae uncl.(100);*
	OTU-2529	0.6229	0.0010	0.2781	0.521	*Firmicutes(100);Clostridia(100);Clostridiales(100);Ruminococcaceae(100);Ruminococcaceae uncl.(100);*
	OTU-3212	0.6651	0.0022	0.3174	0.521	*Firmicutes(100);Clostridia(100);Clostridiales(100);Lachnospiraceae(100);Lachnospiraceae uncl.(100);*

	OTU-5781	0.6651	0.0022	0.3174	0.521	*Firmicutes(100);Clostridia(100);Clostridiales(100);Ruminococcaceae(100);Ruminococcaceae uncl.(100);*
	OTU-2103	0.6112	0.0044	0.3940	0.5221	*Firmicutes(100);Clostridia(100);Clostridiales(100);Ruminococcaceae(67);Ruminococcaceae uncl.(67);*
	OTU-6085	0.6112	0.0044	0.3940	0.5221	*Firmicutes(100);Clostridia(100);Clostridiales(100);Ruminococcaceae(100);Oscillibacter(100);*
	OTU-1238	0.6121	0.0062	0.4781	0.5485	*Firmicutes(100);Clostridia(100);Clostridiales(100);Ruminococcaceae(100);Faecalibacterium(96);*
	OTU-990	0.6727	0.0013	0.2781	0.5629	*Firmicutes(100);Clostridia(100);Clostridiales(100);Ruminococcaceae(97);Ruminococcaceae uncl.(97);*
	OTU-1969	0.6024	0.0052	0.4355	0.5666	*Firmicutes(100);Clostridia(100);Clostridiales(100);Ruminococcaceae(100);Ruminococcaceae uncl.(90);*
	OTU-2084	0.6035	0.0056	0.4567	0.5696	*Firmicutes(100);Clostridia(100);Clostridiales(100);Clostridiales Incertae Sedis XIII(90);Clostridiales Incertae Sedis XIII uncl.(90);*
	OTU-3790	0.6288	0.0021	0.3174	0.5707	*Firmicutes(100);Clostridia(100);Clostridiales(100);Ruminococcaceae(100);Faecalibacterium(100);*
	OTU-3005	0.6449	0.0024	0.3174	0.5739	*Firmicutes(100);Clostridia(100);Clostridiales(100);Ruminococcaceae(100);Oscillibacter(84);*
	OTU-2658	0.6602	0.0012	0.2781	0.5751	*Bacteria uncl.(100);*
	OTU-2998	0.6585	0.0013	0.2781	0.5751	*Firmicutes(100);Clostridia(100);Clostridiales(100);Ruminococcaceae(100);Faecalibacterium(84);*
	OTU-878	0.6470	0.0028	0.3174	0.5757	*Firmicutes(100);Clostridia(100);Clostridiales(100);Ruminococcaceae(100);Ruminococcaceae uncl.(100);*
	OTU-2108	0.6026	0.0047	0.4068	0.5872	*Firmicutes(100);Clostridia(100);Clostridiales(100);Ruminococcaceae(100);Ruminococcaceae uncl.(100);*
Thrombocytes (cells/nl)	OTU-744	0.6657	0.0006	0.7643	-0.6723	*Firmicutes(100);Clostridia(100);Clostridiales(100);Ruminococcaceae(100);Faecalibacterium(100);*
	OTU-9	0.6022	0.0060	0.9600	-0.5912	*Firmicutes(100);Clostridia(100);Clostridiales(100);Ruminococcaceae(100);Faecalibacterium(100);*
	OTU-2686	0.5891	0.0020	0.8852	-0.5908	*Bacteroidetes(100);Bacteroidia(100);Bacteroidales(100);Rikenellaceae(100);Alistipes(100);*
	OTU-1000	0.5855	0.0067	0.9600	-0.5541	*Firmicutes(100);Clostridia(100);Clostridiales(100);Ruminococcaceae(100);Faecalibacterium(100);*
	OTU-2304	0.5983	0.0046	0.9600	-0.5201	*Firmicutes(100);Clostridia(100);Clostridiales(100);Ruminococcaceae(100);Ruminococcaceae uncl.(100);*
	OTU-4411	0.5542	0.0085	0.9600	-0.5115	*Firmicutes(100);Clostridia(100);Clostridiales(100);Lachnospiraceae(100);Lachnospiraceae uncl.(100);*
	OTU-4067	0.4917	0.0083	0.9600	-0.499	*Bacteroidetes(100);Bacteroidia(100);Bacteroidales(100);Rikenellaceae(100);Alistipes(100);*
	OTU-8049	0.4960	0.0065	0.9600	-0.4984	*Firmicutes(100);Clostridia(100);Clostridiales(100);Lachnospiraceae(100);Lachnospiraceae uncl.(100);*
	OTU-57	0.5789	0.0074	0.9600	-0.4272	*Firmicutes(100);Clostridia(100);Clostridiales(100);Ruminococcaceae(100);Faecalibacterium(100);*
	OTU-900	0.5691	0.0036	0.9600	0.0939	*Bacteroidetes(100);Bacteroidia(100);Bacteroidales(100);Bacteroidaceae(100);Bacteroides(100);*
	OTU-1729	0.5697	0.0049	0.9600	0.4394	*Firmicutes(100);Clostridia(100);Clostridiales(100);Ruminococcaceae(100);Ruminococcaceae uncl.(100);*
	OTU-244	0.4366	0.0040	0.9600	0.4607	*Firmicutes(100);Clostridia(100);Clostridiales(100);Ruminococcaceae(99);Ruminococcaceae uncl.(99);*
	OTU-5311	0.4974	0.0062	0.9600	0.4752	*Firmicutes(100);Clostridia(100);Clostridiales(100);Ruminococcaceae(100);Ruminococcaceae uncl.(100);*
	OTU-1725	0.5847	0.0017	0.8757	0.4984	*Firmicutes(100);Clostridia(100);Clostridiales(100);Ruminococcaceae(100);Ruminococcaceae uncl.(100);*
	‡OTU-1104	0.6266	0.0012	0.8757	0.5312	*Firmicutes(100);Clostridia(100);Clostridiales(100);Ruminococcaceae(100);Faecalibacterium(100);*
	OTU-2586	0.5571	0.0087	0.9600	0.5533	*Firmicutes(100);Clostridia(100);Clostridiales(100);Ruminococcaceae(86);Ruminococcaceae uncl.(86);*
	‡OTU-1636	0.5951	0.0002	0.5182	0.5964	*Firmicutes(100);Clostridia(100);Clostridiales(100);Ruminococcaceae(100);Ruminococcaceae uncl.(100);*
	‡OTU-2082	0.6435	0.0015	0.8757	0.6377	*Firmicutes(100);Clostridia(100);Clostridiales(100);Lachnospiraceae(100);Lachnospiraceae uncl.(70);*

+ indicators for healthy individuals; # indicators for CRMO; ‡ indicators for relapse; † indicators for no relapse

Table S7: Species level OTUs from CRMO oral communities associated to clinical measurements via Euclidean distance correlation and Spearman rank correlation (SCC-Spearman correlation coefficient). Indicators for healthy individuals are marked with +; # indicators for CRMO; ‡ indicators for relapse; † indicators for no relapse. HACEK-group members are highlighted in **bold** face and red indicates OTUs with multiple associations. The *P*-value cutoff was set to *P*≤0.010.

Physiology	ID	*Eucl. dist. corr.*	*P*	P_{FDR}	*SCC*	RDP 16 Classification
AP (U/l)	OTU-284	0.6939	0.0037	1.0000	0.5298	*Firmicutes(100);Bacilli(100);Lactobacillales(100);Streptococcaceae(100);Streptococcus(100);*
	OTU-1053	0.6829	0.0018	1.0000	0.6276	*Bacteroidetes(100);Bacteroidia(100);Bacteroidales(100);Porphyromonadaceae(100);Porphyromonas(100);*
Basophiles %	OTU-353	0.6986	0.0045	0.7160	0.1635	*Actinobacteria(100);Actinobacteria(100);Actinomycetales(100);Corynebacteriaceae(94);Corynebacterium(93);*
	OTU-1506	0.6228	0.0067	0.7160	0.2082	*Bacteroidetes(100);Bacteroidia(100);Bacteroidales(100);Porphyromonadaceae(100);Porphyromonas(94);*
	**OTU-166**	0.6063	0.0086	0.7160	0.3167	***Proteobacteria(100);Betaproteobacteria(100);Neisseriales(100);Neisseriaceae(100);Kingella(100);***
	OTU-2118	0.6581	0.0087	0.7160	0.3800	*Bacteroidetes(100);Bacteroidia(100);Bacteroidales(100);Prevotellaceae(100);Prevotella(100);*
	OTU-122	0.6584	0.0093	0.7160	0.3934	*Firmicutes(100);Bacilli(100);Lactobacillales(100);Streptococcaceae(100);Lactococcus(100);*
	**OTU-400**	0.7687	0.0019	0.7160	0.4327	***Proteobacteria(100);Betaproteobacteria(100);Neisseriales(100);Neisseriaceae(100);Kingella(100);***
	OTU-5017	0.6775	0.0051	0.7160	0.4963	*Actinobacteria(100);Actinobacteria(100);Actinomycetales(100);Corynebacteriaceae(100);Corynebacterium(100);*
BSR (1h)	OTU-3017	0.5305	0.0086	0.9593	-0.5461	*Firmicutes(100);Bacilli(100);Lactobacillales(100);Streptococcaceae(100);Streptococcus(100);*
	OTU-88	0.5897	0.0061	0.9593	-0.4354	*Fusobacteria(99);Fusobacteriia(99);Fusobacteriales(99);Leptotrichiaceae(97);Leptotrichiaceae uncl.(95);*
	OTU-97	0.5616	0.0032	0.9593	0.3466	*Firmicutes(100);Bacilli(100);Lactobacillales(100);Streptococcaceae(100);Streptococcus(100);*
	OTU-2175	0.5253	0.0093	0.9593	0.4181	*Firmicutes(100);Bacilli(100);Lactobacillales(100);Streptococcaceae(100);Streptococcus(100);*
	**OTU-4203**	0.5202	0.0050	0.9593	0.4742	***Proteobacteria(100);Gammaproteobacteria(100);Pasteurellales(100);Pasteurellaceae(100);Pasteurellaceae uncl.(100);***
	OTU-423	0.5979	0.0008	0.9186	0.5335	*Firmicutes(100);Bacilli(100);Lactobacillales(100);Streptococcaceae(100);Streptococcus(100);*
	OTU-5263	0.5460	0.0058	0.9593	0.5436	*Fusobacteria(100);Fusobacteriia(100);Fusobacteriales(100);Leptotrichiaceae(100);Leptotrichia(100);*
Calcium (mmol/l)	OTU-848	0.5875	0.0071	0.3377	-0.6101	*Bacteroidetes(100);Bacteroidetes uncl.(66);Bacteroidetes uncl.(66);Bacteroidetes uncl.(66);Bacteroidetes uncl.(66);*
	OTU-7	0.6467	0.0022	0.2706	-0.4745	*Firmicutes(100);Bacilli(100);Bacillales(100);Bacillales_Incertae_Sedis_XI(100);Gemella(100);*
	OTU-206	0.6095	0.0044	0.2706	-0.4421	*Firmicutes(100);Bacilli(100);Lactobacillales(100);Streptococcaceae(100);Streptococcus(100);*
	OTU-62	0.6103	0.0041	0.2706	0.1568	*Bacteroidetes(100);Bacteroidia(100);Bacteroidales(100);Porphyromonadaceae(100);Porphyromonas(100);*
	OTU-518	0.6215	0.0050	0.2706	0.2987	*Firmicutes(100);Bacilli(100);Lactobacillales(100);Streptococcaceae(100);Streptococcus(100);*
	OTU-811	0.6407	0.0037	0.2706	0.3829	*Bacteroidetes(100);Bacteroidia(100);Bacteroidales(100);Porphyromonadaceae(100);Porphyromonas(100);*
	**OTU-2222**	0.5992	0.0074	0.3377	0.3864	***Proteobacteria(100);Gammaproteobacteria(100);Pasteurellales(100);Pasteurellaceae(100);Pasteurellaceae uncl.(78);***
	**OTU-2634**	0.5831	0.0086	0.3542	0.4051	***Proteobacteria(100);Gammaproteobacteria(100);Pasteurellales(100);Pasteurellaceae(100);Pasteurellaceae uncl.(100);***
	OTU-281	0.6556	0.0010	0.2048	0.4658	*Fusobacteria(100);Fusobacteriia(100);Fusobacteriales(100);Leptotrichiaceae(100);Leptotrichia(100);*
	OTU-4561	0.5913	0.0080	0.3421	0.4721	*Proteobacteria(100);Betaproteobacteria(100);Betaproteobacteria uncl.(100);Betaproteobacteria uncl.(100);Betaproteobacteria uncl.(100);*
	**OTU-5093**	0.5913	0.0080	0.3421	0.4721	***Proteobacteria(100);Gammaproteobacteria(100);Pasteurellales(100);Pasteurellaceae(100);Aggregatibacter(67);***
	**OTU-4022**	0.6248	0.0044	0.2706	0.4749	***Proteobacteria(100);Gammaproteobacteria(100);Pasteurellales(100);Pasteurellaceae(100);Pasteurellaceae uncl.(75);***
	OTU-4767	0.6248	0.0046	0.2706	0.4749	*Actinobacteria(100);Actinobacteria(100);Actinomycetales(100);Actinomycetales uncl.(100);Actinomycetales uncl.(100);*
	OTU-6679	0.6248	0.0048	0.2706	0.4749	*Proteobacteria(100);Betaproteobacteria(100);Neisseriales(100);Neisseriaceae(100);Neisseriaceae uncl.(100);*
	OTU-2701	0.6265	0.0040	0.2706	0.4766	*Bacteroidetes(100);Bacteroidia(100);Bacteroidales(100);Porphyromonadaceae(100);Porphyromonas(100);*
	OTU-2857	0.6404	0.0026	0.2706	0.4766	*Bacteroidetes(100);Bacteroidia(100);Bacteroidales(100);Porphyromonadaceae(100);Porphyromonas(100);*
	OTU-4664	0.6514	0.0042	0.2706	0.4766	*Bacteroidetes(100);Bacteroidia(100);Bacteroidales(100);Porphyromonadaceae(100);Porphyromonas(67);*
	OTU-5130	0.6514	0.0040	0.2706	0.4766	*Bacteria uncl.(100);Bacteria uncl.(100);Bacteria uncl.(100);Bacteria uncl.(100);Bacteria uncl.(100);*
	OTU-2924	0.6534	0.0004	0.1193	0.4999	*Bacteroidetes(100);Bacteroidetes uncl.(100);Bacteroidetes uncl.(100);Bacteroidetes uncl.(100);Bacteroidetes uncl.(100);*
	OTU-3625	0.6534	0.0003	0.1193	0.4999	*Fusobacteria(100);Fusobacteriia(100);Fusobacteriales(100);Fusobacteriaceae(100);Fusobacterium(100);*
	OTU-6818	0.6534	0.0005	0.1193	0.4999	*Firmicutes(100);Bacilli(100);Lactobacillales(100);Streptococcaceae(100);Streptococcus(100);*
	OTU-7985	0.6534	0.0004	0.1193	0.4999	*Proteobacteria(100);Betaproteobacteria(100);Neisseriales(100);Neisseriaceae(100);Neisseriaceae uncl.(100);*
	OTU-1358	0.6421	0.0022	0.2706	0.5023	*Bacteroidetes(100);Flavobacteriia(100);Flavobacteriales(100);Flavobacteriaceae(100);Capnocytophaga(100);*
	OTU-1705	0.6357	0.0049	0.2706	0.5193	*Actinobacteria(100);Actinobacteria(100);Actinomycetales(100);Actinomycetales uncl.(100);Actinomycetales uncl.(100);*

	OTU-541	0.6053	0.0058	0.3024	0.5217	Bacteria uncl.(100);Bacteria uncl.(100);Bacteria uncl.(100);Bacteria uncl.(100);Bacteria uncl.(100);
	OTU-2359	0.5737	0.0068	0.3365	0.5589	Fusobacteria(88);Fusobacteriia(88);Fusobacteriales(88);Leptotrichiaceae(88);Leptotrichiaceae uncl.(88);
	OTU-2852	0.7067	0.0004	0.1193	0.5669	Bacteroidetes(100);Bacteroidia(100);Bacteroidales(100);Porphyromonadaceae(100);Porphyromonas(100);
	OTU-431	0.6422	0.0023	0.2706	0.5670	SR1(100);SR1 uncl.(100);SR1 uncl.(100);SR1 uncl.(100);SR1 uncl.(100);
	OTU-2727	0.6194	0.0030	0.2706	0.6054	Actinobacteria(100);Actinobacteria(100);Actinomycetales(100);Actinomycetaceae(100);Actinomyces(100);
CRP (mg/l)	#OTU-1136	0.5896	0.0080	0.9995	0.6244	Actinobacteria(100);Actinobacteria(100);Actinomycetales(100);Micrococcaceae(100);Rothia(100);
Eosinophiles %	†OTU-1346	0.6511	0.0038	0.8203	0.3518	Bacteroidetes(74);Bacteroidetes uncl.(74);Bacteroidetes uncl.(74);Bacteroidetes uncl.(74);Bacteroidetes uncl.(74);
	OTU-1700	0.6435	0.0084	0.8203	0.4404	Bacteroidetes(100);Flavobacteriia(100);Flavobacteriales(100);Flavobacteriaceae(100);Flavobacteriaceae uncl.(100);
	**OTU-3170**	0.5990	0.0082	0.8203	0.4705	**Proteobacteria(100);Gammaproteobacteria(100);Pasteurellales(100);Pasteurellaceae(100);Haemophilus(100);**
	**OTU-860**	0.6492	0.0081	0.8203	0.4743	**Proteobacteria(100);Gammaproteobacteria(100);Pasteurellales(100);Pasteurellaceae(100);Haemophilus(100);**
	OTU-3775	0.6492	0.0085	0.8203	0.4743	Proteobacteria(100);Betaproteobacteria(100);Neisseriales(100);Neisseriaceae(100);Neisseriaceae uncl.(100);
	OTU-4173	0.6492	0.0083	0.8203	0.4743	Bacteria uncl.(100);Bacteria uncl.(100);Bacteria uncl.(100);Bacteria uncl.(100);Bacteria uncl.(100);
	OTU-6299	0.6492	0.0085	0.8203	0.4743	Fusobacteria(100);Fusobacteriia(100);Fusobacteriales(100);Leptotrichiaceae(100);Leptotrichia(100);
	OTU-8459	0.6492	0.0086	0.8203	0.4743	Proteobacteria(100);Betaproteobacteria(100);Burkholderiales(100);Burkholderiaceae(100);Lautropia(100);
	**OTU-1116**	0.6480	0.0040	0.8203	0.4761	**Proteobacteria(100);Betaproteobacteria(100);Neisseriales(100);Neisseriaceae(100);Kingella(100);**
	**OTU-836**	0.6654	0.0026	0.8203	0.5269	**Proteobacteria(100);Gammaproteobacteria(100);Pasteurellales(100);Pasteurellaceae(100);Pasteurellaceae uncl.(89);**
	OTU-1	0.5982	0.0057	0.8203	0.5436	Firmicutes(100);Bacilli(100);Lactobacillales(100);Streptococcaceae(100);Streptococcus(100);
	OTU-1372	0.5706	0.0093	0.8203	0.5463	Firmicutes(100);Bacilli(100);Lactobacillales(100);Streptococcaceae(100);Streptococcus(100);
	OTU-179	0.6223	0.0036	0.8203	0.5475	Firmicutes(100);Bacilli(100);Lactobacillales(100);Streptococcaceae(100);Streptococcus(100);
Erythrocytes (cells/pl)	OTU-1158	0.6547	0.0008	0.9067	-0.5915	Bacteroidetes(100);Bacteroidia(100);Bacteroidales(100);Porphyromonadaceae(100);Porphyromonas(100);
	OTU-2511	0.5539	0.0097	0.9589	-0.4826	Bacteroidetes(100);Flavobacteriia(86);Flavobacteriales(86);Flavobacteriaceae(86);Flavobacteriaceae uncl.(86);
	OTU-7953	0.6216	0.0018	0.9589	-0.4794	Proteobacteria(100);Betaproteobacteria(100);Neisseriales(100);Neisseriaceae(100);Neisseriaceae uncl.(100);
	**OTU-5870**	0.5424	0.0096	0.9589	-0.4644	**Proteobacteria(100);Gammaproteobacteria(100);Pasteurellales(100);Pasteurellaceae(100);Haemophilus(100);**
	OTU-9203	0.5740	0.0059	0.9589	0.4794	Actinobacteria(100);Actinobacteria(100);Actinomycetales(100);Actinomycetaceae(100);Actinomyces(100);
	OTU-1269	0.5840	0.0055	0.9589	0.4823	Actinobacteria(100);Actinobacteria(100);Actinomycetales(100);Actinomycetaceae(100);Actinomyces(100);
Hb (g/dl)	OTU-1096	0.5657	0.0069	0.9863	-0.5660	Bacteroidetes(100);Bacteroidia(100);Bacteroidales(100);Porphyromonadaceae(100);Porphyromonas(100);
	OTU-1571	0.6448	0.0020	0.9863	-0.5044	Firmicutes(100);Bacilli(100);Lactobacillales(100);Streptococcaceae(100);Streptococcus(100);
	**OTU-6277**	0.6889	0.0031	0.9863	-0.4798	**Proteobacteria(100);Gammaproteobacteria(100);Pasteurellales(100);Pasteurellaceae(100);Pasteurellaceae uncl.(100);**
	**OTU-5911**	0.5486	0.0094	0.9863	-0.4738	**Proteobacteria(100);Gammaproteobacteria(100);Pasteurellales(100);Pasteurellaceae(100);Pasteurellaceae uncl.(100);**
	OTU-1782	0.7076	0.0033	0.9863	-0.4593	Firmicutes(100);Bacilli(100);Lactobacillales(100);Carnobacteriaceae(100);Granulicatella(100);
	**OTU-1375**	0.6041	0.0034	0.9863	-0.4214	**Proteobacteria(100);Gammaproteobacteria(100);Pasteurellales(100);Pasteurellaceae(100);Pasteurellaceae uncl.(95);**
Hematocrit	OTU-1312	0.5810	0.0038	0.9718	-0.5666	Firmicutes(100);Bacilli(100);Lactobacillales(100);Streptococcaceae(100);Streptococcus(100);
	**OTU-6277**	0.5973	0.0007	0.7874	-0.4842	**Proteobacteria(100);Gammaproteobacteria(100);Pasteurellales(100);Pasteurellaceae(100);Pasteurellaceae uncl.(100);**
	**OTU-1375**	0.5611	0.0053	0.9718	-0.4365	**Proteobacteria(100);Gammaproteobacteria(100);Pasteurellales(100);Pasteurellaceae(100);Pasteurellaceae uncl.(95);**
	OTU-1782	0.5907	0.0066	0.9718	-0.4214	Firmicutes(100);Bacilli(100);Lactobacillales(100);Carnobacteriaceae(100);Granulicatella(100);
	**OTU-6685**	0.5895	0.0071	0.9718	0.4842	**Proteobacteria(100);Gammaproteobacteria(100);Pasteurellales(100);Pasteurellaceae(100);Pasteurellaceae uncl.(100);**
LDH (U/l)	OTU-527	0.6314	0.0029	0.7471	-0.6813	Proteobacteria(100);Betaproteobacteria(100);Neisseriales(100);Neisseriaceae(100);Neisseria(99);
	OTU-336	0.5589	0.0046	0.7471	-0.0343	Candidatus_Saccharibacteria(100);Candidatus_Saccharibacteria uncl.(100)
	OTU-406	0.5938	0.0050	0.7471	-0.0033	Fusobacteria(95);Fusobacteriia(95);Fusobacteriales(95);Fusobacteriales uncl.(72);Fusobacteriales uncl.(72);
	OTU-169	0.5806	0.0049	0.7471	0.1277	Bacteroidetes(100);Bacteroidia(100);Bacteroidales(100);Prevotellaceae(99);Prevotellaceae uncl.(97);
	OTU-5032	0.6126	0.0066	0.7898	0.4738	Fusobacteria(100);Fusobacteriia(100);Fusobacteriales(100);Fusobacteriaceae(100);Fusobacterium(100);
	OTU-5350	0.6126	0.0066	0.7898	0.4738	Bacteria uncl.(100);Bacteria uncl.(100);Bacteria uncl.(100);Bacteria uncl.(100);Bacteria uncl.(100);
	OTU-3096	0.6315	0.0034	0.7471	0.4987	Proteobacteria(100);Betaproteobacteria(100);Burkholderiales(100);Burkholderiaceae(100);Lautropia(100);
	**OTU-4576**	0.6315	0.0034	0.7471	0.4987	**Proteobacteria(100);Gammaproteobacteria(100);Pasteurellales(100);Pasteurellaceae(100);Haemophilus(67);**
	**OTU-8980**	0.6315	0.0036	0.7471	0.4987	**Proteobacteria(100);Gammaproteobacteria(100);Pasteurellales(100);Pasteurellaceae(100);Haemophilus(100);**
	+OTU-548	0.6439	0.0011	0.7471	0.5944	Bacteroidetes(100);Bacteroidia(99);Bacteroidales(99);Prevotellaceae(93);Prevotellaceae uncl.(93);
Lymphocytes %	**OTU-1307**	0.5746	0.0086	0.3130	-0.5790	**Proteobacteria(100);Gammaproteobacteria(100);Pasteurellales(100);Pasteurellaceae(100);Haemophilus(65);**
	OTU-2733	0.6372	0.0033	0.3130	-0.4984	Actinobacteria(100);Actinobacteria(100);Actinomycetales(100);Actinomycetaceae(100);Actinomyces(100);
	OTU-162	0.5170	0.0064	0.3130	0.1523	Actinobacteria(100);Actinobacteria(100);Actinomycetales(100);Actinomycetaceae(100);Actinomyces(100);

	OTU-104	0.6638	0.0017	0.3130	0.2811	*Fusobacteria(100);Fusobacteriia(100);Fusobacteriales(100);Fusobacteriaceae(100);Fusobacterium(100);*
	OTU-727	0.5284	0.0094	0.3130	0.3321	*Firmicutes(100);Clostridia(100);Clostridiales(100);Lachnospiraceae(100);Catonella(100);*
	OTU-2931	0.5885	0.0065	0.3130	0.3344	*Actinobacteria(100);Actinobacteria(100);Actinomycetales(100);Actinomycetaceae(100);Actinomyces(100);*
	OTU-806	0.6457	0.0008	0.3130	0.3843	*Proteobacteria(100);Gammaproteobacteria(100);Pseudomonadales(100);Pseudomonadaceae(100);Pseudomonas(100);*
	OTU-539	0.6166	0.0069	0.3130	0.3901	*Firmicutes(100);Bacilli(100);Bacillales(100);Staphylococcaceae(100);Staphylococcus(100);*
	OTU-353	0.5895	0.0034	0.3130	0.3972	*Actinobacteria(100);Actinobacteria(100);Actinomycetales(100);Corynebacteriaceae(94);Corynebacterium(93);*
	OTU-1107	0.6642	0.0015	0.3130	0.4122	*Firmicutes(100);Negativicutes(100);Selenomonadales(100);Veillonellaceae(100);Veillonella(100);*
	OTU-698	0.6722	0.0005	0.3130	0.4288	*Firmicutes(100);Clostridia(100);Clostridiales(100);Lachnospiraceae(100);Catonella(100);*
	OTU-1383	0.6198	0.0028	0.3130	0.4349	*Firmicutes(100);Negativicutes(100);Selenomonadales(100);Veillonellaceae(100);Selenomonas(100);*
	#OTU-255	0.5997	0.0040	0.3130	0.4540	*Proteobacteria(100);Epsilonproteobacteria(100);Campylobacterales(100);Campylobacteraceae(100);Campylobacter(100);*
	OTU-132	0.6299	0.0023	0.3130	0.4623	*Proteobacteria(100);Epsilonproteobacteria(100);Campylobacterales(100);Campylobacteraceae(100);Campylobacter(100);*
	OTU-1524	0.6246	0.0021	0.3130	0.4847	*Fusobacteria(100);Fusobacteriia(100);Fusobacteriales(100);Fusobacteriaceae(100);Fusobacterium(100);*
	OTU-736	0.5521	0.0095	0.3130	0.4967	*Firmicutes(100);Bacilli(100);Lactobacillales(100);Lactobacillaceae(100);Lactobacillus(100);*
	OTU-908	0.5832	0.0084	0.3130	0.4967	*Firmicutes(100);Bacilli(100);Bacillales(100);Staphylococcaceae(100);Staphylococcus(100);*
	OTU-5867	0.6171	0.0054	0.3130	0.4984	*Firmicutes(100);Bacilli(100);Lactobacillales(100);Streptococcaceae(100);Streptococcus(100);*
	OTU-774	0.5385	0.0032	0.3130	0.4990	*Proteobacteria(100);Gammaproteobacteria(100);Oceanospirillales(100);Alcanivoracaceae(100);Alcanivorax(100);*
	OTU-823	0.4897	0.0090	0.3130	0.4990	*Bacteroidetes(100);Sphingobacteriia(100);Sphingobacteriales(100);Chitinophagaceae(100);Chitinophagaceae uncl.(100);*
	OTU-1527	0.6182	0.0054	0.3130	0.4990	*Firmicutes(100);Negativicutes(100);Selenomonadales(100);Veillonellaceae(100);Dialister(100);*
	OTU-2442	0.5293	0.0028	0.3130	0.4990	*Firmicutes(88);Firmicutes uncl.(88);Firmicutes uncl.(88);Firmicutes uncl.(88);Firmicutes uncl.(88);*
	OTU-819	0.5588	0.0100	0.3130	0.5138	*Firmicutes(100);Negativicutes(100);Selenomonadales(100);Veillonellaceae(100);Selenomonas(100);*
	OTU-1659	0.6389	0.0021	0.3130	0.6119	*Proteobacteria(100);Betaproteobacteria(100);Neisseriales(100);Neisseriaceae(100);Neisseriaceae uncl.(93);*
MCH (pg/cell)	**OTU-1375**	0.5997	0.0028	0.5627	-0.6267	***Proteobacteria(100);Gammaproteobacteria(100);Pasteurellales(100);Pasteurellaceae(100);Pasteurellaceae uncl.(95);***
	OTU-1782	0.5831	0.0018	0.4366	-0.4785	*Firmicutes(100);Bacilli(100);Lactobacillales(100);Carnobacteriaceae(100);Granulicatella(100);*
	OTU-4038	0.5526	0.0060	0.8006	0.4658	*Actinobacteria(100);Actinobacteria(100);Actinomycetales(100);Actinomycetaceae(100);Actinomyces(100);*
	OTU-6710	0.5526	0.0054	0.8006	0.4658	*Firmicutes(100);Bacilli(100);Lactobacillales(100);Streptococcaceae(100);Streptococcus(100);*
	OTU-7315	0.5526	0.0054	0.8006	0.4658	*Actinobacteria(100);Actinobacteria(100);Actinomycetales(100);Corynebacteriaceae(100);Corynebacterium(100);*
	OTU-5400	0.5743	0.0017	0.4366	0.4891	*Firmicutes(100);Bacilli(100);Lactobacillales(100);Streptococcaceae(100);Streptococcus(100);*
	OTU-6956	0.5743	0.0014	0.4366	0.4891	*Actinobacteria(100);Actinobacteria(100);Actinomycetales(100);Actinomycetaceae(100);Actinomyces(100);*
	OTU-2511	0.5353	0.0099	0.9182	0.4969	*Bacteroidetes(100);Flavobacteriia(86);Flavobacteriales(86);Flavobacteriaceae(86);Flavobacteriaceae uncl.(86);*
	**#OTU-835**	0.6564	0.0004	0.4366	0.4975	***Proteobacteria(100);Gammaproteobacteria(100);Cardiobacteriales(100);Cardiobacteriaceae(100);Cardiobacterium(100);***
	OTU-2168	0.5481	0.0086	0.9182	0.5119	*Firmicutes(100);Bacilli(100);Lactobacillales(100);Streptococcaceae(100);Streptococcus(100);*
	OTU-3982	0.6000	0.0012	0.4366	0.5580	*Bacteria uncl.(75);Bacteria uncl.(75);Bacteria uncl.(75);Bacteria uncl.(75);Bacteria uncl.(75);*
MCHC (g/dl)	OTU-86	0.5752	0.0055	0.5313	0.0064	*Bacteroidetes(100);Flavobacteriia(100);Flavobacteriales(100);Flavobacteriaceae(100);Flavobacteriaceae uncl.(100);*
	**#OTU-512**	0.6284	0.0020	0.5313	0.2608	***Proteobacteria(100);Betaproteobacteria(100);Neisseriales(100);Neisseriaceae(100);Kingella(100);***
	**#OTU-740**	0.5665	0.0062	0.5326	0.3472	***Proteobacteria(100);Gammaproteobacteria(100);Pasteurellales(100);Pasteurellaceae(100);Pasteurellaceae uncl.(92);***
	OTU-237	0.5564	0.0050	0.5313	0.3514	*Actinobacteria(100);Actinobacteria(100);Actinomycetales(100);Corynebacteriaceae(100);Corynebacterium(100);*
	**#OTU-835**	0.5841	0.0047	0.5313	0.4164	***Proteobacteria(100);Gammaproteobacteria(100);Cardiobacteriales(100);Cardiobacteriaceae(100);Cardiobacterium(100);***
	OTU-2235	0.5747	0.0052	0.5313	0.4166	*Firmicutes(100);Bacilli(100);Lactobacillales(100);Streptococcaceae(100);Streptococcus(100);*
	OTU-4038	0.5678	0.0051	0.5313	0.4777	*Actinobacteria(100);Actinobacteria(100);Actinomycetales(100);Actinomycetaceae(100);Actinomyces(100);*
	OTU-6710	0.5678	0.0036	0.5313	0.4777	*Firmicutes(100);Bacilli(100);Lactobacillales(100);Streptococcaceae(100);Streptococcus(100);*
	OTU-7315	0.5678	0.0041	0.5313	0.4777	*Actinobacteria(100);Actinobacteria(100);Actinomycetales(100);Corynebacteriaceae(100);Corynebacterium(100);*
	OTU-377	0.5687	0.0005	0.3102	0.4792	*Bacteroidetes(100);Flavobacteriia(100);Flavobacteriales(100);Flavobacteriaceae(100);Capnocytophaga(100);*
	OTU-1968	0.5705	0.0058	0.5313	0.5223	*Fusobacteria(100);Fusobacteriia(100);Fusobacteriales(100);Leptotrichiaceae(100);Leptotrichia(100);*
	OTU-3775	0.6308	0.0043	0.5313	0.5264	*Proteobacteria(100);Betaproteobacteria(100);Neisseriales(100);Neisseriaceae(100);Neisseriaceae uncl.(100);*
	**OTU-1116**	0.5697	0.0014	0.5313	0.5274	***Proteobacteria(100);Betaproteobacteria(100);Neisseriales(100);Neisseriaceae(100);Kingella(100);***
	**OTU-5446**	0.7042	0.0004	0.3102	0.5849	***Proteobacteria(100);Gammaproteobacteria(100);Pasteurellales(100);Pasteurellaceae(100);Pasteurellaceae uncl.(100);***
MCV (fl)	**OTU-562**	0.6249	0.0025	0.8808	-0.3852	***Proteobacteria(100);Gammaproteobacteria(100);Pasteurellales(100);Pasteurellaceae(100);Haemophilus(89);***
	OTU-2359	0.5628	0.0091	0.8808	0.5702	*Fusobacteria(88);Fusobacteriia(88);Fusobacteriales(88);Leptotrichiaceae(88);Leptotrichiaceae uncl.(88);*
	**OTU-1965**	0.5869	0.0096	0.8808	0.6020	***Proteobacteria(100);Gammaproteobacteria(100);Pasteurellales(100);Pasteurellaceae(100);Haemophilus(55);***
Monocytes	OTU-520	0.5981	0.0060	0.8266	-0.5933	*Firmicutes(100);Bacilli(100);Lactobacillales(100);Streptococcaceae(100);Streptococcus(100);*

%	OTU-5285	0.5705	0.0092	0.8266	-0.5395	*Firmicutes(100);Clostridia(100);Clostridiales(100);Clostridiales uncl.(100);Clostridiales uncl.(100);*
	OTU-4038	0.4808	0.0099	0.8266	0.3694	*Actinobacteria(100);Actinobacteria(100);Actinomycetales(100);Actinomycetaceae(100);Actinomyces(100);*
	OTU-386	0.4935	0.0065	0.8266	0.4588	*Bacteroidetes(100);Bacteroidia(100);Bacteroidales(100);Prevotellaceae(100);Prevotella(100);*
	OTU-3980	0.5530	0.0043	0.8266	0.5098	*Firmicutes(100);Bacilli(100);Lactobacillales(100);Streptococcaceae(100);Streptococcus(100);*
	OTU-843	0.5248	0.0011	0.8266	0.5238	*Proteobacteria(100);Betaproteobacteria(100);Burkholderiales(100);Comamonadaceae(100);Ottowia(100);*
	OTU-1984	0.5952	0.0052	0.8266	0.5787	*Actinobacteria(100);Actinobacteria(100);Actinomycetales(100);Actinomycetaceae(100);Actinomyces(100);*
Neutrophiles %	OTU-2316	0.4982	0.0089	0.8932	0.3612	*Firmicutes(100);Negativicutes(100);Selenomonadales(100);Veillonellaceae(100);Selenomonas(100);*
	**OTU-4203**	0.4982	0.0086	0.8932	0.3612	***Proteobacteria(100);Gammaproteobacteria(100);Pasteurellales(100);Pasteurellaceae(100);Pasteurellaceae uncl.(100);***
	OTU-5263	0.4982	0.0086	0.8932	0.3612	*Fusobacteria(100);Fusobacteriia(100);Fusobacteriales(100);Leptotrichiaceae(100);Leptotrichia(100);*
	OTU-2733	0.5623	0.0080	0.8932	0.4735	*Actinobacteria(100);Actinobacteria(100);Actinomycetales(100);Actinomycetaceae(100);Actinomyces(100);*
	**OTU-1307**	0.6130	0.0031	0.8932	0.5913	***Proteobacteria(100);Gammaproteobacteria(100);Pasteurellales(100);Pasteurellaceae(100);Haemophilus(65);***
Thrombocytes (cells/nl)	OTU-1571	0.5630	0.0042	0.9990	-0.5614	*Firmicutes(100);Bacilli(100);Lactobacillales(100);Streptococcaceae(100);Streptococcus(100);*
	**OTU-2203**	0.5813	0.0031	0.9990	-0.5356	***Proteobacteria(100);Gammaproteobacteria(100);Pasteurellales(100);Pasteurellaceae(100);Pasteurellaceae uncl.(67);***
	**OTU-1833**	0.4661	0.0087	0.9990	-0.4587	***Proteobacteria(100);Gammaproteobacteria(100);Pasteurellales(100);Pasteurellaceae(100);Haemophilus(59);***
	**OTU-4396**	0.5062	0.0023	0.9990	0.4778	***Proteobacteria(100);Gammaproteobacteria(100);Pasteurellales(100);Pasteurellaceae(100);Pasteurellaceae uncl.(100);***
	OTU-4812	0.5519	0.0017	0.9990	0.4791	*Bacteria uncl.(100);Bacteria uncl.(100);Bacteria uncl.(100);Bacteria uncl.(100);Bacteria uncl.(100);*
	OTU-1901	0.4790	0.0056	0.9990	0.4938	*Firmicutes(100);Bacilli(100);Lactobacillales(100);Streptococcaceae(100);Streptococcus(100);*
	OTU-1964	0.5105	0.0086	0.9990	0.5177	*Bacteroidetes(100);Bacteroidia(100);Bacteroidales(100);Porphyromonadaceae(100);Porphyromonas(100);*

+ indicators for healthy individuals; # indicators for CRMO; ‡ indicators for relapse; † indicators for no relapse

Table S8: Association of alpha diversity measures in the fecal and oral microbial communities with the clinical measurements in CRMO patients. Lines highlighted in red show nominally significant Euclidean distance correlations and includes Spearman rank correlation coefficients (SCC).

		Stool					Saliva				
Diversity	Physiology	N	SCC	Euclidean dist.	*P*-value	P_{FDR}	N	SCC	Euclidean dist.	*P*-value	P_{FDR}
ACE species richness	BSR (1h)	21	-0.0559	0.3283	0.3817	0.8589	22	-0.1255	0.3438	0.4368	0.7863
	CRP (mg/l)	22	-0.0017	0.3019	0.4892	0.8594	24	0.0762	0.2566	0.8531	0.8531
	Hb (g/dl)	22	-0.1125	0.3528	0.3510	0.8589	24	-0.0300	0.3175	0.6684	0.8531
	Erythrocytes (cells/pl)	22	-0.2796	0.3841	0.2199	0.7171	24	-0.2645	0.3259	0.5690	0.8531
	Hematocrit	22	-0.3340	0.4118	0.1459	0.6565	24	-0.1837	0.3669	0.3417	0.6833
	MCV (fl)	22	-0.0040	0.2796	0.8387	0.9127	24	0.3425	0.4837	0.0467	0.2803
	MCH (pg/cell)	21	0.0989	0.2896	0.6684	0.8594	23	0.3685	0.4348	0.1171	0.3719
	MCHC (g/dl)	21	0.3006	0.2771	0.6480	0.8594	23	0.2638	0.3849	0.2383	0.6127
	Thrombocytes (cells/nl)	22	0.0141	0.1917	0.9971	0.9971	24	-0.1087	0.2866	0.6857	0.8531
	Neutrophiles %	20	0.4090	0.5303	0.0325	0.3490	22	-0.3315	0.3679	0.2841	0.6393
	Eosinophiles %	20	-0.0542	0.2585	0.8513	0.9127	22	-0.1657	0.2968	0.6595	0.8531
	Basophiles %	19	-0.1565	0.3998	0.2390	0.7171	21	-0.5186	0.4358	0.1240	0.3719
	Lymphocytes %	20	-0.3624	0.5245	0.0388	0.3490	22	0.3721	0.4603	0.0882	0.3719
	Monocytes %	19	0.1632	0.3149	0.6389	0.8594	21	0.0760	0.2890	0.8158	0.8531
	Calcium (mmol/l)	20	-0.0437	0.3062	0.6578	0.8594	22	0.5210	0.6073	0.0055	0.0986
	AP (U/l)	12	-0.4343	0.4456	0.4971	0.8594	13	-0.0275	0.3844	0.7870	0.8531
	LDH (U/l)	20	-0.2701	0.4241	0.1399	0.6565	22	-0.0028	0.3141	0.7169	0.8531
Shannon H numbers equivalent	BSR (1h)	21	-0.0351	0.3184	0.4781	0.9201	22	-0.0729	0.2900	0.8097	0.8757
	CRP (mg/l)	22	0.0793	0.3072	0.5149	0.9201	24	-0.1415	0.2679	0.8169	0.8757
	Hb (g/dl)	22	-0.1035	0.3549	0.3970	0.9201	24	0.0017	0.3140	0.7093	0.8757
	Erythrocytes (cells/pl)	22	-0.2559	0.3814	0.2613	0.7839	24	-0.0840	0.3420	0.4901	0.8757
	Hematocrit	22	-0.3506	0.4154	0.1566	0.6466	24	0.0325	0.3430	0.4979	0.8757
	MCV (fl)	22	-0.1314	0.2892	0.8466	0.9201	24	0.2692	0.4082	0.2029	0.6087
	MCH (pg/cell)	21	0.0507	0.2944	0.7136	0.9201	23	0.2132	0.2938	0.7993	0.8757
	MCHC (g/dl)	21	0.2778	0.2534	0.8572	0.9201	23	0.0228	0.2408	0.9694	0.9694
	Thrombocytes (cells/nl)	22	-0.0141	0.2321	0.9650	0.9650	24	0.0565	0.2680	0.8271	0.8757
	Neutrophiles %	20	0.4045	0.5358	0.0328	0.4090	22	-0.4613	0.4268	0.1491	0.5368
	Eosinophiles %	20	-0.0783	0.2708	0.8434	0.9201	22	-0.1946	0.3320	0.5233	0.8757
	Basophiles %	19	-0.0584	0.4297	0.1796	0.6466	21	-0.3448	0.4653	0.0966	0.4347
	Lymphocytes %	20	-0.3669	0.5197	0.0454	0.4090	22	0.4873	0.5616	0.0156	0.1215
	Monocytes %	19	0.1272	0.3370	0.5784	0.9201	21	-0.2592	0.3764	0.3754	0.8757
	Calcium (mmol/l)	20	0.0430	0.3029	0.7321	0.9201	22	0.5447	0.6305	0.0029	0.0522
	AP (U/l)	12	-0.4273	0.4356	0.5724	0.9201	13	0.2971	0.3949	0.7675	0.8757
	LDH (U/l)	20	-0.3115	0.4463	0.1128	0.6466	22	0.1333	0.3316	0.6065	0.8757

Table S9: Association of beta diversity measures of fecal and oral microbial communities with clinical measurements in CRMO patients via PERMANOVA. Red highlights nominally significant associations between beta diversity and clinical measurements.

		Fecal					Oral				
Metric	Physiology	DF	*F*-Value	*P*-Value	R^2	Adj. R^2	DF	*F*-Value	*P*-Value	R^2	Adj. R^2
Bray-Curtis	BSR (1h)	1,19	1.1024	0.2328	0.0548	0.0051	1,20	0.9939	0.4437	0.0473	-0.0003
	CRP (mg/l)	1,20	0.7561	0.9649	0.0364	-0.0117	1,22	0.7064	0.9654	0.0311	-0.0129
	Hb (g/dl)	1,20	1.1671	0.1566	0.0551	0.0079	1,22	0.8006	0.8363	0.0351	-0.0087
	Erythrocytes (cells/pl)	1,20	1.2890	0.0521	0.0605	0.0136	1,22	0.8824	0.6999	0.0386	-0.0051
	Hematocrit*	1,20	1.4467	0.0117	0.0675	0.0208	1,22	1.0149	0.4221	0.0441	0.0006
	MCV (fl)	1,20	0.9022	0.6997	0.0432	-0.0047	1,22	1.0232	0.3949	0.0444	0.0010
	MCH (pg/cell)	1,19	1.0333	0.3674	0.0516	0.0017	1,21	0.8198	0.8096	0.0376	-0.0083
	MCHC (g/dl)	1,19	1.2005	0.1127	0.0594	0.0099	1,21	0.8299	0.8007	0.0380	-0.0078
	Thrombocytes (cells/nl)	1,20	0.9441	0.5962	0.0451	-0.0027	1,22	0.7970	0.8749	0.0350	-0.0089
	Neutrophils %	1,18	1.1276	0.1970	0.0590	0.0067	1,20	1.0905	0.2690	0.0517	0.0043
	Eosinophils %	1,18	0.9606	0.5602	0.0507	-0.0021	1,20	1.4950	0.0404	0.0696	0.0230
	Basophils%	1,17	0.8545	0.8055	0.0479	-0.0081	1,19	0.9761	0.4788	0.0489	-0.0012
	Lymphocytes %	1,18	1.1550	0.1633	0.0603	0.0081	1,20	1.2480	0.1215	0.0587	0.0117
	Monocytes %	1,17	0.9903	0.4731	0.0550	-0.0005	1,19	0.9142	0.5924	0.0459	-0.0043
	Calcium (mmol/l)	1,18	0.9991	0.4635	0.0526	0.0000	1,20	1.5541	0.0240	0.0721	0.0257
	AP (U/l)	1,10	1.1507	0.1808	0.1032	0.0135	1,11	0.8865	0.7492	0.0746	-0.0095
	LDH (U/l)	1,18	0.9483	0.5821	0.0500	-0.0027	1,20	0.8935	0.6631	0.0428	-0.0051
	Age	1,20	0.9207	0.6637	0.0440	-0.0038	1,22	1.6499	0.0128	0.0698	0.0275
Jaccard	BSR (1h)	1,19	0.9898	0.6670	0.0495	-0.0005	1,20	0.9733	0.7149	0.0464	-0.0013
	CRP (mg/l)	1,20	0.9719	0.9291	0.0463	-0.0013	1,22	0.9027	0.9964	0.0394	-0.0043
	Hb (g/dl)	1,20	1.0065	0.3442	0.0479	0.0003	1,22	0.9552	0.8263	0.0416	-0.0020
	Erythrocytes (cells/pl)	1,20	1.0146	0.2112	0.0483	0.0007	1,22	0.9706	0.7149	0.0423	-0.0013
	Hematocrit	1,20	1.0305	0.0909	0.0490	0.0015	1,22	0.9550	0.8389	0.0416	-0.0020
	MCV (fl)	1,20	1.0275	0.1151	0.0489	0.0013	1,22	1.0659	0.0940	0.0462	0.0029
	MCH (pg/cell)	1,19	0.9939	0.5795	0.0497	-0.0003	1,21	1.0543	0.1277	0.0478	0.0025
	MCHC (g/dl)	1,19	1.0268	0.1038	0.0513	0.0013	1,21	0.9708	0.6970	0.0442	-0.0013
	Thrombocytes (cells/nl)	1,20	0.9985	0.4743	0.0475	-0.0001	1,22	0.9957	0.4770	0.0433	-0.0002
	Neutrophils %	1,18	1.0175	0.1739	0.0535	0.0009	1,20	0.9809	0.6193	0.0468	-0.0009
	Eosinophils %	1,18	0.9976	0.5093	0.0525	-0.0001	1,20	1.0345	0.2209	0.0492	0.0016
	Basophils %	1,17	1.0105	0.2753	0.0561	0.0006	1,19	1.0872	0.0547	0.0541	0.0043
	Lymphocytes %	1,18	1.0302	0.0812	0.0541	0.0016	1,20	1.0452	0.1631	0.0497	0.0021

Monocytes %	1,17	1.0005	0.4463	0.0556	0.0000	1,19	1.0129	0.3264	0.0506	0.0006
Calcium (mmol/l)	1,18	0.9605	0.9848	0.0507	-0.0021	1,20	1.0985	0.0319	0.0521	0.0047
AP (U/l)	1,10	1.0311	0.0653	0.0935	0.0028	1,11	0.9442	0.9036	0.0790	-0.0047
LDH (U/l)	1,18	1.0063	0.3224	0.0529	0.0003	1,20	1.0234	0.2621	0.0487	0.0011
Age	1,20	0.9771	0.9049	0.0466	-0.0011	1,22	1.2387	0.0012	0.0533	0.0103
